# The genetic component of preeclampsia: A whole-exome sequencing study

**DOI:** 10.1371/journal.pone.0197217

**Published:** 2018-05-14

**Authors:** Anette Tarp Hansen, Jens Magnus Bernth Jensen, Anne-Mette Hvas, Mette Christiansen

**Affiliations:** 1 Department of Clinical Biochemistry, Aarhus University Hospital, Aarhus, Denmark; 2 Department of Clinical Biochemistry, Aalborg University Hospital, Aalborg, Denmark; 3 Department of Clinical Medicine, Health, Aarhus University, Aarhus, Denmark; 4 Department of Clinical Immunology, Aarhus University Hospital, Aarhus, Denmark; German Cancer Research Center (DKFZ), GERMANY

## Abstract

Preeclampsia is a major cause of maternal and perinatal deaths. The aetiology of preeclampsia is largely unknown but a polygenetic component is assumed. To explore this hypothesis, we performed an in-depth whole-exome sequencing study in women with (cases, N = 50) and without (controls, N = 50) preeclampsia. The women were identified in an unselected cohort of 2,545 pregnant women based on data from the Danish National Patient Registry and the Medical Birth Registry. Matching DNA was obtained from a biobank containing excess blood from routine antenatal care visits. Novogene performed the whole-exome sequencing blinded to preeclampsia status. Variants for comparison between cases and controls were filtered in the Ingenuity Variant Analysis software. We applied two different strategies; a *disease association panel* approach, which included variants in single genes associated with established clinical risk factors for preeclampsia, and a *gene panel* approach, which included biological pathways harbouring genes previously reported to be associated with preeclampsia. Variant variability was compared in cases and controls at the level of biological processes, signalling pathways, and in single genes. Regardless of the applied strategy and the level of variability examined, we consistently found positive correlations between variant numbers in cases and controls (all R^2^s>0.88). Contrary to what was expected, cases carried fewer variants in biological processes and signalling pathways than controls (all *p*-values ≤0.02). In conclusion, our findings challenge the hypothesis of a polygenetic aetiology for preeclampsia with a common network of susceptibility genes. The greater genetic diversity among controls may suggest a protective role of genetic diversity against the development of preeclampsia.

## Introduction

Preeclampsia is a leading cause of maternal and perinatal deaths with estimated 343,000 women worldwide dying from preeclampsia in the last decennium [1]. In survivors, preeclampsia is associated with an increased risk of premature death from any cause, cardiovascular disease, and with adverse pregnancy outcomes in future pregnancies, including preeclampsia and impaired foetal growth requiring premature induction of delivery [2–6]. Preeclampsia affects 3–8% of pregnancies with an increasing incidence, probably due to an increased burden of maternal obesity and diabetes, and the trend of postponing pregnancy to higher maternal ages [7–10]. Identifying women at risk of developing preeclampsia enables early preventive treatment with low-dose aspirin [11].

The aetiology of preeclampsia is poorly understood, however, defect placentation with impaired utero-placental flow plays an essential role [9,12]. Disturbances in placental growth factors and regulators of angiogenesis, and reduced immune tolerance to “non-self” tissue in the placenta and the foetus are additional suggested mechanisms for preeclampsia [7,13]. Clustering of preeclampsia cases within families suggests a genetic etiological component from maternal, foetal, and/or paternal genes [9,14–16]. Women with an affected first relative are at three to five-fold increased risk of developing preeclampsia themselves [9,16,17] and within some families, preeclampsia seems to follow Mendelian patterns for disease inheritance of rare deleterious genetic variants [15,18]. Family studies based on large cohorts of affected women and relatives have suggested that variation in activin A receptor type 2A (*ACVR2)*, rho associated coiled-coil containing protein kinase 2 (*ROCK2)*, endoplasmic reticulum aminopeptidase 1 (*ERAP1)*, and endoplasmic reticulum aminopeptidase 2 (*ERAP2)* genes are associated with preeclampsia [19–21], reviewed in [14]. However, for the majority of preeclampsia cases, the genetic contribution seems more complex and likely polygenetic [14,17]. This was supported by twin studies showing discordance in preeclampsia phenotype in monozygotic twin pairs, suggesting only minor genetic contribution [22,23]. Therefore, the findings from family studies may not be generalizable to the overall preeclampsia population [14,22,23].

Previous candidate gene studies and genome-wide association studies have chosen candidate genes based on the existent knowledge. More than 50 candidate genes for preeclampsia within various pathophysiological paths have been suggested, but no universally accepted susceptibility genes for preeclampsia have yet been identified [14]. Since preeclampsia probably has a polygenetic aetiology of rare genetic variants, a high-resolution systematic investigation of the whole exome is needed [24,25]. Kaartokallio et al. used pooled blood samples for an exome sequencing study, thus comparing the pooled frequency of gene variants to reference data. The authors concluded that no genetic variants reached statistically significance for preeclampsia [24]. However, this design rendered the origin of genetic variants blurred, *i*.*e*., the reported variants could be clustered within few individuals or the other extreme be spread across individuals. Thus, transparent whole-exome investigations on single women are warranted to further unravel the genetic contribution in preeclampsia.

Yet, there is no published whole exome sequencing study for preeclampsia in the literature. Here, we report the first whole-exome sequencing study on blood samples from preeclampsia cases and controls, allowing direct comparison of the genetic variability in cases and controls.

## Materials and methods

### Setting

In Denmark, nearly all pregnant women attend a routine antenatal care visit at their general practitioner during early first trimester. Upon this first trimester visit, the general practitioner obtains a blood sample for maternal blood typing and screening for human immunodeficiency virus, hepatitis B virus, and syphilis.

In the period from May 2014 to June 2015, we collected all first trimester blood samples received for analysis every second day at the Department of Clinical Immunology, Aarhus University Hospital, Denmark (N = 2,545 women). The present study was based on these samples. EDTA stabilized whole blood was centrifuged, aliquoted in plasma and cell pellet, and stored at -80°C until genomic analysis.

We obtained data on the course of the 2,545 pregnancies from the Danish National Patient Registry [26] and the Medical Birth Registry [27]. Those born in or immigrating to Denmark receives a unique Civil Personal Registration number, enabling accurate linkage among Danish registries at the individual level [28]. The Danish National Patient Registry has recorded data on all admissions and discharges from Danish non-psychiatric hospitals according to the *International Classification of Diseases*, *Eighth Revision (ICD-8)* from 1977 until the end of 1993 and *Tenth Revision (ICD-10)* thereafter [26]. Each hospital discharge or outpatient visit is coded in the Danish National Patient Registry. The Medical Birth Registry contains prospectively collected data on all deliveries in Denmark since 1 January 1973 [27].

### Study population

From the Danish National Patient Registry and the Medical Birth Registry, we identified women registered with a preeclampsia diagnosis among the 2,545 pregnancies. Danish preeclampsia patients are generally diagnosed according to the criteria for preeclampsia stated in the American College of Obstetricians and Gynaecologists’ task force report on hypertension during pregnancy: hypertension (≥ 140/90 mm Hg) debuting from gestational weeks 20 and proteinuria (www.acog.org). In total, we identified 58 women with a preeclampsia diagnosis during index pregnancy). We included the 50 women developing preeclampsia (cases) at the earliest gestational ages. Skjaerven and co-workers previously reported that especially severe cases of preeclampsia seemed to have a genetic component [29]. Early onset of preeclampsia is in clinical practice considered severe preeclampsia. For that reason, we selected the 50 women with the earliest onset of preeclampsia as cases in the present study. These women accounted for 86% of the total number of preeclampsia cases in the entire cohort. The distribution of non-genetic known risk factors for preeclampsia (maternal age, parity, and body mass index; data not shown) did not differ between the subgroup of women included in the present study and the total group of preeclampsia cases. Additional non-genetic risk factors for preeclampsia were un-likely to have played a role for our case-selection and overall conclusions in the study.

Fifty women were randomly included as controls, if they had no history of diabetes, arterial cardiovascular disease, venous thromboembolism, transient ischemic attack, cerebral ischemic stroke, hypertension, acute or chronic renal disease or gestational diabetes diagnosed within the index pregnancy.

### Whole-exome sequencing

Genomic DNA was purified from the cell pellets using the Qiasymphony DNA Midi kit (Qiagen, The Netherlands). The DNA concentration was determined using Qubit Broad Range DNA kit (Thermofisher). Mean concentration was 174 ng/μL (range 27–394 ng/μL). Novogene Bioinformatics Technology Co., Hong Kong, performed the whole-exome sequencing blinded to preeclampsia disease status.

### Post-sequencing bioinformatics

**Fig 1:**
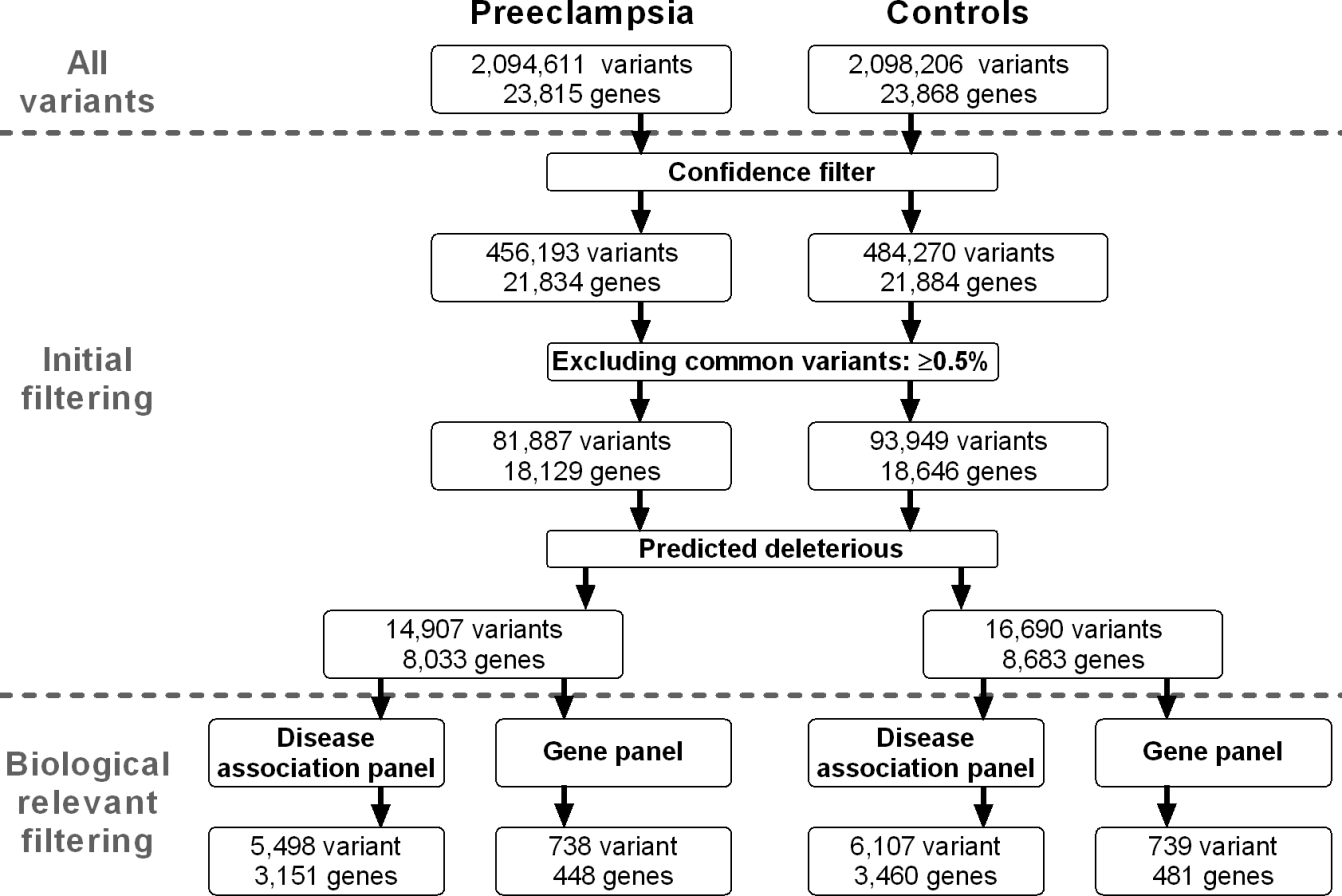
Filtering cascade. Number of variants and genes present after initial filtering and biological filtering.

The variant call files containing variant info were uploaded to Ingenuity Variant Analysis Software (Qiagen), hereafter denominated IVA Software, and filtered as illustrated in Fig 1.

### Initial filtering

First, we filtered for confidence by excluding variants of call quality below 20 (equivalent to base call accuracy of 99%), read depth below 10, allele fraction below 25%, and genotype quality below 30 (equivalent to genotype call accuracy of 99.9%). Subsequently, we excluded common variants. These were defined as variants with reported allele frequencies of 0.5% or greater in one of the following databases: 1000 genomes project [30], NHLBI ESP exomes [31], Allele frequency Community [32], Exome Aggregation Consortium [33] and gnomAD [34]. Variants predicted to be disease associated were included if the following criteria were met: variants classified as pathogenic or likely pathogenic according to computed American College of Medical Genetics and Genomics guidelines [35], disease-associated variants according to the Human Gene Mutation Database [36] or disease-associated variants according to the CLINVAR database [37].

Lastly, variants predicted to be deleterious were included according to the following criteria: frameshift, in-frame insertion and deletions, stop codon changes, missense unless predicted innocuous by SIFT [38] or polyphen-2 [39], CADD score > 15.0 [40] disrupt splice site up to 2 bases into intron, or predicted to disrupt splicing by MaxEntScan [41]. The initial filtering is further specified in S1 Appendix.

### Biological filtering

We employed two different approaches; a *disease association panel* approach and a *gene panel* approach for the filtering according to biological mechanisms (specified in S1 Appendix). In the *disease association panel* approach, we filtered for variants in genes associated with clinical risk factors for preeclampsia [7]. These included genes associated with abnormal immune tolerance, chronic kidney disease, coagulopathy, diabetes, hypertension, preeclampsia, systemic lupus erythematosus, and thrombophilia. In the g*ene panel* approach, we searched for relevant biological pathways harbouring genes previously reported to be protective or disease causing for preeclampsia. These included the renin-angiotensin system pathway, antigen presentation folding, peptide loading of class I major histocompatibility complex, cell adhesion endothelial cell contacts by non-junctional mechanisms, DNA double-strand break repair, complement and coagulation cascades, epoxide hydrolase pathway, myometrial relaxation and contraction pathways, transforming growth factor-beta signalling pathway. In the *gene panel*, we additionally included genes previously reported to be associated with preeclampsia in a large genome wide association study [21]. We exported genes present in these pathways from PathCards [42].

### Biological processes and pathways

We furthermore investigated the genetic variability by comparing the variant frequency for cases and controls separately of the 100 most significant biological processes and pathways, as defined by the IVA software. The IVA software defines biological processes as the biological properties of particular molecules, or the effects that a given molecule has on a disease or function [43]. Based on classical models of signal transduction and information in the Qiagen Knowledge Base [43], the Qiagen’s Content Curation team has defined pathways in the IVA software. The IVA software determined the significance of each particular biological process or pathway by the use of a right-tailed Fisher's exact test, testing if the frequency of genetic variants within these processes and pathways was higher than expected by random chance.

### Statistics

The correlations of variants in the two groups were examined by linear regression, based on least squares residuals. Difference in variant prevalence was assessed using binomial distribution, as the probability of the observed prevalence given prevalence was equal in the groups. We defined the level of significance as 0.05. Raw *p*-values were corrected for multiple comparisons by the Bonferroni approach, unless otherwise stated. Ninety-five percent confidence intervals are presented in brackets []. Data analysis was made in Stata 11.0, StataCorp, USA.

### Ethics

The Ethics Committee of Central Regional Denmark and the Danish Data Protection Agency approved the study (record number/date 1-16-02-294-13/ 20.06.2013 and record number 1-10-72-46-16).

## Results

We collected blood samples from 2,545 unique women attending their first antenatal visit at their general practitioner. Of those, 58 (2.2%) subsequently had a preeclampsia diagnosis registered in the Danish Medical Birth Registry. We included the 50 women who developed preeclampsia at the earliest gestational ages. Table 1 shows demographic characteristics of women developing and not developing preeclampsia.

**Table 1:** Characteristics of women with (cases) and without (controls) preeclampsia.

	Cases (N = 50)		Controls (N = 50)	
	Median	10–90 percentile	Median	10–90 percentile
Gestational age at blood sampling, days	76	69–87	80	66–91
Gestational age at blood sampling, weeks^+days^	10^+6^	–	11^+6^	–
Maternal age at blood sampling, years	25	20–35	30	24–37
Maternal body mass index, kg/m^2^	24	20–33	23	19–29
Gestational age at delivery, days	267	237–282	281	266–291
Birth weight, grams	3,088	1,605–3,770	3,505	2,804–4,140

Women developing preeclampsia were younger, delivered infants with lower birth weights, and had a lower gestational age in comparison to controls. The raw sequencing data obtained in the case and control groups were of similar coverage and quality (Table 2). The *p*-values for all quality parameters were all > 0.1.

**Table 2:** Whole-exome sequencing quality data.

	Cases Mean (SD)	Controls Mean (SD)
Raw data (Gb)	7.7 (0.6)	7.6 (0.7)
Q30 (%)	91.6 (1.1)	91.4 (1.0)
Effective sequences on or near target (Mb)	5,558 (415)	5,461 (443)
Mean sequencing depth on target	70 (5)	68 (6)
Fraction of target covered with at least 20x	0.95 (0.01)	0.95 (0.01)
Total number of SNP	218,254 (14,170)	220,074 (13,638)
Number of transitions	151,996 (9,940)	153,109 (9,500)
Number of transversions	66,257 (4,245)	66,965 (4,150)
Number of novel SNP	17,600 (4,735)	16,497 (3,613)
Number of SNP in CDS	22,883 (888)	23,001 (998)
Number of stop gains	86 (6)	88 (7)
Number of stop loss	12 (2)	12 (2)

SD: standard deviation; Gb: Giga-bases. Q30: the percent of bases with phred-scaled quality scores greater than 30. Mb: Mega-bases

### Comparison of the 100 most variable biological processes in cases and controls

**Fig 2:**
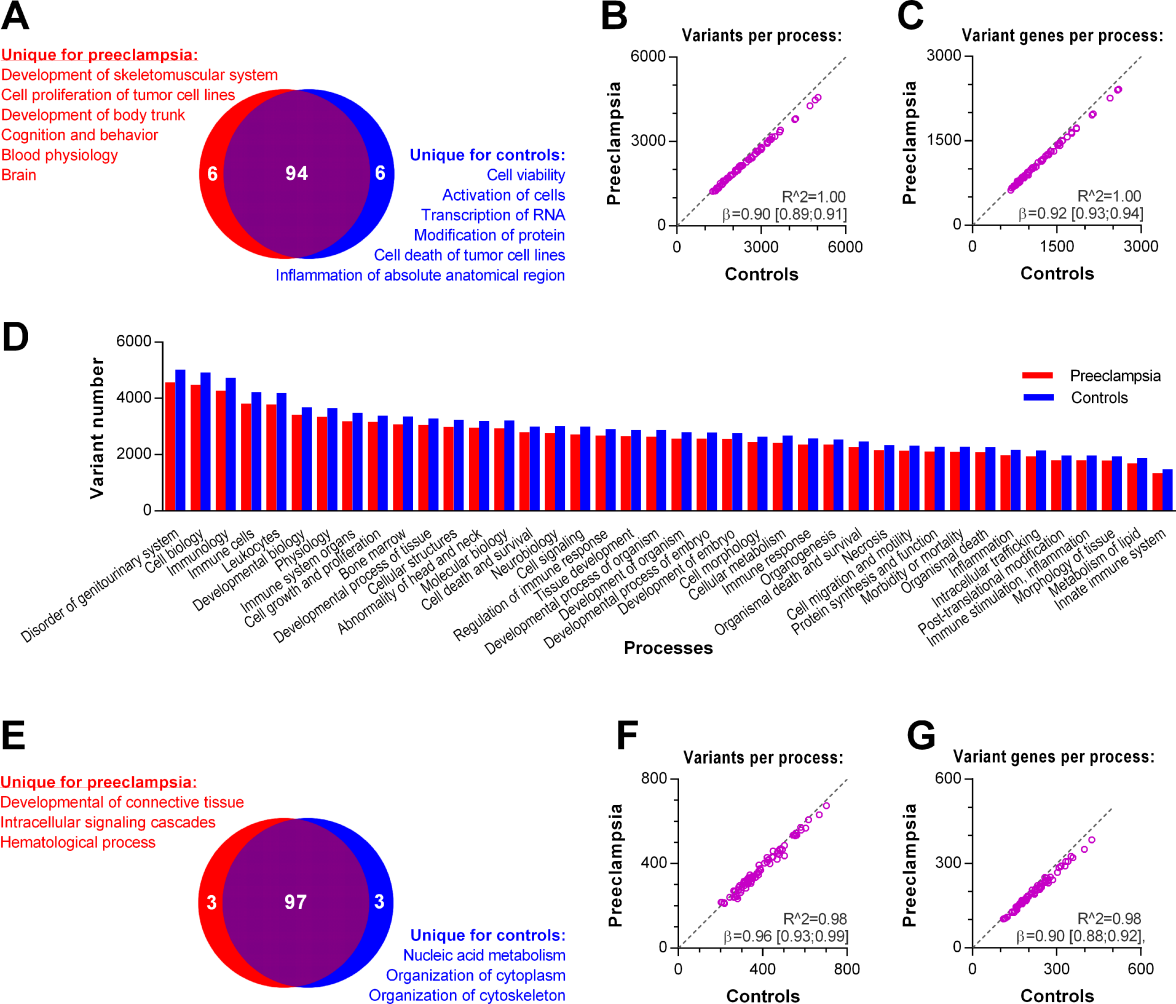
Comparison of the 100 most variable biological processes in cases and controls. Variant processes were identified with the *disease association panel* (**A-D**) or the g*ene panel* (**E**-**G**). **A**: The number of unique and shared variant harbouring processes. For shared variant processes, correlation of total variant number (**B**) and number of genes carrying variants (**C**). Number of variants in 40 processes which less frequently carried variants in cases (**D**). **E**: Number of unique and shared variant processes. For shared variant harbouring processes, correlation of total variant number (**F**) and number of genes carrying variants (**G**).

Using the *disease association panel*, we identified the 100 most variable biological processes in each of the two groups. The two groups shared 94 of these processes (Fig 2A). All women had variants in each of these biological processes. For the shared variant processes, we found a strong and highly significant correlation of total variant number (Fig 2B) and number of genes carrying variants (Fig 2C) across the case and control groups. Cases had 10% [9.4%–11%] fewer variants per biological process and 7.6% [6.8%–8.3%] fewer genes with variants per biological process compared to the control group (*p*<0.0001). To explore this difference further, we identified the biological processes in which variant frequency differed between the two groups. We found 40 different biological processes with a lower number of variants in cases than in controls (Fig 2D).

We repeated these analyses using the g*ene panel*. Even more biological processes, 97 of the 100, were shared for the case and control group (Fig 2E). Again, the two groups correlated strongly in number of variants in single processes (Fig 2F) and the number of genes harbouring variants (Fig 2G). In addition, cases had fewer variants per process (3.9% [1.2%–6.6%]) and fewer genes were affected per process (10% [8.5%–12%]) compared to controls (*p*≤0.005). However, the variant frequency did not differ for any single biological process.

### Comparison of the 100 most variable pathways in cases and controls

**Fig 3:**
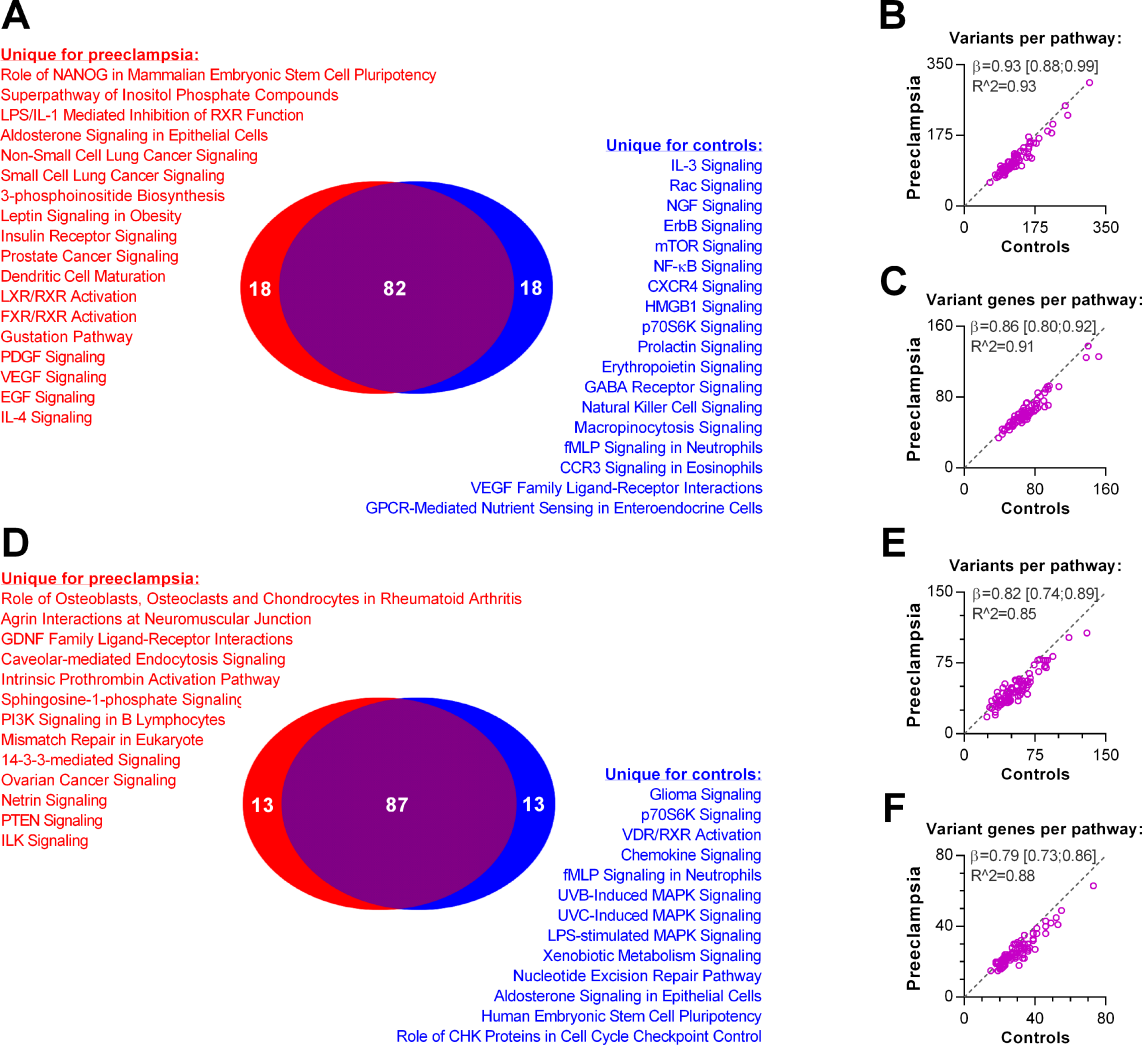
Comparison of the 100 most variable pathways in cases and controls. Variant pathways were identified with the *disease association panel* (**A-C**) or the *gene panel* (**C**-**F**). **A**: Number of unique and shared variant harbouring pathways. For shared variant pathways, correlations of total variant number and number of genes carrying variants are presented in **B** and **C**, respectively. **D**: Number of unique and shared variant harbouring pathways. For shared variant pathways, correlations of total variant number and number of genes carrying variants are presented in **E** and **F**.

We then addressed variability in pathways, as defined in the IVA Software. When using the *disease association panel*, we found that 82 pathways were shared for cases and controls (Fig 3A). Both the number of variants per pathway and the number of genes harbouring variants correlated strongly and significantly in the two groups (Fig 3B and 3C). On average, cases had 6.7% [1.1%–12%] fewer variants per pathway and 14% [7.8%–20%] fewer variant genes per pathway compared to controls (*p* ≤0.02).

When applying the *gene panel* approach, we observed that 87 pathways were shared for cases and controls (Fig 3D). Both the number of variants per pathway and the number of genes harbouring variants correlated strongly in the two groups (Fig 3E and 3F). On average, cases had 18% [11%–26%] fewer variants per pathway and 21% [14%–27%] fewer variant genes per pathway than controls (*p*<0.0001).

### Comparison of variant genes in cases and controls

**Fig 4:**
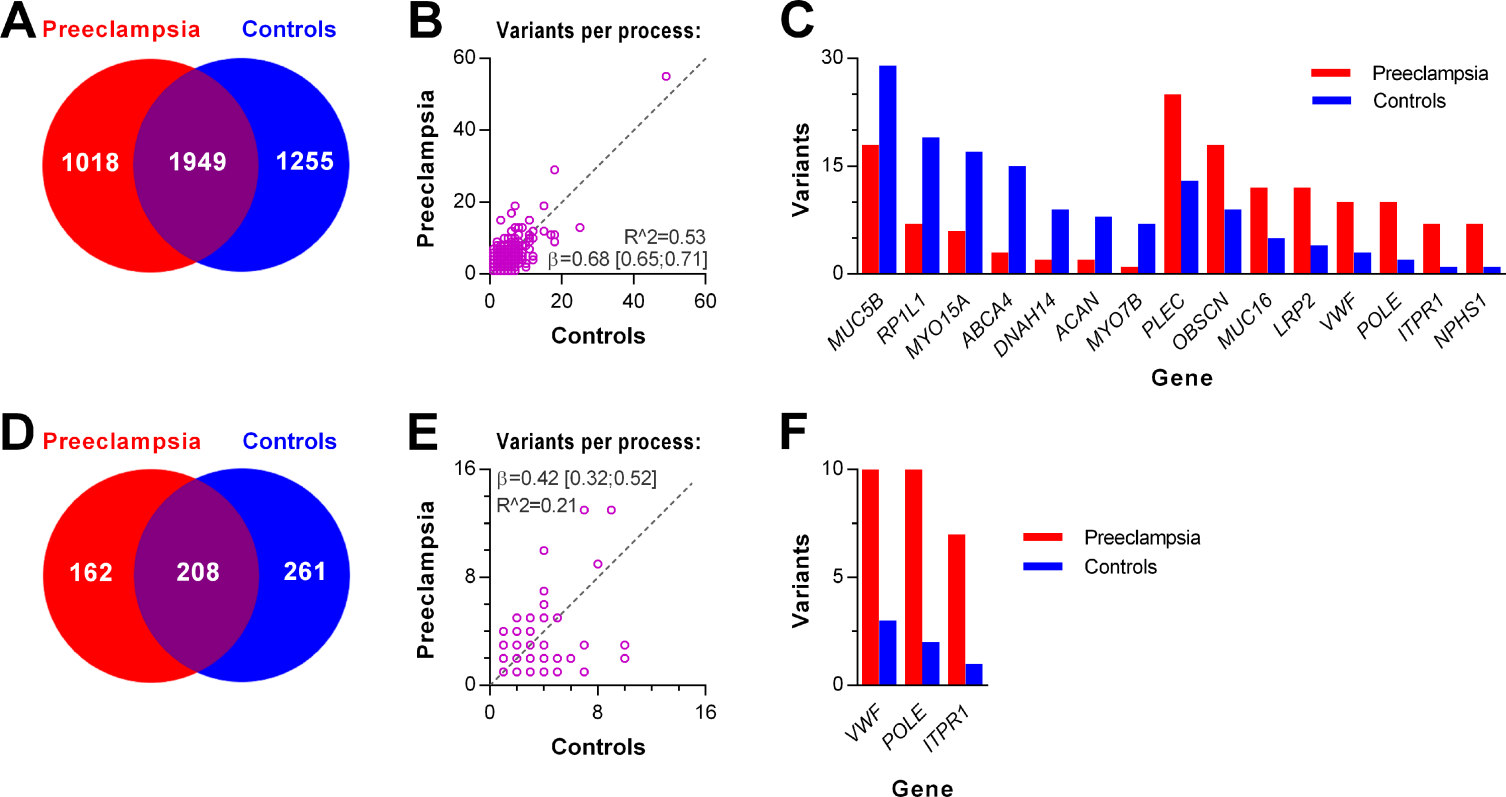
Comparison of variant genes in cases and controls. Genes containing variants were identified with the *disease association panel* (**A-C**) or the *gene panel* (**C**-**F**). **A**: Number of unique and shared variant harbouring genes. For the shared genes, correlation of total variant number is depicted in **B**. **C**: Genes with extreme distribution of variant numbers between groups (uncorrected *p*-values below 0.05). **D**: Number of unique and shared variant harbouring pathways. For the shared genes, correlation of total variant number is depicted in **E**. **F**: Genes with extreme distribution of variant numbers between groups (uncorrected *p*-values below 0.05).

We then studied variants in single genes. Again, we applied the *disease association panel*. Cases had variants in 1,018 genes and controls had variants in 1,255 genes, whereas both groups had variants in 1,949 genes (Fig 4A). The total number of variants in the shared genes again showed a strong and highly significant correlation between the two groups (Fig 4B). Fig 4C depicts raw data on shared genes with the most skewed prevalence of variants (raw *p*-values below 0.05, but without significant difference after Bonferroni correction for multiple comparison). We then applied the g*ene panel*. Cases had variants in 162 genes, controls had variants in 208 genes, whereas both groups shared 261 genes harbouring variants (Fig 4D). The total number of variants in the shared genes correlated strongly between the two groups (Fig 4E) and no difference was observed in variant numbers for any of these genes. Fig 4F depicts raw data on shared genes with the most skewed prevalence of variants (raw *p*-values below 0.05, but without significant difference after Bonferroni correction for multiple comparison).

### Rare deleterious variants more frequent in cases

We performed a sensitivity analysis for uncovering the maximum genetic variability between cases and controls. We did so by comparing the frequency of rare deleterious variants for cases and controls directly. We selected for genes harbouring deleterious variants more frequent in preeclampsia cases than in controls (variants in single genes in ≥10% of cases and ≤2% of controls). Five genes were identified; methylenetetrahydrofolate reductase (*MTHFR)*, inositol 1,4,5-trisphosphate receptor type 1 *(ITPR1)*, discs large MAGUK scaffold protein *2 (DLG2)*, sucrose isomaltase *(SI)*, and ataxin 1 (*ATXN1)* (Table 3).

**Table 3:** Rare predicted deleterious variants found in ≥10% of women with preeclampsia (cases) and 2% of controls.

Gene Symbol Transcript ID	Transcript Variant	Protein Variant	gnomAD§	CADD Score*	Clinvar/ HGMD (Variant Class) ¤	Variants present	
						Cases	Controls
*MTHFR* NM_005957.4	c.*3046G>A		0.372	<10	RCV000344422.1 (VUS)	1	0
	c.*2594C>T		0.206	<10	RCV000293884.1 (VUS)	1	0
	c.*1294G>A	p.A1430A	0.054	11.4	RCV000370067.1 (VUS)	1	0
	c.1970G>C	p.*657S	0.001	<10	CM035841 (Low activity)	1	0
	c.1409A>T	p.E470V	0.168	23.3	RCV000319501.1 (VUS)	1	1
	c.870C>T	p.N290N	0.020	10.1	RCV000310090.1 (VUS)	1	0
*ITPR1* NM_002222.5	c.-2A>G		0.041	15.2	RCV000399171.1 (VUS)	1	0
	c.195C>G	p.R65R	0.172	13.0	RCV000336374.1 (VUS)	1	0
	c.971C>A	p.A324D	0.003	23.4	-	1	0
	c.2985A>G	p.S995S	0.092	<10	-	1	0
	c.5076C>T	p.N1692N	0.188	15.7	RCV000278808.1 (VUS)	1	0
	c.6007C>T	p.H2003Y	-	29.6	RCV000177981.1 RCV000359508.1 (VUS)	1	0
	c.6321+1G>T		-	23.5	-	1	0
*DLG2* NM_001142699.1	c.*137C>T		-	22.3	-	2	0
	c.1799C>T	p.T600M	0.012	33.0	-	1	0
	c.837G>T	p.G279G	0.005	18.0	-	1	0
	c.357+188529C>T		0.375	15.6	-	1	0
	c.-16T>C		0.004	16.4	-	1	0
*SI* NM_001041.3	c.5279G>A	p.G1760D	0.017	22.7	-	1	0
	c.2923T>C	p.Y975H	0.409	26.6	RCV000366023.1 (VUS)	2	0
	c.1958G>T	p.G653V	-	33.0	-	1	0
	c.878G>A	p.G293D	0.000	31.0	-	1	0
*ATXN1* NM_000332.3	c.2114G>C	p.S705T	-	16.8	-	1	0
	c.772G>T	p.G258C	0.172	24.2	-	1	0
	c.642G>T	p.Q214H	0.368	11.6	-	1	0
	c.302C>T	p.T101M	0.019	24.5	-	1	0
	c.-748G>C		0.002	15.9	-	1	0

§) gnomAD frequency in %

*) CADD score above 20 belongs to the 1% most deleterious variants.

¤) Clinical significance reported in ClinVar.–Not present in database. VUS: variant of unknown significance.

### Presence of previously reported genes associated with preeclampsia

Finally, we investigated the presence of variants in four selected genes previously reported in relation to preeclampsia; the *ROCK2*, *ACVR2A*, *ERAP1*, and *ERAP2 genes* [17,19–21]. We chose to include rather frequent variants (<10%) to be able to assess potential accumulation of more frequent variants among cases. For *ROCK2*, we identified one variant among cases and one among controls. For *ACVR2A*, we identified one variant among cases and none among controls. We identified four *ERAP1* variants among cases and none among controls. For *ERAP2*, we identified 30 variants among cases and 18 among controls. When we searched for more rare variants (<1%), no difference was detected (Table 4).

**Table 4:** Predicted deleterious variants in ROCK2, AVCR2A, ERAP1, and ERAP2 in women with (cases) and without (controls) preeclampsia.

Gene Symbol Transcript ID	Transcript Variant	Protein Variant	gnomAD§	CADD score*	Clinvar/ HGMD (Variant Class) ¤	Variants present	
						Cases	Controls
*ROCK2* NM_004850.4	c.2833A>C	p.M945L	-	22.5	-	0	1
	c.407T>G	p.F136C	0.095	26.0	-	1	0
*ACVR2A* NM_001616.4	c.1077+36A>G	-	0.025	15.2	-	1	0
*ERAP1* NM_016442.4	c.2101-89C>T	-	0.184	15.6	-	1	0
	c.1939G>A	p.V647I	0.297	27.8	CM0911242 (DM?)	1	0
	c.1398G>A	p.Q466Q	0.009	15.6	-	1	0
	c.663+145G>A	-	0.055	15.9	-	1	0
*ERAP2* NM_022350.4	c.291C>T	p.I97I	7.912	18.3	-	12	8
	c.641C>T	p.P214L	1.824	32.0	-	2	0
	c.995T>C	p.V332A	0.010	<10	-	1	0
	c.1040C>T	p.T347M	2.149	26.8	-	5	2
	c.1232T>G	p.L411R	0.399	28.2	RCV000179970.1 (Benign)	0	1
	c.2006T>A	p.L669Q	3.937	26.9	CM0911418 (DP)	6	3
	c.2045A>T	p.D682V	0.020	24.0	-	0	1
	c.2726A>T	p.D909V	0.002	23.3	-	0	1
	c.2740-27G>A	-	0.375	16.4	-	4	1
	c.2855T>C	p.L952P	0.120	26.7	-	0	1

§) gnomAD frequency in %

*) CADD score above 20 belongs to the 1% most deleterious variants.

¤) Clinical Significance reported in ClinVar.–Not present in database. DM: disease-causing mutation, DP: disease-associated polymorphism.

## Discussion

We demonstrated a high degree of genetic concordance for cases and controls. Our findings therefore challenge the suggested hypothesis of a polygenetic aetiology for preeclampsia with a common network of susceptibility genes [13,20]. Our findings support previous twin studies that reported only minor genetic contribution for preeclampsia [22,23). The occurrence of preeclampsia in our cohort was lower (2.2%) than reported by others [6–9], but similar to previous reports on preeclampsia registration in the Danish Medical Birth Registry [26]. This may be explained by heterogeneity in the definition of preeclampsia used in different studies [13,16] and the fact that only preeclampsia requiring hospital admission are registered in the Danish Medical Birth Registry [30].

Cases did not appear more distinct from the reference genome than controls concerning variants in biological processes. Using the *disease association panel*, the controls carried a slightly higher frequency of variants in a higher number of genes compared to preeclampsia cases. This suggests that general variant accumulation, in the studied processes, does not contribute to the development of preeclampsia. Contrary, our finding that preeclampsia cases were less genetic diverse might be suggestive of a protective role of genetic diversity.

The genetic concordance was high for cases and controls for the signalling pathways. In the *gene panel* approach, cases carried slightly fewer variant genes per pathway than controls. The raw data from both the *disease association panel* and *gene panel* uncovered more variants in the von Willebrand factor gene, the polymerase DNA epsilon gene, and the *ITPR1* gene. Nevertheless, correction for multiple comparisons eliminated the statistical significant difference in variant numbers for any of the shared genes.

The overrepresentation of *ERAP2* variants in cases was explained by frequent variants. There was no difference when looking at the more rare variants (<1%). We found that deleterious variants in the *MTHFR*, *ITPR1*, *DLG2*, *SI*, and *ATXN1* genes were more frequent in cases compared to controls. The variants detected in *MTHFR* are reported in ClinVar as possibly associated with neural tube defects except p.*657S. p.*657S is reported in relation to MTHFR deficiency [44,45]. Variants in the *MTHFR* genes have, together with the most frequent inherited thrombophilia markers Factor V Leiden mutation and the prothrombin variant (G20210A), been reported associated with preeclampsia [17,46]. However, several reports have also largely refuted this association [14,46]. The *IPTR1* gene has been reported to be involved in maintenance of normal blood pressure through IP3R1-mediated regulation of eNOS [47]. The DLG2 gene is previously reported differentially expressed in transcripts of decidua basalis in preeclampsia [48]. Based on these findings, we cannot rule out a possible role of the *MTHFR*, *ITPR1*, and *DLG2* gene variants for risk of developing preeclampsia.

Variants in *SI* were reported in ClinVar as a variant of unknown significance. The *ATXN1* gene is a causative factor for spinocerebellar ataxia-1 [49] and has been suggested to participate in the highly conserved Notch signalling pathway with regulatory importance for embryonic development [50]. The variants in the *SI* and *ATXN1* genes demonstrated in the present study are of more dubious clinical relevance for preeclampsia.

### Presence of previously reported genes associated with preeclampsia

The *AVCR2*, *ROCK2*, *ERAP1*, and *ERAP2* genes previously reported associated with preeclampsia [19–21], reviewed in [14] were not found to be more frequent in cases compared to controls in the present study. Thus, our findings do not support previous findings of their possible contribution to risk of developing preeclampsia.

### Strengths and limitations

The major strength in the present study was the robust study design based on blood samples from an unselected cohort of pregnant women representative for the general Danish population of pregnant women. The Danish tax-paid health care system ensures free access for all inhabitants, including free antenatal care at midwives and general practitioners. Therefore, blood samples collected as a part of this antenatal care constituted a unique source for studying genetic variability according to risk of developing preeclampsia. The study was also strengthened by the fact that we based our case-identification on nationwide Danish registry data free from recall bias. The Danish Medical Birth Registry has complete coverage for all women giving birth in Denmark. The validity of the preeclampsia diagnosis is high in The Danish Medical Birth Registry, *i*.*e*., a positive and negative predictive value of 88% and 97% [26,37]. To further validate our case-selection, we obtained maternal demographic data and data on birth outcomes for both cases and controls. These data showed lower gestational ages and birth weights in the case group, consistent with the known clinical course in preeclampsia. Finally, we performed an in-depth sequencing and bioinformatics analysis of the entire exome of both cases and controls. This enabled direct comparison of variant frequencies in the two groups with reference to the reference genome [43].

We did not have access to data on family history of preeclampsia. Therefore, we cannot entirely rule out a family history of preeclampsia in the controls. The sample size may have limited the power of the present study, potentially leading to false-negative results [51]. However, we believe, that if any clinically relevant differences in the frequencies of protective or disease causing variants were present, it would have shown in the present study.

## Conclusion

In this explorative whole-exome sequencing study, we found no evidence of a common network of genetic variability predisposing to preeclampsia. Preeclampsia affects the reproductive success, wherefore it is biologically plausible that preeclampsia susceptibility genes are under negative evolutionary control, thereby keeping the population frequencies of susceptibility variant low [23]. The present study indicates that genetic markers carry a minor potential for predicting preeclampsia. Cases even had a reduced genetic variability compared to controls. Future studies should focus on the clinical impacts of this reduced variability in women suffering from preeclampsia.

## Supporting information

S1 Appendix (DOCX)
