# Supplementary material for: The genetic component of preeclampsia: A whole-exome sequencing study
Source: PLoS One. 2018 May 14;13(5):e0197217. doi: 10.1371/journal.pone.0197217 (PMC5951572; doi:10.1371/journal.pone.0197217)
Supplement: S1 Appendix — (DOCX) [file pone.0197217.s001.docx]

**S1 Appendix**

**S1 Supplementary methods**

**Whole exome sequencing and data filtering:**

Novogene Bioinformatics Technology Co., Hong Kong, performed the whole exome sequencing blinded to preeclampsia disease status. We used a total amount of 1.0 μg genomic DNA per sample as input material for the library preparation. Sequencing libraries were generated using Agilent SureSelect Human All Exon kit (Agilent Technologies, CA, USA) following manufacturer’s recommendations and index codes were added to each sample. In brief, fragmentation was carried out by hydrodynamic shearing system (Covaris, Massachusetts, USA) to generate 180-280 base pair fragments. Remaining overhangs were converted into blunt ends via exonuclease/polymerase activities and enzymes were removed. After adenylation of 3’ ends of DNA fragments, adapter oligonucleotides were ligated. DNA fragments with ligated adapter molecules on both ends were selectively enriched by PCR. Subsequently, libraries were hybridized with biotin labelled probes. Using magnetic beads with bound streptomycin, we captured 334,378 exons in 20,965 genes. Captured libraries were enriched by PCR to add index tags to prepare for hybridization. Products were purified using AMPure XP system (Beckman Coulter, Beverly, USA) and quantified using the Agilent high sensitivity DNA assay on the Agilent Bioanalyzer 2100 system. The clustering of the index-coded samples was performed on a cBot Cluster Generation System using TruSeq PE Cluster Kit v4-cBot-HS (Illumia, San Diego, USA) according to the manufacturer’s instructions. After cluster generation, the libraries were sequenced on an Illumina sequencing platform. Burrows-Wheeler Aligner (BWA, version 0.7.8-r455) was utilized to map the paired-end clean reads to the human reference genome (b37). After sorting with SAMtools (version 1.0) and marking duplicates with Picard (version 1.111), the results of read alignment were finally stored in BAM format. We then computed the coverage and depth based on the final BAM file. SNP and InDel detection was performed with GATK (Version 2015Mar22).

**S1 Table**

| **Filtering** | **Settings** |
| --- | --- |
| **Confidence** | Kept variants with call quality at least 20.0, with read depth at least 10.0, with allele fraction at least 25.0%, with genotype quality at least 30.0. |
| **Common variants** | We excluded variants that were observed with an allele frequency ≥ 0.5% of the genomes in the 1,000 genomes project, in NHLBI ESP exomes (All), in AFC, in ExAC, and in gnomAD |
| **Predicted deleterious** | We included variants that were:  Classified as pathogenic or likely pathogenic according to computed ACMG guidelines,  Disease-associated according to HGMD  Disease-associated according to CLINVAR  Frameshift, in-frame indel, or stop codon change  Missense unless predicted to be innocuous by SIFT or Polyphen-2  Predicted deleterious by having CADD score > 15.0  Disrupt splice site up to 2 bases into intron  Predicted to disrupt splicing by MaxEntScan. |
| **Disease Association Panel** | **Variants included in the following genes** |
| **Systemic lupus erythematosus**  471 affected genes | ABCB1, ACHE, ACTA1, ACTA2, ACTB, ACTC1, ACTG1, ACTG2, ACVR1, ACVR1B, ACVR1C, ACVR2A, ACVR2B, ADARB1, ADORA2A, AGFG1, AGRN, AKT1, ALG14, ALG2, ANXA1, ANXA5, APCS, APH1A, APH1B, APOE, AR, ATG5, AZU1, BANK1, BAX, BCHE, BCL2, BCL2L1, BCL2L11, BLK, BMPR2, BPI, BTNL2, C1QA, C1QB, C1QC, C1R, C1S, C2, C3, C3orf67, C4A/C4B, C5, C6orf10, C8A, CA3, CALM1, CAMP, CASP8, CCAR2, CCL3L1, CCND3, CD19, CD1A, CD1B, CD1C, CD1D, CD22, CD226, CD24, CD247, CD38, CD40, CD44, CD46, CD55, CD80, CD86, CDK2, CDKN1B, CEACAM1, CEACAM8, CFB, CFI, CHAT, CHRFAM7A, CHRM1, CHRM2, CHRM3, CHRM4, CHRM5, CHRNA1, CHRNA10, CHRNA2, CHRNA3, CHRNA4, CHRNA5, CHRNA6, CHRNA7, CHRNA9, CHRNB1, CHRNB2, CHRNB3, CHRNB4, CHRND, CHRNE, CHRNG, CIITA, CLEC16A, CLU, CNGB3, CNTF, COL13A1, COLQ, COX10, COX15, CR1, CR2, CREB1, CRISP3, CRP, CSF2, CSK, CSNK2B, CTLA4, CTSA, CTSG, CTSS, CXCL16, CXCL9, CYP2C19, DEFA4, DEFB1, DHFR, DKK3, DLG5, DNASE1, DNASE1L3, DNMT1, DOK7, DPAGT1, DYSF, E2F2, EGR2, EGR3, ELANE, ESR1, ESR2, ETS1, F2, F5, FAM167A-AS1, FAS, FASLG, FCAR, FCGR1A, FCGR1B, FCGR2A, FCGR2B, FCGR2C, FCGR3A/FCGR3B, FCGRT, FKBP1A, FLT1, FOXO1, GABRA1, GABRA2, GABRA3, GABRA4, GABRA5, GABRA6, GABRB1, GABRB2, GABRB3, GABRD, GABRE, GABRG1, GABRG2, GABRG3, GABRP, GABRQ, GABRR1, GABRR2, GABRR3, GATA3, GBP2, GFPT1, GMPPB, GNAQ, GNRH1, GRIN1, GRIN2A, GRIN2B, GRIN2C, GRIN2D, GRIN3A, GRIN3B, GRINA, GSTP1, GZMA, GZMB, H1F0, H1FX, H3F3A/H3F3B, HAVCR1, HCAR3, HCLS1, HFE, HIST1H1A, HIST1H1B, HIST1H1C, HIST1H1D, HIST1H1E, HIST1H1T, HIST1H3C, HIST1H4J, HIST2H3C, HIST3H3, HLA-DRA, HLA-DRB1, HLA-DRB3, HLA-DRB4, HLA-DRB5, HLA-G, HMGB1, HMGCR, HPRT1, HPS1, HRH1, HTR1A, ICA1, ICAM1, ICAM2, ICAM3, ICAM4, ICAM5, ICOS, IFIH1, IFIT1, IFNA1/IFNA13, IFNA10, IFNA14, IFNA16, IFNA17, IFNA2, IFNA21, IFNA4, IFNA5, IFNA6, IFNA7, IFNA8, IFNAR1, IFNAR2, IFNB1, IFNE, IFNG, IFNGR1, IFNK, IFNW1, IFRD1, IKZF1, IL10, IL12A, IL12B, IL16, IL17A, IL18, IL1B, IL1R2, IL1RN, IL2, IL2RB, IL4, IL6, IL6ST, IMPDH1, IMPDH2, IRF5, IRF7, IRF8, ISG15, ITGAM, ITGB7, ITPA, ITPR3, JAG1, KCP, KDR, KLRB1, KLRC4-KLRK1/KLRK1, LCK, LCN2, LILRA4, LRP4, LTF, LTK, LY6E, LY9, LYN, MAP3K8, MASP2, MBL2, MC2R, MECP2, MEFV, MFGE8, MICA, MMP8, MMP9, MPO, MS4A1, MSH5, MT2A, MTA2, MTHFR, MTOR, MUSK, MX1, MYO9A, NCF1, NCF2, NCR1, NCR3, NCSTN, NFKBIA, NFKBIL1, NMNAT2, NOD2, NOS3, NR3C1, OAS1, OAS2, OAS3, OASL, PDCD1, PDGFC, PEPD, PGF, PGR, PHRF1, PLEC, POLG, PPAT, PPP1CA, PPP1R8, PPP2CA, PPP3CA, PPP3CB, PPP3CC, PRKAR1A, PRKCA, PRKCD, PRL, PROK1, PSEN1, PSEN2, PSENEN, PTGS1, PTGS2, PTPN1, PTPN22, PTPN6, PTPRC, PXK, RAB27A, RAB31, RAB5A, RAG2, RAPSN, RASGRP3, RNASE2, RNASE3, RORC, RYR1, S1PR1, SCARF1, SCN4A, SCN5A, SCUBE1, SELE, SELL, SELP, SERPINC1, SERPING1, SGK1, SH2D1A, SIAE, SKIV2L, SLAMF6, SLAMF7, SLC15A4, SLC18A3, SLC5A7, SLC6A2, SLC6A4, SNAP25, SNTB2, SOCS1, SOD2, SOS2, SP1, SPP1, SRSF1, SRSF2, ST6GAL1, STAT1, STAT3, STAT4, STAT5A, STAT5B, STAT6, SURF1, SYT2, TACO1, TAP1, TBC1D10C, TBK1, TBX21, TCF7, TGFBR1, TGFBR2, TGFBR3, TIRAP, TLE3, TLR1, TLR10, TLR2, TLR3, TLR4, TLR5, TLR6, TLR7, TLR8, TLR9, TMEM39A, TNF, TNFAIP3, TNFRSF1A, TNFRSF1B, TNFRSF6B, TNFRSF9, TNFSF13, TNFSF13B, TNFSF4, TNIP1, TNK1, TNPO3, TNXB, TP53BP2, TRAF3IP2, TRAF5, TREX1, TRIM21, TRIM25, TUBB4A, TYK2, TYMS, UBASH3B, UBE2L3, UHRF1BP1, UNC13D, USP17L9P, VAMP1, VCAM1, VDR, VEGFA, VEGFB, VEGFC, VEGFD, WDFY4, XKR6, XRCC1, ZBP1, ZNF148, ZNF214 |
| **Abnormal immune tolerance**  7 affected genes | ADGRE1, CDKN1A, F8, IFNG, MAN2A1, PSMB11, RNF128 |
| **Chronic kidney disease**  820 affected genes | A1BG, ABCA1, ABCB1, ABCC2, ABCC8, ACAT2, ACE, ACHE, ACP5, ACTN4, ADCY8, ADIPOQ, ADK, ADM, ADORA1, ADORA2A, ADRB2, AGA, AGER, AGT, AGTR1, AGTR2, AGXT, AHI1, AHSG, AIF1, AKAP12, AKT1S1, ALB, ALDH1L1, ALDH7A1, ALG9, ALMS1, ALPL, AMBP, AMY2A, AMY2B, ANGPT1, ANLN, ANTXR1, ANXA5, APOA1, APOA2, APOA4, APOA5, APOB, APOC1, APOC2, APOC3, APOC4, APOD, APOE, APOF, APOH, APOL1, APOM, APRT, AR, ARHGAP24, ARHGDIA, ARL13B, ART1, ASB3/GPR75-ASB3, ASIC1, ASIC2, ASIC3, ASIC4, ASIC5, ATG5, ATP10B, ATP1A1, ATP1A2, ATP1A3, ATP1A4, ATP1B1, ATP1B2, ATP1B3, ATP2C1, ATP4A, ATP4B, ATP5I, ATP5J, ATP5O, ATRX, AVPR2, B9D1, B9D2, BAD, BAK1, BAX, BBS1, BBS10, BBS4, BBS9, BCL11A, BCL2, BCL2L1, BID, BMP1, BSND, BUB1B, BUD13, C12orf42, C1orf100, C2CD3, C3, C3AR1, C4A/C4B, C5, C5orf42, C9, CA1, CA12, CA13, CA14, CA2, CA3, CA4, CA5A, CA5B, CA6, CA7, CA9, CACNA1A, CACNA1B, CACNA1C, CACNA1D, CACNA1E, CACNA1F, CACNA1G, CACNA1H, CACNA1I, CACNA1S, CACNA2D1, CACNA2D2, CACNA2D3, CACNA2D4, CACNB1, CACNB2, CACNB3, CACNB4, CACNG1, CACNG2, CACNG3, CACNG4, CACNG5, CACNG6, CACNG7, CACNG8, CAMLG, CASR, CATSPER1, CATSPER2, CATSPER3, CATSPER4, CC2D2A, CCL2, CCL5, CCNA2, CCNB1, CCNC, CCND1, CCND2, CCND3, CCR2, CCR5, CD14, CD151, CD180, CD1D, CD2AP, CD46, CD80, CD86, CDH4, CDK2, CDKN1A, CDKN1B, CDV3, CELSR2, CEP104, CEP120, CEP164, CEP290, CEP41, CEP83, CETP, CFH, CFHR5, CFTR, CHUK, CKB, CKM, CLCN5, CLCNKB, CLDN16, CLDN19, CLEC2B, CLIC2, CLU, CNDP1, CNTN5, COG2, COL3A1, COL4A1, COL4A2, COL4A3, COL4A4, COL4A5, COL4A6, COMT, COPS8, COQ6, COQ8B, COX6C, COX7C, CPB2, CPOX, CRB2, CRP, CRYGD, CSF3, CSGALNACT1, CSPP1, CTGF, CTNNB1, CTNS, CX3CR1, CXCL12, CXCL8, CXCR2, CYBA, CYP1A1, CYP24A1, CYP2B6, CYP2C19, CYP2D6, CYP2R1, CYP3A4, CYP3A43, CYP3A5, CYP3A7, CYP3A7-CYP3A51P, DACT3, DBI, DCDC2, DCN, DDAH2, DGKE, DNTTIP2, DPP4, DRD1, DRD2, DRD3, DRD4, DRD5, EEF1B2, EGF, EGFEM1P, EGFR, EIF2AK4, ELMO1, ELN, EMC8, EPHA4, EPO, EPOR, EVC2, EXO1, EXOC3L2, EXOC4, EXOC8, EXOG, F10, F13A1, F2, F2R, F5, FABP1, FABP2, FABP3, FABP4, FABP5, FABP6, FABP7, FABP9, FAM111A, FAN1, FBLN1, FCGR1A, FCGR1B, FCGR2A, FCGR2B, FCGR2C, FCGR3A/FCGR3B, FDPS, FGA, FGB, FGF23, FGFR4, FKBP1A, FLT1, FN1, FRMD3, FTH1, FTL, FXYD2, GABRA1, GABRA2, GABRA3, GABRA4, GABRA5, GABRA6, GABRB1, GABRB2, GABRB3, GABRD, GABRE, GABRG1, GABRG2, GABRG3, GABRP, GBE1, GC, GDNF, GFPT2, GH1, GHR, GJA4, GLA, GLIS2, GLIS3, GLRA1, GNB1L, GNB3, GOLGA4, GPC5, GPNMB, GPR141, GPX1, GPX2, GPX3, GPX4, GPX5, GPX6, GPX7, GPX8, GRIA1, GSS, GSTA1, GSTK1, GSTM1, GSTM2, GSTP1, GSTT1, GSTT2/GSTT2B, GSTZ1, H3F3A/H3F3B, HACD4, HBA1/HBA2, HBEGF, HEXA, HEXB, HEXDC, HFE, HGF, HIST1H4J, HLA-G, HMGB1, HMGCR, HMGN3, HMOX1, HNF1A, HNF1B, HOXA13, HPR, HPRT1, HPSE, HPX, HSD11B2, HSP90AA1, HYLS1, ICAM1, IDO1, IFNA2, IFNAR1, IFNAR2, IFNG, IFT122, IFT140, IFT172, IFT43, IL10, IL13, IL18, IL1A, IL1B, IL1F10, IL1RN, IL2, IL2RA, IL2RB, IL33, IL36A, IL36B, IL36G, IL36RN, IL37, IL4, IL6, IMPDH1, IMPDH2, INF2, INPP5B, INPP5E, INSR, INVS, IQCB1, IRS2, ITGA2, ITGA3, ITGB3, ITIH4, ITPR1, ITPR2, ITPR3, JPH3, JUNB, KCNJ11, KCNN4, KCNQ1, KCP, KDM4B, KIAA0556, KIAA0586, KIAA0753, KIF14, KIF17, KIF7, KIT, KL, KLF14, KLF4, KLF5, KLK1, KLK10, KLK11, KLK12, KLK13, KLK14, KLK15, KLK2, KLK3, KLK4, KLK5, KLK6, KLK7, KLK8, KLK9, KLKB1, KLRA1P, KNG1, LAMB2, LCAT, LDLR, LGMN, LIN7C, LINC00923, LINC01241, LINC01441, LIPH, LMX1B, LPL, LRP1, LRRCC1, LSM3, LSM7, LTA, LTBP1, LZTFL1, MAGI2, MARCH10, MAS1, MBD2, MCEE, MEFV, MGST1, MGST2, MGST3, MICALL2, MKKS, MKS1, MLST8, MMAB, MMP1, MMP14, MMP2, MMP3, MR1, MRE11, MSC, MT-ATP6, MT-CO1, MT-RNR1, MT-RNR2, MT-TL1, MTHFR, MTOR, MTR, MTRR, MUC1, MUT, MVD, MYADML, MYC, MYH10, MYH9, MYO1E, MYO7B, NADSYN1, NAGLU, NAT8, NDUFA1, NDUFA6, NDUFB1, NDUFS5, NECTIN2, NEK8, NOD2, NOS1AP, NOS3, NPHP1, NPHP3, NPHP4, NPHS1, NPHS2, NPM1, NPPA, NPPB, NR3C1, NR3C2, NSA2, NUP107, NUP93, OCRL, OFD1, OPRD1, OPRK1, OPRM1, OR13G1, ORM1, ORM2, P2RY1, P2RY12, PARP1, PARP10, PARP11, PARP12, PARP14, PARP15, PARP16, PARP2, PARP3, PARP4, PARP6, PARP8, PARP9, PAX2, PAX8, PCNA, PCSK2, PCSK9, PCYOX1, PDE11A, PDE3A, PDE3B, PDE4A, PDE4B, PDE4C, PDE4D, PDE5A, PDE6D, PDE7A, PDE7B, PDE8A, PDGFD, PDPR, PECAM1, PFDN5, PHACTR3, PHB, PIBF1, PITRM1, PKD1, PKHD1, PKHD1L1, PLA2G4A, PLCE1, PLEKHM3, PLTP, PON1, PON2, PON3, PPARD, PPARG, PPAT, PPP3CA, PPP3CB, PPP3CC, PPP3R1, PPP3R2, PRDX6, PRKAR1B, PRKCB, PSMA1, PSMA2, PSMA3, PSMA4, PSMA5, PSMA6, PSMA7, PSMA8, PSMB1, PSMB10, PSMB11, PSMB2, PSMB3, PSMB4, PSMB5, PSMB6, PSMB7, PSMB8, PSMB9, PSMC1, PSMC2, PSMC3, PSMC4, PSMC5, PSMC6, PSMD1, PSMD10, PSMD11, PSMD12, PSMD13, PSMD14, PSMD2, PSMD3, PSMD4, PSMD5, PSMD6, PSMD7, PSMD8, PSMD9, PSME1, PSME2, PSME3, PSME4, PSMF1, PTAFR, PTGER1, PTGIR, PTGS1, PTGS2, PTH, PTHLH, PTPRO, PVT1, QKI, RAD51C, RAPGEF3, RAPGEF4, RBP4, RCOR1, REN, RNLS, RPGRIP1L, RPL23, RPL34, RPL36A, RPL7, RPTOR, RREB1, SAA1, SAA2, SAA4, SARS2, SCAP, SCGB1A1, SCN5A, SCNN1A, SCNN1B, SCNN1D, SCNN1G, SDCCAG8, SEMA3A, SERPINA1, SERPINC1, SERPINE1, SERPINF1, SERPINF2, SFTPC, SGCG, SKP2, SLC12A1, SLC12A3, SLC19A1, SLC2A9, SLC30A7, SLC34A1, SLC3A1, SLC6A2, SLC6A3, SLC6A4, SLC7A11, SLC7A7, SLC7A8, SLC9A1, SMAD3, SMARCAL1, SMURF2, SNRPE, SNRPG, SOAT1, SOAT2, SOD2, SOX18, SPP1, SPRY2, STC1, SYT7, TAF9, TAX1BP1, TCN2, TCTN1, TCTN2, TCTN3, TF, TFPI, TGFB1, TGFBR1, TGFBR2, THBD, TIPARP, TLR3, TLR4, TLR9, TMEM107, TMEM138, TMEM216, TMEM231, TMEM237, TMEM260, TMEM67, TMPO, TNF, TNFAIP3, TNFRSF11B, TNFRSF13B, TNFRSF1A, TNFRSF1B, TNFSF11, TNKS, TNKS2, TNNI3, TNNT1, TNNT2, TNNT3, TP53INP1, TPBG, TRA2B, TRAF3IP1, TRAK2, TRAPPC9, TRIM32, TRPC6, TRPV1, TTC21B, TTR, TUBA1A, TUBA1C, TUBA3C/TUBA3D, TUBA4A, TUBA8, TUBB1, TUBB2A, TUBB3, TUBB4A, TUBB4B, TUBD1, TUBE1, TUBG1, TUBG2, TXNDC15, UCP1, UCP2, UCP3, UMOD, UQCRB, UQCRH, VCAM1, VDR, VEGFA, VKORC1, VLDLR, VTN, WDPCP, WDR19, WDR35, WDR73, WFDC2, WRN, WT1, XDH, XPNPEP3, ZC3H15, ZC3HAV1, ZMIZ1, ZNF423, ZNF592, ZPR1 |
| **Coagulopathy (blood coagulation disorder)** 3,948 affected genes | A1BG, A2ML1, AARS, ABCA1, ABCA10, ABCA13, ABCA3, ABCA4, ABCA9, ABCB1, ABCB10, ABCB11, ABCB4, ABCB5, ABCB6, ABCB7, ABCB8, ABCC1, ABCC11, ABCC2, ABCC3, ABCC4, ABCC6, ABCC8, ABCC9, ABCD2, ABCD3, ABCD4, ABCG1, ABCG2, ABCG5, ABCG8, ABHD12B, ABHD17A, ABHD2, ABL1, ABL2, ABLIM1, ABO, ABTB1, ACAA1, ACAD10, ACAD11, ACAD9, ACAN, ACBD5, ACD, ACE, ACER2, ACHE, ACKR1, ACOT11, ACOT9, ACP2, ACP5, ACSL1, ACSL4, ACSL5, ACSM3, ACSM6, ACTA1, ACTA2, ACTB, ACTC1, ACTG1, ACTG2, ACTL6B, ACTL7B, ACTN1, ACTN2, ACTN4, ACTRT3, ACVR1, ACVR1B, ACVR1C, ACVR2A, ACVR2B, ADA, ADA2, ADAM22, ADAM28, ADAM7, ADAM9, ADAMTS12, ADAMTS13, ADAMTS16, ADAMTS5, ADAMTS9, ADAR, ADARB1, ADARB2, ADCY1, ADCY3, ADCY7, ADD2, ADGB, ADH1C, ADH4, ADH6, ADORA1, ADORA2A, ADORA3, ADRA1A, ADRA1B, ADRA1D, ADRA2A, ADRA2B, ADRA2C, ADRB1, ADRB2, ADRB3, AFTPH, AGAP6 , AGBL1, AGFG1, AGGF1, AGK, AGL, AGO1, AGT, AGTR2, AHI1, AHNAK, AHNAK2, AHSA1, AHSG, AHSP, AICDA, AIG1, AK7, AKAP12, AKAP13, AKAP17A, AKAP2, AKAP5, AKAP8, AKIRIN2, AKR1C3, AKR1C4, AKR1D1, AKT1, AKT1S1, AKT2, AKT3, ALAS2, ALB, ALDH1A1, ALDH2, ALDH4A1, ALDH5A1, ALG1, ALG10B, ALG11, ALG12, ALG13, ALG2, ALG3, ALG6, ALG8, ALG9, ALK, ALOX12, ALOX12B, ALOX15, ALPI, ALPK1, ALPK3, ALPL, ALPP, ALPPL2, AMACR, AMBP, AMER1, AMOT, AMPH, AMY1C , ANAPC5, ANG, ANGPT1, ANGPTL2, ANGPTL4, ANK2, ANK3, ANKFY1, ANKH, ANKIB1, ANKMY1, ANKRD11, ANKRD18A, ANKRD18B, ANKRD20A4 , ANKRD22, ANKRD26, ANKRD30A, ANKRD30B, ANKRD30BL, ANKRD36, ANKRD53, ANKRD62, ANKRD7, ANKS1B, ANLN, ANO4, ANO5, ANO6, ANO9, ANPEP, ANTXR1, ANXA1, ANXA11, ANXA2, ANXA4, ANXA5, ANXA6, ANXA7, AP1G2, AP2A2, AP3B1, AP3D1, APAF1, APBA1, APBA3, APC, APCDD1, APCS, APEX1, APH1A, APH1B, APLNR, APOA1, APOA2, APOA4, APOB, APOBEC1, APOBEC3A, APOBEC3D, APOC1, APOC2, APOC3, APOC4, APOD, APOE, APOF, APOH, APOL1, APOM, APP, AQP4, AQP7, AR, ARAF, ARG2, ARHGAP1, ARHGAP20, ARHGAP24, ARHGAP26, ARHGAP31, ARHGAP35, ARHGAP9, ARHGEF10, ARHGEF10L, ARHGEF5, ARID1A, ARID2, ARID3A, ARID4A, ARID4B, ARID5A, ARID5B, ARIH1, ARL11, ARL6IP5, ARL9, ARMS2, ARNT, ARNTL, ARRB1, ARRDC1, ARSA, ARSD, ASAH1, ASAP3, ASB15, ASCC1, ASH1L, ASMT, ASMTL, ASRGL1, ASS1, ASXL1, ASXL2, ASXL3, ATAD1, ATF5, ATF6B, ATF7IP, ATG4D, ATG5, ATG7, ATIC, ATL1, ATM, ATN1, ATOX1, ATP11A, ATP1A4, ATP1B1, ATP2A2, ATP2B2, ATP2B4, ATP2C1, ATP4A, ATP4B, ATP5A1, ATP6AP1, ATP6V0A2, ATP6V0A4, ATP6V0D2, ATP6V1A, ATP7B, ATP8A2, ATP8B1, ATP9B, ATR, ATRN, ATRNL1, ATRX, ATXN1, AURKA, AURKB, AVP, AVPR1A, AVPR1B, AVPR2, AXIN2, AXL, AZU1, B2M, B3GALNT2, B3GALT1, B3GAT1, B3GNT3, B4GALNT1, B4GALNT2, B4GALT1, B4GALT2, B4GAT1, BAALC, BAAT, BACH1, BACH2, BAG1, BAK1, BANK1, BAP1, BARD1, BAX, BBC3, BBS10, BBS12, BCAS1, BCAS3, BCAT1, BCL10, BCL11A, BCL11B, BCL2, BCL2L1, BCL2L11, BCL2L13, BCL2L14, BCL3, BCL6, BCLAF1, BCOR, BCORL1, BCR, BCS1L, BDNF, BDP1, BEND2, BEST1, BHLHE22, BICD2, BIRC5, BLACE, BLK, BLNK, BLOC1S3, BLOC1S6, BLVRA, BMP1, BMP4, BMPER, BMPR2, BMX, BORCS8, BPI, BRAF, BRCA1, BRCA2, BRD3, BRD7, BRD8, BRIP1, BSPH1, BTBD3, BTBD9, BTG1, BTG2, BTK, BTN2A1, BTNL2, BTNL9, BTRC, BUB1B, C10orf76, C10orf90, C14orf177, C16orf62, C17orf47, C19orf57, C1GALT1, C1GALT1C1, C1QA, C1QB, C1QC, C1R, C1S, C1orf158, C2, C20orf96, C2CD2, C2CD2L, C2orf27A/C2orf27B, C2orf54, C2orf71, C3, C3AR1, C3orf30, C3orf67, C4A/C4B, C5, C5orf15, C5orf42, C6, C6orf10, C6orf47, C8orf4, C8orf44-SGK3/SGK3, C9, C9orf84, CA10, CA2, CA3, CA9, CACNA1A, CACNA1B, CACNA1C, CACNA1D, CACNA1E, CACNA1F, CACNA1G, CACNA1H, CACNA1I, CACNA1S, CACNA2D1, CACNA2D2, CACNA2D3, CACNA2D4, CACNB1, CACNB2, CACNB3, CACNB4, CACNG1, CACNG2, CACNG3, CACNG4, CACNG5, CACNG6, CACNG7, CACNG8, CAD, CADM1, CALCRL, CALD1, CALM1 , CALML4, CALR, CAMK2N1, CAMKK1, CAMKK2, CAMLG, CAMP, CAMTA1, CAND2, CAPN12, CAPN2, CAPN5, CAPRIN2, CARD11, CARD14, CARD16, CASK, CASP10, CASP2, CASP7, CASP8, CASR, CAT, CATSPER1, CATSPER2, CATSPER3, CATSPER4, CATSPERE, CAV1, CBFA2T2, CBFB, CBL, CBLB, CBS/CBSL, CBWD1, CBWD2, CBX2, CCDC115, CCDC150, CCDC172, CCDC178, CCDC40, CCDC7, CCDC89, CCL17, CCL18, CCL2, CCL27, CCL3, CCL3L1, CCL3L3, CCL4, CCL5, CCL7, CCNA2, CCNB3, CCND1, CCND2, CCND3, CCNDBP1, CCNH, CCNYL1, CCPG1, CCR4, CCR5, CCR7, CD14, CD160, CD177, CD180, CD19, CD1A, CD1B, CD1C, CD1D, CD2, CD200, CD207, CD209, CD22, CD226, CD24, CD244, CD247, CD28, CD2AP, CD300E, CD33, CD34, CD36, CD38, CD3D, CD3E, CD3G, CD4, CD40, CD40LG, CD44, CD46, CD47, CD48, CD5, CD52, CD58, CD59, CD63, CD68, CD69, CD7, CD70, CD74, CD79A, CD79B, CD80, CD81, CD86, CD8A, CD8B, CD93, CD99, CDA, CDC20, CDC25A, CDC25C, CDC27, CDC42, CDC42EP2, CDC42EP3, CDC6, CDCA4, CDCP1, CDH10, CDK1, CDK11B, CDK12, CDK13, CDK2, CDK4, CDK5RAP2, CDK6, CDKN1A, CDKN1B, CDKN2A, CDKN2B, CDKN2C, CDKN2D, CDT1, CEACAM1, CEACAM20, CEACAM4, CEACAM8, CEBPA, CEBPB, CEBPE, CECR6, CELA3A, CELSR3, CENPI, CENPJ, CEP104, CEP112, CEP126, CEP164, CEP192, CEP57L1, CEP68, CEP89, CES1, CES2, CES3, CES4A, CES5A, CETP, CFAP161, CFB, CFH, CFHR1, CFHR3, CFI, CFL1, CFTR, CHAC2, CHAF1A, CHD1, CHD2, CHD4, CHD7, CHEK1, CHEK2, CHGA, CHGB, CHIA, CHIC2, CHID1, CHIT1, CHL1, CHPF, CHRM1, CHRM2, CHRM3, CHRM4, CHRM5, CHRNA1, CHRNA10, CHRNA2, CHRNA3, CHRNA4, CHRNA5, CHRNA6, CHRNA7, CHRNA9, CHRNB1, CHRNB2, CHRNB3, CHRNB4, CHRND, CHRNE, CHRNG, CHST15, CHSY1, CHSY3, CHUK, CIITA, CISD2, CISH, CKAP2L, CKB, CKM, CKMT1A/CKMT1B, CKMT2, CLCA1, CLCA4, CLCN2, CLCN7, CLCNKA, CLDN14, CLDN7, CLEC11A, CLEC16A, CLEC18A/CLEC18C, CLEC18B, CLEC1B, CLEC2B, CLEC4G, CLEC4M, CLEC7A, CLIC2, CLIC3, CLIP1, CLK1, CLSTN1, CLTB, CLTCL1, CLU, CNBD1, CNDP1, CNGA4, CNGB3, CNKSR1, CNOT1, CNOT3, CNOT4, CNTFR, CNTLN, CNTN3, CNTN5, CNTNAP1, CNTNAP2, CNTNAP3B, CNTNAP4, COCH, COG1, COG2, COG4, COG5, COG6, COG7, COG8, COL11A1, COL12A1, COL15A1, COL16A1, COL17A1, COL1A1, COL1A2, COL24A1, COL25A1, COL2A1, COL4A1, COL4A3, COL4A3BP, COL4A4, COL4A5, COL4A6, COL5A1, COL5A2, COL6A1, COL6A2, COL6A6, COL7A1, COMMD1, COMMD6, COMT, COQ6, COQ8B, CORO1A, CP, CPB2, CPD, CPNE1, CPS1, CPT1A, CPT1B, CPT1C, CPT2, CR1, CR2, CRBN, CRCP, CREB1, CREBBP, CRHR1, CRIP1, CRIPAK, CRIPT, CRISP3, CRLF2, CROCC, CRP, CRTAP, CRTC1, CRYGA, CRYGB, CSAG2/CSAG3, CSF1, CSF1R, CSF2, CSF2RA, CSF2RB, CSF3, CSF3R, CSK, CSMD1, CSNK1A1, CSNK2A1, CSNK2B, CSPG4, CT47A6 , CTAGE9 , CTBP1, CTBP2, CTC1, CTCF, CTLA4, CTNNB1, CTNND2, CTPS1, CTSA, CTSB, CTSG, CTSK, CTSL, CTSO, CTSW, CTTN, CUL1, CUL7, CUL9, CUX1, CUX2, CWH43, CX3CR1, CXCL1, CXCL10, CXCL12, CXCL13, CXCL16, CXCL8, CXCL9, CXCR1, CXCR4, CYCS, CYLC2, CYP1A1, CYP26A1, CYP27A1, CYP27B1, CYP2C19, CYP2C9, CYP2D6, CYP2E1, CYP2R1, CYP39A1, CYP3A4, CYP3A43, CYP3A5, CYP3A7, CYP3A7-CYP3A51P, CYP4F11, CYP51A1, CYP7A1, CYP7B1, DAAM1, DAAM2, DAB1, DAD1, DAG1, DAGLA, DCAF12, DCAF5, DCBLD2, DCC, DCHS2, DCK, DCLRE1A, DCLRE1C, DCN, DCST1, DCSTAMP, DCTD, DCTN2, DCTN3, DDC, DDOST, DDR1, DDR2, DDRGK1, DDX1, DDX11, DDX18, DDX3X, DDX4, DDX41, DDX46, DDX51, DDX58, DDX60, DEFA4, DEFB1, DEFB105A/DEFB105B, DENND3, DES, DGCR2, DGCR8, DGKB, DGKE, DGUOK, DHDDS, DHDH, DHFR, DHPS, DHRS12, DHRSX, DHTKD1, DHX16, DHX29, DHX32, DHX9, DIABLO, DIAPH1, DICER1, DICER1-AS1, DIO1, DIRC2, DISP1, DISP2, DKC1, DKK3, DLC1, DLEU7, DLG1, DLG2, DLG5, DLGAP3, DLL1, DLL4, DLX4, DMBT1, DMD, DMGDH, DMPK, DMXL1, DNAAF4, DNAH11, DNAH14, DNAH17, DNAH2, DNAH3, DNAH5, DNAH7, DNAH8, DNAH9, DNAI1, DNAJA1, DNAJB2, DNAJC21, DNAJC25, DNAJC5, DNASE1, DNASE1L3, DNASE2, DNER, DNHD1, DNM1L, DNM2, DNMT1, DNMT3A, DNMT3B, DNMT3L, DNTT, DOCK10, DOCK6, DOK1, DOK6, DOLK, DOPEY1, DOT1L, DPAGT1, DPCR1, DPEP1, DPM1, DPM2, DPM3, DPP4, DPYD, DPYD-AS1, DPYSL4, DRD2, DROSHA, DSC1, DST, DTHD1, DTNB, DTNBP1, DTX1, DTX3, DUSP10, DUSP21, DUSP4, DVL3, DYM, DYNC1H1, DYNC2H1, DYSF, DYTN, DZANK1, E2F1, E2F2, E2F3, E2F4, E2F8, E4F1, EBF1, EBF3, EBI3, ECEL1, ECHDC3, ECI2, EED, EEF1A1, EEF1D, EEFSEC, EFCAB5, EFCAB6, EFNA1, EFNA2, EFNA3, EFNA4, EFNA5, EFNB1, EFNB2, EFNB3, EGF, EGFLAM, EGFR, EGLN1, EGR1, EGR2, EGR3, EHBP1L1, EHMT1, EHMT2, EIF2AK1, EIF2S1, EIF3C, EIF3D, EIF3I, EIF4A1, EIF4G1, EIF4G2, EIF4G3, EIF4H, ELANE, ELAVL1, ELAVL2, ELAVL4, ELMO1, ELMOD3, ELOC, EML5, ENAM, ENC1, ENO2, ENPP1, ENTPD1, ENTPD2, EOGT, EOMES, EP300, EPAS1, EPB41L4B, EPCAM, EPG5, EPHA1, EPHA10, EPHA2, EPHA3, EPHA4, EPHA5, EPHA6, EPHA7, EPHA8, EPHB1, EPHB2, EPHB3, EPHB4, EPHB6, EPHX1, EPO, EPOR, ERBB2, ERBB3, ERBB4, ERC1, ERCC1, ERCC2, ERCC4, ERCC5, ERFE, ERG, ERICH3, ERO1B, ESCO2, ESD, ESPNL, ESR1, ESR2, ETFA, ETNK1, ETS1, ETV3, ETV5, ETV6, EVC, EXO1, EXOC4, EXOC5, EXOC6B, EYS, EZH2, F10, F11, F11R, F12, F13A1, F13B, F2, F2R, F3, F5, F7, F8, F8A1 , F9, FADD, FADS6, FAH, FAM104B, FAM120B, FAM129B, FAM160A2, FAM167A-AS1, FAM169B, FAM177A1, FAM184B, FAM19A5, FAM205A, FAM214B, FAM221B, FAM25A , FAM3B, FAM83E, FAM86B2/FAM86KP, FAM91A1, FANCA, FANCB, FANCC, FANCD2, FANCE, FANCF, FANCG, FANCI, FANCL, FANCM, FAR1, FARP1, FARS2, FAS, FASLG, FASN, FAT1, FAT2, FAT4, FBLN1, FBN1, FBXL2, FBXL7, FBXO38, FBXW10, FBXW7, FCAMR, FCAR, FCER1A, FCER1G, FCER2, FCGBP, FCGR1A, FCGR1B, FCGR2A, FCGR2B, FCGR2C, FCGR3A/FCGR3B, FCHSD1, FCN2, FDFT1, FDPS, FDXR, FEN1, FER, FERMT3, FETUB, FGA, FGB, FGD4, FGD6, FGF12, FGF2, FGF4, FGFR1, FGFR2, FGFR3, FGFR4, FGFRL1, FGG, FGL1, FGR, FHOD1, FHOD3, FITM2, FKBP10, FKBP1A, FKBP8, FKRP, FKTN, FLG, FLG2, FLI1, FLII, FLNA, FLNB, FLNC, FLOT2, FLT1, FLT3, FLT4, FMN2, FMNL3, FN1, FNDC7, FNIP2, FNTA, FNTB, FOLH1, FOLR1, FOS, FOXC1, FOXD4, FOXD4L1, FOXD4L3/FOXD4L6, FOXD4L4/FOXD4L5, FOXF1, FOXJ3, FOXM1, FOXN1, FOXO1, FOXO3, FOXP1, FOXP3, FPGS, FREM1, FRK, FRMPD1, FRS2, FRYL, FSD1, FUBP1, FUS, FUT10, FUT4, FUT7, FXN, FYB, FYN, G3BP1, G6PC, G6PC3, G6PD, GAA, GAB1, GAB2, GABBR2, GABRA1, GABRA2, GABRA3, GABRA4, GABRA5, GABRA6, GABRB1, GABRB2, GABRB3, GABRD, GABRE, GABRG1, GABRG2, GABRG3, GABRP, GABRQ, GABRR1, GABRR2, GABRR3, GAD2, GADD45A, GADD45G, GAGE1 , GAGE12F , GALC, GALE, GALK1, GALNT3, GALNT8, GALNT9, GALNTL6, GALT, GAMT, GAREM1, GART, GAS2, GAS7, GATA1, GATA2, GATA3, GATA4, GATA5, GBA, GBA2, GBA3, GBP2, GC, GCC2, GCKR, GCOM1, GDI1, GEMIN2, GEMIN4, GEN1, GFAP, GFI1B, GGCX, GGH, GGPS1, GGT1, GGT2/LOC102724197, GGT5, GGT6, GGT7, GGTLC1, GGTLC2, GH1, GHR, GIMAP1-GIMAP5, GIT1, GJB4, GJB5, GJC1, GJD2, GK, GLI1, GLIPR1, GLRB, GLRX5, GLS, GLT6D1, GLUD2, GMPPA, GMPPB, GNAQ, GNAS, GNAT1, GNB1, GNB1L, GNB3, GNE, GNG13, GNG5, GNPTAB, GNRH1, GOLGA1, GOLGA6A , GOLGA6L2, GOLGA8A/GOLGA8B, GOLGA8EP/GOLGA8G, GOLGA8F, GOLGA8K , GOSR2, GOT1, GOT1L1, GOT2, GP1BA, GP1BB, GP5, GP6, GP9, GPAT2, GPC4, GPER1, GPR101, GPR15, GPR156, GPR158, GPR174, GPRASP1, GPSM2, GPT, GPT2, GPX1, GPX2, GPX3, GPX4, GPX5, GPX6, GPX7, GPX8, GRB2, GREB1, GREM1, GRHL2, GRIN1, GRIN2A, GRIN2B, GRIN2C, GRIN2D, GRIN3A, GRIN3B, GRINA, GRIP1, GRK3, GRM1, GRM3, GRM6, GRN, GSE1, GSG2, GSK3B, GSN, GSTA1, GSTA2, GSTK1, GSTM1, GSTM2, GSTM3, GSTP1, GSTT1, GSTT2/GSTT2B, GSTZ1, GTF2B, GTF2I, GTPBP6, GTSE1, GUCY1A2, GUCY1A3, GUCY2C, GUK1, GULP1, GYG1, GYPA, GZMA, GZMB, GZMM, H1F0, H1FX, H2AFX, H3F3A/H3F3B, HABP2, HADH, HADHA, HADHB, HAMP, HAO2, HAPLN3, HAS1, HAT1, HAVCR1, HBA1/HBA2, HBB, HBD, HBE1, HBEGF, HBG1, HBG2, HBQ1, HBS1L, HBZ, HCAR3, HCFC1, HCK, HCLS1, HCST, HDAC1, HDAC10, HDAC11, HDAC2, HDAC3, HDAC4, HDAC5, HDAC6, HDAC7, HDAC8, HDAC9, HDC, HDHD5, HDLBP, HEATR1, HEATR4, HEATR6, HECTD1, HELB, HELZ, HERC1, HERC2, HERC2P3, HES1, HES5, HEY1, HEY2, HEYL, HFE, HFM1, HHLA2, HIC2, HIF1A, HIPK2, HIST1H1A, HIST1H1B, HIST1H1C, HIST1H1D, HIST1H1E, HIST1H1T, HIST1H2BO, HIST1H3C, HIST1H4J, HIST2H3C, HIST3H3, HIVEP1, HK3, HLA-A, HLA-B, HLA-C, HLA-DPA1, HLA-DQA1, HLA-DQB1, HLA-DQB1-AS1, HLA-DRA, HLA-DRB1, HLA-DRB3, HLA-DRB4, HLA-DRB5, HLA-G, HLCS, HLX, HMCN1, HMG20B, HMGB1, HMGCLL1, HMGCR, HMOX1, HMP19, HNF1A, HNF4G, HNRNPA2B1, HNRNPCL1/HNRNPCL2, HOMEZ, HOOK1, HOXA11, HOXA7, HOXA9, HOXB3, HOXB4, HOXB5, HOXD10, HOXD11, HOXD13, HOXD4, HPD, HPN, HPR, HPRT1, HPS1, HPS3, HPS4, HPS5, HPS6, HPX, HRAS, HRG, HRH1, HRNR, HS3ST6, HSD11B1, HSD3B1, HSD3B7, HSF1, HSF4, HSP90AA1, HSP90AB1, HSP90B1, HSPA5, HSPA6, HSPB1, HSPD1, HSPG2, HTR1A, HTR2A, HTR2B, HTR2C, HTR3D, HTRA2, HTT, HUWE1, HVCN1, HYAL1, HYAL2, HYDIN, HYPK, IARS, ICA1, ICAM1, ICAM2, ICAM3, ICAM4, ICAM5, ICK, ICOS, ID1, ID2, ID4, IDH1, IDH2, IDH3B, IDI2-AS1, IFI27, IFI44L, IFIH1, IFIT1, IFITM2, IFITM5, IFNA1/IFNA13, IFNA10, IFNA14, IFNA16, IFNA17, IFNA2, IFNA21, IFNA4, IFNA5, IFNA6, IFNA7, IFNA8, IFNAR1, IFNAR2, IFNB1, IFNE, IFNG, IFNGR1, IFNGR2, IFNK, IFNL3, IFNW1, IFRD1, IFT122, IFT43, IFT46, IFT80, IGF1R, IGF2, IGF2BP3, IGF2R, IGFBP1, IGFBP3, IGLL1/IGLL5, IGSF5, IGSF8, IKBKAP, IKBKB, IKBKG, IKZF1, IKZF2, IKZF3, IL10, IL11RA, IL12A, IL12B, IL12RB1, IL13, IL15, IL16, IL17A, IL17RA, IL18, IL18RAP, IL19, IL1A, IL1B, IL1F10, IL1R1, IL1R2, IL1RAP, IL1RAPL2, IL1RL1, IL1RL2, IL1RN, IL2, IL20RA, IL21, IL21R, IL23R, IL27, IL2RA, IL2RB, IL2RG, IL3, IL33, IL36A, IL36B, IL36G, IL36RN, IL37, IL3RA, IL4, IL4R, IL6, IL6R, IL6ST, IL7R, IL9, ILDR1, ILKAP, ILVBL, IMPA2, IMPDH1, IMPDH2, INCA1, INF2, INHA, INPP5A, INPP5D, INPPL1, INSC, INSR, INTU, IPO7, IQCA1, IRAK2, IRAK3, IRAK4, IREB2, IRF1, IRF2BP2, IRF4, IRF5, IRF6, IRF7, IRF8, IRS1, IRS2, IRS4, ISG15, ISPD, ITGA1, ITGA2, ITGA2B, ITGA3, ITGA4, ITGA5, ITGA6, ITGA9, ITGAE, ITGAL, ITGAM, ITGAX, ITGB1, ITGB1BP2, ITGB2, ITGB3, ITGB4, ITGB5, ITGB7, ITGBL1, ITIH4, ITK, ITPA, ITPR1, ITPR2, ITPR3, IVD, IZUMO3, JAG1, JAK1, JAK2, JAK3, JAKMIP2, JARID2, JDP2, JMJD6, JMJD7-PLA2G4B, JPH2, JPH3, JUN, KAT5, KAT6A, KAT7, KAZN, KCNA4, KCNA5, KCNC2, KCNE4, KCNG1, KCNH2, KCNH7, KCNJ1, KCNK13, KCNK18, KCNK4, KCNMB2, KCNMB3, KCNQ1, KCNQ2, KCNS2, KCP, KCTD19, KDM2B, KDM4B, KDM4E, KDM5A, KDM5C, KDM6A, KDR, KHDRBS1, KIAA0895L, KIAA1024, KIAA1147, KIAA1456, KIAA2022, KIF17, KIF1A, KIF1B, KIF1BP, KIF24, KIF25, KIF26A, KIF2B, KIF3A, KIF4B, KIRREL, KISS1R, KIT, KL, KLB, KLF1, KLF10, KLF17, KLF4, KLHDC1, KLHL13, KLHL32, KLHL4, KLHL8, KLK1, KLKB1, KLRB1, KLRC4, KLRC4-KLRK1/KLRK1, KMT2A, KMT2C, KMT2D, KMT2E, KMT5A, KNG1, KPNA1, KPNA2, KRAS, KRR1, KRT1, KRT10, KRT14, KRT18, KRT3, KRT36, KRT5, KRT6C, KRT73, KRT8, KRT81, KRT85, KRT86, KRTAP1-1, KRTAP10-10, KRTAP10-5, KRTAP10-6, KRTAP11-1, KRTAP19-1, KRTAP19-2, KRTAP19-3, KRTAP3-3, KRTAP4-12, KRTAP4-2, KRTAP4-7, KRTAP5-1, KRTAP5-3/KRTAP5-5, KSR1, KTI12, KY, L1CAM, LAMA1, LAMP1, LAMTOR2, LAPTM5, LARGE1, LARP1B, LARS, LARS2, LASP1, LAT, LAT2, LATS1, LAX1, LBP, LBR, LCAT, LCE4A, LCK, LCN2, LCT, LDB1, LDHB, LDLR, LDOC1, LEF1, LEKR1, LEP, LEPR, LEXM, LGALS1, LGALS2, LGALS4, LHCGR, LHX9, LIF, LIG1, LIG4, LILRA4, LILRB1, LILRB4, LILRB5, LIMS3/LIMS4, LINC00937, LINC01182, LINGO1, LIPA, LIPC, LIPT2, LLGL1, LMAN1, LMBR1, LMBRD1, LMBRD2, LMNA, LMNB1, LMO1, LMO2, LMX1B, LOC100287290, LOC101927851, LOC101929231, LOC102724788/PRODH, LOXL3, LPA, LPP, LRBA, LRFN2, LRGUK, LRP10, LRP1B, LRP2, LRP4, LRP5, LRP6, LRRC1, LRRC23, LRRC3, LRRC37A3 , LRRC40, LRRC49, LRRC53, LRRC7, LRRC8A, LRRCC1, LRRIQ4, LRRK1, LRRK2, LRRN2, LRRTM4, LTA, LTB, LTBP1, LTBP2, LTBP4, LTF, LTK, LUC7L2, LUC7L3, LUM, LUZP2, LY6E, LY9, LYL1, LYN, LYPLA1, LYPLA2, LYST, LYZ, LYZL2, LZTR1, MAATS1, MACC1, MAD1L1, MAD2L2, MADD, MAFG, MAGEA12, MAGEC1, MAGED4/MAGED4B, MAGI1, MAGI2, MAIP1, MAMDC2, MAML1, MAML2, MAML3, MAN1B1, MAN2B1, MAP1LC3B2, MAP2K1, MAP2K2, MAP2K4, MAP3K21, MAP3K8, MAP4K1, MAP4K2, MAP4K4, MAP4K5, MAP7, MAPK14, MAPK8IP1, MAPKAP1, MAPT, MARS, MARVELD3, MASTL, MAT1A, MAX, MBD4, MBL2, MBP, MBTPS2, MC2R, MCEE, MCFD2, MCL1, MCM6, MCM7, MDC1, MDM2, MDM4, MECOM, MECP2, MED1, MED12, MED13L, MED23, MED24, MED29, MEFV, MEGF10, MEI1, MEIOC, MEIS1, MELK, MET, MFAP1, MFAP2, MFGE8, MFN1, MGAT2, MGLL, MGMT, MGST1, MGST2, MGST3, MICA, MICAL3, MID2, MIF, MIOX, MIR4300HG, MITF, MKI67, MKL1, MKL2, MLF1, MLH1, MLLT10, MLLT3, MLPH, MLST8, MMAA, MMAB, MMACHC, MMADHC, MME, MMP1, MMP14, MMP16, MMP2, MMP7, MMP8, MMP9, MN1, MNT, MOAP1, MOCS1, MOGS, MON1B, MPDU1, MPDZ, MPI, MPIG6B, MPL, MPO, MPP1, MPP2, MPP3, MPP5, MPP6, MR1, MRAS, MRE11, MRPL17, MRPL37, MRPL47, MRPL51, MRPS5, MRS2, MS4A1, MS4A2, MSH2, MSH4, MSH5, MSH6, MSI2, MSR1, MSRB2, MST1, MSX2, MT-CO2, MT-CO3, MT-CYB, MT-ND4, MT2A, MTA2, MTCP1, MTHFD1, MTHFD1L, MTHFR, MTM1, MTMR10, MTMR11, MTMR4, MTO1, MTOR, MTPAP, MTR, MTRR, MTTP, MTUS1, MTX1, MUC12, MUC16, MUC17, MUC20, MUC4, MUC5AC, MUC5B, MUC6, MUT, MVK, MX1, MX2, MXI1, MXRA5, MYB, MYBL2, MYBPC2, MYC, MYCBP2, MYCN, MYD88, MYF5, MYH10, MYH11, MYH13, MYH2, MYH8, MYH9, MYL9, MYO15A, MYO18A, MYO1E, MYO5A, MYO7B, MYOCD, MYOD1, MYOG, MYOM3, MYOZ3, MYRIP, MZF1, N4BP3, NACAD, NAF1, NALCN, NAMPT, NANOS3, NAP1L3, NAPB, NASP, NAT1, NAT2, NBAS, NBEAL2, NBN, NBPF10 , NBPF3, NBPF4/NBPF6, NBR2, NCAM1, NCAN, NCBP2L, NCBP3, NCF1, NCF2, NCKAP5, NCOA3, NCOA6, NCOR1, NCOR2, NCR1, NCR3, NCSTN, NEB, NEDD4, NEDD9, NEIL1, NEK11, NEK3, NEK8, NETO1, NEUROD4, NEUROG3, NEXN, NF1, NFASC, NFAT5, NFE2, NFE2L2, NFIX, NFKB1, NFKB2, NFKBIA, NFKBIE, NFKBIL1, NGF, NGLY1, NHEJ1, NHLRC3, NHP2, NHSL1, NID1, NIF3L1, NINL, NIPBL, NIPSNAP3A, NIT1, NKTR, NKX2-1, NKX2-5, NKX2-6, NLGN1, NLGN4Y, NLRC3, NLRC4, NLRP12, NLRP2, NLRP3, NLRP9, NLRX1, NME1, NMNAT2, NMRAL1, NMT2, NMUR2, NOD1, NOD2, NOG, NOMO1 , NONO, NOP10, NOS1, NOS2, NOS3, NOSTRIN, NOTCH1, NOTCH2, NOTCH3, NOX4, NPAS3, NPAT, NPC1L1, NPEPPS, NPFFR1, NPHS1, NPHS2, NPIPA7 , NPM1, NPM2, NPPB, NPR2, NPTXR, NQO1, NQO2, NR0B2, NR1H3, NR1H4, NR1I3, NR3C1, NR4A1, NR5A1, NRAP, NRARP, NRAS, NRCAM, NRK, NRP1, NRP2, NSD1, NSD2, NSD3, NSDHL, NSMAF, NSUN2, NSUN7, NT5C2, NT5C3A, NTRK1, NTRK2, NTRK3, NUAK2, NUFIP1, NUGGC, NUMA1, NUMB, NUP107, NUP153, NUP188, NUP214, NUP98, NUS1, NUTF2, NUTM2A/NUTM2B, NXF3, NXPE1, NYNRIN, OAS1, OAS2, OAS3, OASL, OBSCN, OC90, OCLN, ODF3, ODF4, OGG1, OLIG2, OPA1, OPN4, OR10A7, OR10G2, OR10G3, OR10G7, OR10G9, OR11H12 , OR11H4, OR12D2, OR13C2, OR1L3, OR1N1, OR2A1/OR2A42, OR2A12, OR2A4/OR2A7, OR2B11, OR2C1, OR2C3, OR2F2, OR2S2, OR2T12, OR2T29/OR2T5, OR2V2, OR2W3, OR4F17 , OR4F21 , OR4F6, OR4K13, OR4K2, OR4K5, OR4N5, OR51B4, OR51G1, OR51Q1, OR51T1, OR52A1, OR52A5, OR52D1, OR52E8, OR52M1, OR52N2, OR52W1, OR5H1, OR5H6, OR5K4, OR5L1, OR5T3, OR6C4, OR6C74, OR6K3, OR7A17, OR8K1, OR8K3, OR9G1, ORC5, ORM1, ORM2, OSBPL10, OSBPL1A, OSTM1, OTOA, OTOP2, OTOR, OTUB1, OXER1, P2RX1, P2RY1, P2RY12, P2RY6, P3H1, PADI1, PADI2, PAH, PAK1, PAK2, PAK5, PALB2, PALD1, PALM2-AKAP2, PAMR1, PAN3, PANK4, PAPOLG, PAQR6, PAQR9, PARD3, PARN, PARP1, PARP4, PAX1, PAX2, PAX5, PAXIP1, PBX3, PCCA, PCCB, PCDH10, PCDH11X, PCDH15, PCDH7, PCDHA1, PCDHA10, PCDHA11, PCDHA12, PCDHA13, PCDHA2, PCDHA3, PCDHA4, PCDHA5, PCDHA6, PCDHA7, PCDHA8, PCDHA9, PCDHAC1, PCDHAC2, PCDHB1, PCDHB10, PCDHB12, PCDHB13, PCDHB16, PCDHB4, PCDHB5, PCDHGA1, PCDHGB2, PCDHGB7, PCF11, PCMTD1, PCNA, PCNX2, PCSK5, PCSK9, PCYOX1, PDCD1, PDCD5, PDE11A, PDE1B, PDE1C, PDE3A, PDE3B, PDE4A, PDE4B, PDE4C, PDE4D, PDE4DIP, PDE5A, PDE7A, PDE7B, PDE8A, PDGFA, PDGFB, PDGFC, PDGFRA, PDGFRB, PDHA1, PDIA2, PDP1, PDPK1, PDS5B, PDSS2, PEBP4, PEG3, PENK, PEPD, PER3, PEX1, PEX10, PEX11B, PEX12, PEX13, PEX14, PEX16, PEX19, PEX2, PEX26, PEX3, PEX5, PEX6, PF4, PFDN1, PFKFB3, PGA4 , PGBD2, PGF, PGK2, PGM1, PGM3, PGR, PHACTR1, PHEX, PHF1, PHF24, PHF3, PHF6, PHGDH, PHIP, PHKB, PHLDA3, PHRF1, PHYHD1, PHYHIPL, PI4KB, PIAS1, PIAS2, PICALM, PIGA, PIGO, PIGT, PIK3C2A, PIK3C2B, PIK3C2G, PIK3C3, PIK3CA, PIK3CB, PIK3CD, PIK3CG, PIK3R1, PIK3R2, PIK3R3, PIK3R4, PIK3R5, PIK3R6, PINK1, PIP4K2A, PITRM1, PKD1L1, PKD1L2, PKD2, PKHD1, PKP2, PKP3, PLA2G4A, PLA2G4F, PLA2G6, PLA2G7, PLA2R1, PLAGL2, PLAT, PLAU, PLCE1, PLCG1, PLCG2, PLCL1, PLCL2, PLCXD1, PLD2, PLEK, PLEKHA1, PLEKHG6, PLEKHG7, PLEKHM1, PLEKHM2, PLG, PLIN4, PLIN5, PLK1, PLOD2, PLP1, PLPPR1, PLTP, PLXDC2, PLXNA4, PLXNB1, PLXNC1, PMEL, PMF1/PMF1-BGLAP, PML, PMM2, PMS2, PNKD, PNP, PNPLA3, PNPT1, POLA1, POLB, POLD1, POLDIP3, POLE, POLE2, POLE3, POLE4, POLG, POLQ, POLRMT, POMGNT1, POMGNT2, POMK, POMT1, POMT2, PON1, PON3, POR, POT1, POTEB/POTEB2, POTEC, POTEE/POTEF, POTEG , POTEI, PPARA, PPARG, PPARGC1A, PPAT, PPBP, PPFIA2, PPIAL4G , PPIB, PPIP5K1, PPP1CA, PPP1CB, PPP1R12B, PPP1R13L, PPP1R14B, PPP1R1B, PPP1R2, PPP1R3B, PPP1R8, PPP2CA, PPP2R1B, PPP2R2A, PPP2R2B, PPP2R3B, PPP3CA, PPP3CB, PPP3CC, PPP3R1, PPP3R2, PPP4R1, PPP4R4, PPP6R3, PPTC7, PRAMEF11, PRAMEF14, PRAMEF18/PRAMEF19, PRAMEF20, PRAMEF4 , PRAMEF7/PRAMEF8, PRDM1, PRDM15, PRDM2, PRDM9, PRDX3, PRDX4, PRDX6, PREX2, PRF1, PRG4, PRICKLE2, PRIM1, PRIM2, PRKAA2, PRKACG, PRKAR1A, PRKCA, PRKCB, PRKCD, PRKCE, PRKCG, PRKCH, PRKCI, PRKCQ, PRKCZ, PRKD1, PRKD3, PRKDC, PRL, PRMT5, PRNP, PROC, PROCR, PROK1, PROM1, PROS1, PROZ, PRPF40B, PRPF8, PRPS1, PRR14L, PRR20A , PRR35, PRR5, PRRC2A, PRSS1, PRSS12, PRSS2, PRSS21, PRSS22, PRSS3, PRSS33, PRSS48, PRTN3, PRX, PSAP, PSD, PSEN1, PSEN2, PSENEN, PSIP1, PSMA1, PSMA2, PSMA3, PSMA4, PSMA5, PSMA6, PSMA7, PSMA8, PSMB1, PSMB10, PSMB11, PSMB2, PSMB3, PSMB4, PSMB5, PSMB6, PSMB7, PSMB8, PSMB9, PSMC1, PSMC2, PSMC3, PSMC3IP, PSMC4, PSMC5, PSMC6, PSMD1, PSMD10, PSMD11, PSMD12, PSMD13, PSMD14, PSMD2, PSMD3, PSMD4, PSMD5, PSMD6, PSMD7, PSMD8, PSMD9, PSME1, PSME2, PSME3, PSME4, PSMF1, PSRC1, PTAFR, PTCH2, PTEN, PTGER1, PTGES3L, PTGES3L-AARSD1, PTGFRN, PTGIR, PTGIS, PTGS1, PTGS2, PTH, PTH1R, PTK2, PTK2B, PTPMT1, PTPN1, PTPN11, PTPN13, PTPN2, PTPN21, PTPN22, PTPN6, PTPRA, PTPRC, PTPRD, PTPRE, PTPRF, PTPRG, PTPRK, PTPRN2, PTPRO, PTPRT, PTPRZ1, PTTG1, PUS7L, PVRIG, PXDN, PXK, PYGL, PYGM, QRICH2, R3HDM1, RAB24, RAB27A, RAB31, RAB40A, RAB4A, RAB5A, RAB6C/WTH3DI, RAB7A, RABGEF1, RABL2B, RAC1, RAC2, RACK1, RAD21, RAD23A, RAD50, RAD51, RAD51C, RAD9A, RAF1, RAG1, RAG2, RALA, RANBP17, RANBP3, RAPGEF1, RAPGEF6, RARA, RARB, RARG, RARS2, RASA1, RASA2, RASA4, RASA4B, RASAL2, RASGEF1C, RASGRP1, RASGRP2, RASGRP3, RASGRP4, RASSF2, RASSF4, RB1, RBBP4, RBL2, RBM12B, RBM15, RBM19, RBM41, RBM42, RBM7, RBM8A, RBMX, RBP4, RBPJ, RBPMS2, RCBTB1, RCC1, RDH10, RDH16, RECQL4, REL, RELA, RELB, RELN, RET, REV1, REV3L, RFC1, RFT1, RGL4, RGPD4 , RGS10, RGS18, RHOA, RHOB, RHOBTB1, RHOF, RHOH, RHOU, RHOV, RHOXF1, RHOXF2/RHOXF2B, RHPN2, RICTOR, RIF1, RIMBP2, RIMBP3 , RIPK3, RIT1, RIT2, RLF, RMRP, RNASE2, RNASE3, RNASEH2A, RNASEH2C, RND2, RND3, RNF111, RNF123, RNF13, RNF146, RNF168, RNF169, RNF17, RNF212, RNF214, RNPEP, RNU12, ROCK2, ROR1, ROR2, RORB, RORC, RP1L1, RPE65, RPL10, RPL11, RPL18, RPL3, RPL32, RPL34, RPL37, RPL3L, RPL41, RPL5, RPL6, RPN2, RPS11, RPS12, RPS13, RPS16, RPS19, RPS23, RPS24, RPS25, RPS3A, RPS4X, RPS6, RPS6KC1, RPTOR, RPUSD1, RRAS, RRAS2, RRBP1, RREB1, RRM1, RRM2, RRM2B, RRN3, RSPH10B/RSPH10B2, RSU1, RTEL1, RTL1, RUNDC1, RUNDC3B, RUNX1, RUNX1T1, RUNX2, RUNX3, RUSC2, RUVBL2, RWDD2B, RWDD3, RXRA, RXRB, RXRG, RYR1, RYR2, RYR3, S100A9, S100PBP, S1PR1, S1PR2, S1PR3, SAA1, SAA2, SAA4, SAC3D1, SALL2, SALL3, SALL4, SAMD8, SAMD9L, SAMHD1, SAP30, SAPCD2, SARS, SARS2, SASH3, SATB1, SBDS, SBF2, SBNO2, SC5D, SCARB1, SCARB2, SCARF1, SCD, SCN10A, SCN2A, SCN5A, SCN9A, SCP2, SCRT2, SCUBE1, SCYL2, SDCBP, SDHD, SEC23B, SEC24D, SELE, SELENBP1, SELL, SELP, SEMA3B, SEMA3D, SEMA4C, SEMA5A, SEMA5B, SEMA6A, SEPT6, SEPT8, SERAC1, SERF2, SERINC3, SERPINA1, SERPINA13P, SERPINB2, SERPINB3, SERPINB5, SERPINB8, SERPINC1, SERPIND1, SERPINE1, SERPINF1, SERPINF2, SERPINH1, SERTAD1, SESN2, SESN3, SETBP1, SETD1B, SETD2, SETD9, SETDB2, SETX, SEZ6L, SF3A1, SF3B1, SF3B2, SGCZ, SGK1, SGK2, SGPL1, SGPP2, SGSH, SH2B1, SH2B2, SH2B3, SH2D1A, SH2D2A, SH3BP1, SH3BP2, SH3GL1, SH3KBP1, SH3TC1, SH3YL1, SHANK1, SHANK2, SHCBP1L, SHMT1, SHOC2, SHROOM4, SI, SIAE, SIGLEC11, SIGLEC15, SIGLEC16, SIGMAR1, SIN3A, SIPA1, SIPA1L1, SIPA1L2, SIRPA, SIRPB1, SIRPG, SIRT1, SKAP1, SKI, SKIV2L, SKP2, SLAMF6, SLAMF7, SLC10A1, SLC10A2, SLC11A1, SLC12A2, SLC13A2, SLC14A1, SLC14A2, SLC15A4, SLC16A7, SLC17A1, SLC18A1, SLC19A1, SLC19A2, SLC19A3, SLC22A1, SLC22A14, SLC24A2, SLC24A4, SLC25A13, SLC25A32, SLC25A39, SLC25A5, SLC25A6, SLC27A2, SLC27A4, SLC27A6, SLC28A1, SLC29A1, SLC29A3, SLC2A2, SLC34A2, SLC35A1, SLC35A2, SLC35B1, SLC35B4, SLC35C1, SLC37A4, SLC38A1, SLC39A8, SLC40A1, SLC44A2, SLC45A4, SLC46A1, SLC4A1, SLC4A2, SLC5A5, SLC5A9, SLC6A15, SLC6A2, SLC6A3, SLC6A4, SLC6A6, SLC6A8, SLC7A2, SLC7A7, SLC7A8, SLC9A1, SLC9A4, SLCO1B1, SLCO1B3, SLCO1B7, SLCO1C1, SLCO3A1, SLCO6A1, SLFN14, SLITRK2, SLX4, SMAD4, SMAD6, SMAD9, SMARCA2, SMARCA4, SMARCAL1, SMARCB1, SMARCC2, SMC1A, SMC3, SMC5, SMG6, SMO, SMOX, SMPD1, SMPD3, SMTN, SMU1, SMYD2, SMYD3, SNAI2, SNCA, SNCAIP, SND1, SNRK, SNRNP70, SNRPE, SNX1, SNX10, SNX16, SOAT1, SOAT2, SOCS1, SOCS3, SOD1, SOD2, SOD3, SORBS2, SORCS2, SORCS3, SOS1, SOS2, SOST, SOX30, SOX4, SOX6, SP1, SP140, SP3, SP7, SPAG9, SPANXN1, SPARC, SPATA24, SPATA31A1 , SPATA5, SPATA6L, SPDYE2 , SPEN, SPHK1, SPHK2, SPI1, SPP1, SPRED1, SPRY1, SPSB3, SPTBN4, SRC, SRCAP, SRD5A3, SREBF1, SREK1, SRF, SRGAP3, SRMS, SRP54, SRPK2, SRPK3, SRRM1, SRRM2, SRRM4, SRSF1, SRSF2, SSB, SSBP2, SSR4, SSTR1, SSTR2, SSTR3, SSTR4, SSTR5, SSX3, ST3GAL4, ST3GAL6, ST6GAL1, ST6GALNAC3, ST6GALNAC4, ST7, ST8SIA3, ST8SIA6, STAB2, STAG1, STAG2, STAG3, STAT1, STAT3, STAT4, STAT5A, STAT5B, STAT6, STEAP4, STIM1, STIP1, STK11, STK3, STK32B, STK32C, STK38L, STK39, STK40, STMN2, STON2, STOX2, STRADB, STRN, STRN3, STS, STT3A, STT3B, STX11, STXBP2, SUCLA2, SUCLG1, SUFU, SUGP2, SULT1A1, SULT1A2, SULT1A3/SULT1A4, SUPT5H, SURF6, SUSD2, SUV39H2, SUZ12, SV2A, SVEP1, SWAP70, SYCP2, SYDE2, SYK, SYMPK, SYN1, SYN3, SYNE1, SYNPO2, SYTL5, TACC2, TACR3, TADA3, TAF1, TAF10, TAF13, TAF15, TAF1L, TAF6, TAF8, TAL1, TAL2, TALDO1, TAOK2, TAOK3, TAP1, TAP2, TARP, TAS1R2, TATDN2, TAX1BP1, TBC1D10C, TBC1D2, TBC1D3P2, TBC1D8B, TBC1D9, TBK1, TBL1XR1, TBP, TBX1, TBX21, TBXA2R, TBXAS1, TCEA3, TCEAL3, TCEB3C , TCF12, TCF3, TCF4, TCF7, TCIRG1, TCL1A, TCN2, TDP2, TDRD10, TEC, TEK, TEKT5, TELO2, TENM2, TEP1, TERC, TERF1, TERT, TET1, TET2, TEX15, TF, TFDP1, TFE3, TFPI, TFR2, TFRC, TGFB1, TGFB2, TGFB3, TGFBR1, TGFBR2, TGFBR3, TGM3, TGM6, TGOLN2, THAP11, THBD, THBS1, THBS2, THEM4, THOC2, THPO, THRAP3, THSD7B, TIA1, TIAF1, TIGAR, TIGIT, TIMD4, TIMM23B, TINF2, TIRAP, TJP1, TJP2, TLE3, TLL1, TLR1, TLR10, TLR2, TLR3, TLR4, TLR5, TLR6, TLR7, TLR8, TLR9, TLX1, TLX3, TMC1, TMC4, TMCO3, TMCO4, TMED7, TMEM132A, TMEM132B, TMEM132C, TMEM158, TMEM165, TMEM168, TMEM179, TMEM199, TMEM2, TMEM220, TMEM236, TMEM255B, TMEM259, TMEM268, TMEM38B, TMEM39A, TMEM45B, TMEM5, TMEM50B, TMEM63B, TMEM70, TMEM89, TMPRSS11B, TMPRSS9, TMSB10/TMSB4X, TMTC2, TNC, TNF, TNFAIP3, TNFAIP6, TNFRSF11A, TNFRSF11B, TNFRSF13B, TNFRSF13C, TNFRSF1A, TNFRSF1B, TNFRSF6B, TNFRSF8, TNFRSF9, TNFSF10, TNFSF11, TNFSF12, TNFSF12-TNFSF13, TNFSF13, TNFSF13B, TNFSF14, TNFSF15, TNFSF18, TNFSF4, TNFSF8, TNFSF9, TNIK, TNIP1, TNK1, TNK2, TNNC1, TNNC2, TNPO3, TNRC6A, TNRC6B, TNS1, TNXB, TOB2, TOP1, TOP2A, TOP2B, TOX, TOX4, TP53, TP53BP1, TP53BP2, TP53TG3D , TP63, TP73, TPM2, TPM4, TPMT, TPP2, TPSAB1/TPSB2, TPSD1, TPSG1, TRADD, TRAF3, TRAF3IP2, TRAF5, TRAF6, TRAM2, TRAPPC11, TRAPPC9, TRDMT1, TREML1, TRERF1, TREX1, TRHDE, TRIB1, TRIM10, TRIM11, TRIM21, TRIM24, TRIM25, TRIM3, TRIM42, TRIM6, TRIM6-TRIM34, TRIML1, TRIO, TRIP11, TRMU, TRNT1, TRPM3, TRPM6, TRPM8, TRRAP, TSC2, TSHZ2, TSHZ3, TSPAN15, TSPAN3, TSPAN33, TSPAN8, TSPO, TSPO2, TSR1, TSSK1B, TSSK3, TSSK6, TTC19, TTC21B, TTC26, TTF2, TTL, TTLL10, TTLL3, TTN, TTR, TUBA1A, TUBA1B, TUBA1C, TUBA3C/TUBA3D, TUBA3E, TUBA4A, TUBA4B, TUBA8, TUBB, TUBB1, TUBB2A, TUBB2B, TUBB3, TUBB4A, TUBB4B, TUBB6, TUBB8, TUBD1, TUBE1, TUBG1, TUBG2, TULP4, TUSC3, TWNK, TWSG1, TXK, TXNRD1, TXNRD3, TYK2, TYMS, TYRO3, TYROBP, U2AF1/U2AF1L5, U2AF2, UBA6, UBAP1, UBASH3B, UBB, UBBP4, UBE2C, UBE2D1, UBE2I, UBE2J1, UBE2J2, UBE2L3, UBE2NL, UBE2T, UBE3C, UBR3, UBTD2, UBXN1, UBXN11, UBXN4, UCKL1, UEVLD, UGCG, UGP2, UGT1A1, UGT1A3, UGT1A5, UGT1A6, UGT1A7 , UGT2A1, UGT2B10, UGT2B15, UGT2B28, UGT2B7, UGT3A2, UHRF1BP1, ULK3, ULK4, UMODL1, UNC13A, UNC13B, UNC13D, UNC93B1, UNG, UNK, UPB1, UPF2, UQCRB, UQCRC1, UQCRFS1, UQCRQ, UROC1, UROS, USB1, USH1G, USP15, USP16, USP17L2 , USP17L24 , USP17L9P, USP18, USP26, USP3, USP30, USP32, USP44, USP6NL, USP9X, UVSSA, VAMP7, VASH1, VAV1, VCAM1, VCAN, VCX/VCX3A, VDR, VEGFA, VEGFB, VEGFC, VEGFD, VHL, VIPAS39, VKORC1, VMP1, VNN1, VPS13A, VPS13D, VPS16, VPS33B, VPS41, VPS45, VPS4A, VPS4B, VPS54, VSIG1, VSTM4, VTI1A, VTN, VWA3B, VWF, WAC, WAS, WASH3P, WASHC2A/WASHC2C, WASHC4, WDFY4, WDR12, WDR25, WDR33, WDR38, WDR62, WDR78, WDR91, WEE1, WEE2, WFDC3, WFS1, WIPF1, WLS, WNT1, WRAP53, WRN, WT1, WT1-AS, WWOX, XBP1, XCL1, XDH, XIAP, XIRP1, XIRP2, XKR4, XKR6, XPC, XPNPEP2, XPO1, XPO5, XPO7, XPOT, XRCC1, XRCC2, XRCC3, XRCC4, XRCC5, XRCC6, XYLT1, XYLT2, YBX2, YES1, YLPM1, YWHAE, ZAN, ZAP70, ZAR1L, ZBBX, ZBED1, ZBED6CL, ZBP1, ZBTB16, ZBTB33, ZBTB39, ZBTB44, ZBTB7A, ZBTB7B, ZBTB8B, ZC3H12C, ZC3H13, ZC3H18, ZC3H8, ZDBF2, ZDHHC19, ZDHHC22, ZEB2, ZER1, ZFHX4, ZFP2, ZFP36L1, ZFP36L2, ZFP37, ZFP64, ZFPL1, ZFPM1, ZFYVE26, ZGRF1, ZHX2, ZIM2, ZKSCAN3, ZMIZ1, ZMIZ2, ZMYM1, ZNF10, ZNF107, ZNF148, ZNF169, ZNF180, ZNF185, ZNF207, ZNF208, ZNF212, ZNF214, ZNF215, ZNF229, ZNF230, ZNF276, ZNF277, ZNF347, ZNF385A, ZNF385D, ZNF395, ZNF423, ZNF440, ZNF449, ZNF470, ZNF484, ZNF492/ZNF98, ZNF512B, ZNF526, ZNF527, ZNF540, ZNF543, ZNF549, ZNF551, ZNF552, ZNF554, ZNF572, ZNF595, ZNF600, ZNF609, ZNF613, ZNF614, ZNF621, ZNF652, ZNF670, ZNF671, ZNF679, ZNF683, ZNF705B/ZNF705D, ZNF711, ZNF718, ZNF74, ZNF746, ZNF749, ZNF804B, ZNF827, ZNF83, ZNF835, ZNF844, ZNF880, ZNF93, ZNF99, ZNRF3, ZNRF4, ZPBP2, ZRSR2, ZSWIM5, ZXDC, ZZEF1 |
| **Diabetes (diabetes mellitus)**  2,624 affected genes | A1BG, A2M, AAAS, AACS, AANAT, ABCA1, ABCA8, ABCB1, ABCC8, ABCC9, ABCD1, ABCD3, ABCF1, ABCG1, ABCG2, ABCG5, ABCG8, ABHD16A, ABI2, ABO, ABT1, ACAA1, ACAA2, ACACA, ACACB, ACAD10, ACD, ACE, ACE2, ACO1, ACO2, ACSL1, ACSM2A, ACSM3, ACSM5, ACTA2, ACTRT3, ACVR1, ACVR1B, ACVR1C, ACVR2A, ACVR2B, ADAD1, ADAM10, ADAM22, ADAM30, ADAMTS1, ADAMTS12, ADAMTS14, ADAMTS16, ADAMTS18, ADAMTS19, ADAMTS2, ADAMTS20, ADAMTS3, ADAMTS4, ADAMTS5, ADAMTS6, ADAMTS7, ADAMTS8, ADAMTS9, ADCY1, ADCY5, ADCY9, ADD1, ADGRD1, ADGRL2, ADGRL3, ADIPOQ, ADIPOR1, ADIPOR2, ADK, ADORA1, ADORA2B, ADRA1A, ADRA1B, ADRA1D, ADRA2A, ADRA2B, ADRA2C, ADRB1, ADRB2, ADRB3, AEBP1, AGAP1, AGER, AGPAT1, AGPAT2, AGT, AGTR1, AGTR2, AHI1, AHSG, AIF1, AIM2, AIP, AIRE, AKR1B1, AKR1C3, AKR1E2, AKT1, AKT2, ALAS2, ALB, ALDH1A1, ALDH1L1, ALDH2, ALMS1, ALOX12, ALOX15, ALOX5, ALOX5AP, ALPK2, ALPL, AMACR, AMBP, AMPD1, AMY2A, AMY2B, ANAPC10, ANAPC13, ANAPC5, ANGPT4, ANGPTL4, ANK1, ANK2, ANKRD27, ANKRD44, ANKRD50, ANO10, ANXA1, ANXA11, ANXA2, ANXA5, ANXA7, AOC1, AOC3, AP1S1, AP3S2, AP4B1, APC2, APLN, APOA1, APOA2, APOA4, APOA5, APOB, APOC1, APOC2, APOC3, APOC4, APOD, APOE, APOF, APOH, APOL1, APOM, APP, APPL1, AQP1, AQP5, AR, ARAP1, ARHGAP15, ARHGAP22, ARHGAP8/PRR5-ARHGAP8, ARHGEF11, ARHGEF15, ARHGEF6, ARL1, ARL6, ARMC12, ARMC5, ARR3, ARTN, ASB2, ASB3/GPR75-ASB3, ASCC3, ASIP, ATAT1, ATF2, ATF4, ATF6, ATM, ATP10A, ATP10B, ATP1A2, ATP1B1, ATP2A2, ATP2A3, ATP2C2, ATP4A, ATP4B, ATP6AP2, ATP8B1, ATXN1, ATXN10, ATXN2, ATXN3, ATXN7, ATXN8OS, AVEN, AVPR2, AXIN2, AZGP1, B2M, BACE1, BACE2, BACH2, BAD, BAG6, BAK1, BANK1, BATF3, BAX, BAZ1B, BBS1, BBS10, BBS2, BBS7, BCAR1, BCL11A, BCL2, BCL2L1, BCL2L11, BDKRB2, BDNF, BEAN1, BGLAP, BHMT, BID, BLK, BLM, BMP2, BMP4, BMP7, BMPR1B, BMPR2, BNC2, BPI, BRAF, BRAP, BRCA2, BRD2, BSCL2, BTC, BTG2, BTN2A1, BTN3A1, BTN3A2, BTN3A3, BTNL2, C11orf65, C1QA, C1QB, C1QC, C1S, C1orf162, C2, C2CD4A, C2CD4B, C3, C3AR1, C3orf35, C3orf67, C4A/C4B, C5, C5orf46, C6orf10, C6orf15, C6orf47, C6orf48, C9, CA1, CA12, CA13, CA14, CA2, CA3, CA4, CA5A, CA5B, CA6, CA7, CA9, CACNA1A, CACNA1B, CACNA1C, CACNA1D, CACNA1E, CACNA1F, CACNA1G, CACNA1H, CACNA1I, CACNA1S, CACNA2D1, CACNA2D2, CACNA2D3, CACNA2D4, CACNB1, CACNB2, CACNB3, CACNB4, CACNG1, CACNG2, CACNG3, CACNG4, CACNG5, CACNG6, CACNG7, CACNG8, CADM1, CALCR, CALR, CAMLG, CAMTA1, CANX, CAPG, CAPN10, CAPN3, CAPSL, CARMIL1, CARS, CARTPT, CASP1, CASP10, CASP2, CASP3, CASP4, CASP8, CASQ1, CASR, CAT, CATSPER1, CATSPER2, CATSPER3, CATSPER4, CAV1, CAVIN1, CBARP, CBLB, CBS/CBSL, CCDC12, CCDC138, CCDC28B, CCDC68, CCDC88C, CCL11, CCL16, CCL19, CCL2, CCL22, CCL3, CCL3L3, CCL4, CCL5, CCNB1IP1, CCND1, CCND2, CCND3, CCNE1, CCNE2, CCNG1, CCR1, CCR2, CCR5, CCR6, CCR7, CCR8, CCR9, CCRL2, CD101, CD14, CD180, CD19, CD1D, CD200, CD22, CD226, CD24, CD247, CD27, CD274, CD28, CD300A, CD300C, CD34, CD36, CD38, CD3D, CD3E, CD3G, CD4, CD40, CD40LG, CD44, CD5, CD52, CD7, CD74, CD79B, CD80, CD84, CD86, CD96, CDC123, CDC23, CDC42, CDC42EP3, CDH1, CDH13, CDH17, CDK11A, CDK4, CDKAL1, CDKN1A, CDKN1B, CDKN1C, CDKN2A, CDKN2B, CDKN2B-AS1, CDKN2C, CDON, CDS1, CDSN, CEACAM1, CEACAM16, CEACAM18, CEACAM19, CEACAM20, CEACAM21, CEACAM3, CEACAM4, CEACAM5, CEACAM6, CEACAM7, CEACAM8, CEBPD, CEL, CELSR2, CEP19, CEP295, CES2, CETP, CFB, CFH, CFTR, CGA, CHEK2, CHI3L1, CHN2, CHP1, CHRD, CHRM1, CHRM2, CHRM3, CHRM4, CHRM5, CHRNA1, CHRNA4, CHRNA7, CHRNB2, CHUK, CIB2, CIDEC, CIITA, CILP2, CISD2, CISH, CKB, CKM, CKMT1A/CKMT1B, CKMT2, CLCN1, CLEC12A, CLEC16A, CLEC2B, CLEC2D, CLEC3B, CLEC4A, CLEC6A, CLEC9A, CLIC2, CLIC5, CLPS, CLPTM1, CLTCL1, CLU, CLYBL, CMAHP, CMKLR1, CNBP, CNDP1, CNGA1, CNGB1, CNR1, CNR2, CNTF, CNTN4, CNTNAP2, CNTNAP5, COBLL1, COL10A1, COL11A1, COL11A2, COL12A1, COL13A1, COL14A1, COL15A1, COL16A1, COL17A1, COL18A1, COL19A1, COL1A1, COL1A2, COL21A1, COL22A1, COL23A1, COL24A1, COL25A1, COL27A1, COL2A1, COL3A1, COL4A1, COL4A2, COL4A3, COL4A4, COL4A5, COL4A6, COL5A1, COL5A2, COL5A3, COL6A1, COL6A2, COL6A3, COL7A1, COL8A1, COL8A2, COL9A1, COL9A2, COL9A3, COMT, COPS2, COPS3, COPS5, COPS8, CP, CPE, CPLX2, CPNE4, CPT1A, CPT1B, CPT1C, CPVL, CR1, CR2, CRABP1, CRABP2, CREB1, CREB3, CREB3L3, CREB3L4, CREB5, CREBBP, CRELD1, CRELD2, CREM, CRH, CRHBP, CRHR1, CRIP1, CRLF2, CRP, CRX, CRYAB, CSF2, CSF3R, CSGALNACT1, CSGALNACT2, CSH1/CSH2, CSHL1, CSN3, CSNK1D, CTBP1, CTC1, CTGF, CTH, CTLA4, CTNNB1, CTNNBL1, CTNS, CTRC, CTSB, CTSH, CTSK, CTSL, CTSS, CTSV, CTSW, CUBN, CUX2, CX3CR1, CXADR, CXCL10, CXCL12, CXCL13, CXCL16, CXCL5, CXCL8, CXCR3, CXCR4, CXCR5, CXCR6, CYB5A, CYB5R4, CYBA, CYBB, CYBRD1, CYC1, CYP11A1, CYP11B1, CYP11B2, CYP19A1, CYP21A2, CYP24A1, CYP27B1, CYP2C19, CYP2C8, CYP2C9, CYP2D6, CYP2E1, CYP2R1, CYP4F2, CYP7A1, CYSLTR1, CYTL1, DAB1, DAB2IP, DAD1, DAPK1, DAPL1, DBI, DCAF17, DCAKD, DCD, DCK, DCLRE1C, DDAH1, DDAH2, DDOST, DDR1, DDX25, DDX39B, DDX5, DDX60, DDX60L, DEAF1, DECR1, DGKB, DGUOK, DHCR7, DHFR, DHRS2, DHX16, DICER1, DIO2, DIP2C, DIS3L2, DISP1, DKC1, DKK1, DKK3, DLGAP2, DLL1, DMBX1, DMD, DNAH10, DNAJC21, DNAJC3, DNER, DNLZ, DNMT1, DNMT3A, DOCK6, DOCK7, DPCR1, DPEP1, DPP4, DPP6, DPYS, DPYSL3, DRD1, DRD2, DRD3, DRD4, DRD5, DUSP1, DUSP23, DUSP9, DYM, DYRK1A, DYRK2, DYSF, E2F1, E2F2, E2F3, E2F7, ECEL1, ECHS1, ECM1, EDA, EDA2R, EDARADD, EDEM3, EDN1, EDNRA, EDNRB, EEA1, EEF2, EGFR, EGR1, EHMT2, EIF2AK3, EIF2AK4, EIF2S3, ELMO1, ELN, ELOVL5, ENAH, ENO2, ENPEP, ENPP1, EP300, EPAS1, EPB41L3, EPHA4, EPO, EPOR, ERBB3, ERCC4, ERCC6, ERN1, ESR1, ESR2, ESRRA, ESRRG, ETFB, ETFDH, ETNK2, ETS2, EVA1C, EVI2A, EXPH5, EYA2, F10, F12, F13A1, F2, F2R, F3, F5, F7, FA2H, FAAH, FABP1, FABP2, FABP4, FABP6, FABP7, FADD, FADS2, FAF1, FAH, FAM111B, FAM19A1, FAM26F, FAM60A, FAS, FASLG, FASN, FBL, FBLN2, FBN1, FBP1, FBXO11, FBXO28, FBXO3, FBXW12, FCER1G, FCER2, FCGR1A, FCGR1B, FCGR2A, FCGR2B, FCGR2C, FCGR3A/FCGR3B, FCGRT, FCMR, FCRL1, FGA, FGB, FGF1, FGF14, FGF19, FGF8, FGF9, FGFR1, FGFR2, FGFR3, FGFR4, FGL1, FHOD3, FKBP15, FKBP1A, FLAD1, FLOT1, FLT1, FMN2, FMR1, FN1, FN3K, FOS, FOXA1, FOXA2, FOXC1, FOXC2, FOXH1, FOXL1, FOXO1, FOXO3, FOXP1, FOXP3, FREM2, FRMD3, FRMD4B, FRZB, FST, FSTL4, FTH1, FTL, FTO, FUT2, FXN, FXYD2, FXYD5, FYN, G6PC2, G6PD, GAA, GABBR1, GABRA1, GABRA2, GABRA3, GABRA4, GABRA5, GABRA6, GABRB1, GABRB2, GABRB3, GABRD, GABRE, GABRG1, GABRG2, GABRG3, GABRP, GABRQ, GABRR1, GABRR2, GABRR3, GAD1, GAD2, GAL, GALNT2, GALNT6, GALP, GALT, GANAB, GANC, GAP43, GAS1, GAS2L1, GAS6, GATA1, GATA4, GATA5, GATA6, GBP2, GBP4, GBP6, GBX1, GC, GCDH, GCG, GCGR, GCK, GCKR, GCLC, GCLM, GDF1, GDF11, GDF15, GDF2, GFAP, GFM2, GFPT1, GFPT2, GFRA1, GGH, GGPS1, GGT1, GGT2/LOC102724197, GGT5, GGT6, GGT7, GGTLC1, GGTLC2, GH1, GH2, GHR, GHRH, GHRHR, GHRL, GHSR, GIP, GIPR, GJA1, GJA4, GJB3, GJB4, GLA, GLB1, GLI2, GLIPR2, GLIS1, GLIS2, GLIS3, GLP1R, GLRA3, GLRX5, GMDS, GNAI2, GNAS, GNAT1, GNAT2, GNB1, GNB3, GNB4, GNGT1, GNPAT, GNRH1, GNRHR, GOLPH3, GOT1, GOT1L1, GOT2, GPANK1, GPC3, GPC4, GPC5, GPD2, GPLD1, GPM6A, GPNMB, GPR1, GPR101, GPR141, GPR18, GPR39, GPR65, GPSM1, GPSM3, GPT, GPT2, GPX1, GPX2, GPX3, GPX4, GPX5, GPX6, GPX7, GPX8, GRB14, GRB2, GREM1, GRHL2, GRIA4, GRID2, GRIK1, GRIK2, GRIK3, GRIK4, GRIK5, GRIN1, GRIN2A, GRIN2B, GRIN2C, GRIN2D, GRIN3A, GRIN3B, GRINA, GRK1, GRK4, GSC, GSK3A, GSK3B, GSR, GSS, GSTA1, GSTA4, GSTK1, GSTM1, GSTM2, GSTO1, GSTP1, GSTT1, GSTT2/GSTT2B, GSTZ1, GTF2H4, GUCA1B, GUCY2C, GYPA, GYS1, GZMA, H19, H2BFM, HAAO, HACD2, HADH, HAMP, HAND1, HAPLN1, HAVCR1, HAVCR2, HBA1/HBA2, HBB, HBD, HBE1, HBG1, HBG2, HBQ1, HBS1L, HBZ, HCAR2, HCAR3, HCFC1, HCG22, HCG4, HCG9, HCLS1, HCN1, HCP5, HCST, HDAC1, HDAC10, HDAC11, HDAC2, HDAC3, HDAC4, HDAC5, HDAC6, HDAC7, HDAC8, HDAC9, HERC6, HES1, HES5, HEXA, HEXB, HEXDC, HFE, HFE2, HGF, HHAT, HHEX, HIPK1, HIST1H2BF, HIST1H2BO, HIST1H3B, HIST1H3I, HIST1H4L, HK2, HLA-A, HLA-B, HLA-C, HLA-DMA, HLA-DMB, HLA-DOA, HLA-DOB, HLA-DPA1, HLA-DPB1, HLA-DQA1, HLA-DQA2, HLA-DQB1, HLA-DQB2, HLA-DRA, HLA-DRB1, HLA-DRB3, HLA-DRB4, HLA-DRB5, HLA-E, HLA-F, HLA-G, HLA-L, HMBOX1, HMG20A, HMGA1, HMGA2, HMGCL, HMGCR, HMGCS2, HMGN4, HNF1A, HNF1B, HNF4A, HNF4G, HOXA3, HOXC8, HP, HPGD, HPN, HPR, HPSE, HPSE2, HPX, HRAS, HRH1, HRH3, HS3ST1, HS6ST3, HSD11B1, HSD17B1, HSD17B13, HSD3B1, HSD3B2, HSF1, HSPA1A/HSPA1B, HSPA1L, HSPA5, HSPB1, HTR1A, HTR2A, HTR2B, HTR2C, HTR3A, HTR3B, HTR3C, HTR3D, HTR3E, HTR4, HTR6, HTT, HUWE1, HYAL1, HYAL2, HYAL3, HYAL4, IAPP, ICA1, ICAM1, ICAM2, ICAM3, ICAM4, ICAM5, ICOS, ID1, ID3, IDE, IER3IP1, IFI16, IFI44, IFIH1, IFIT1, IFIT1B, IFIT2, IFIT3, IFITM3, IFNA1/IFNA13, IFNA10, IFNA14, IFNA16, IFNA17, IFNA2, IFNA21, IFNA4, IFNA5, IFNA6, IFNA7, IFNA8, IFNAR1, IFNB1, IFNG, IFNGR1, IFNGR2, IFNL2, IFNL3, IFT172, IGF1, IGF1R, IGF2, IGF2-AS, IGF2BP2, IGF2R, IGFALS, IGFBP1, IGFBP2, IGFBP3, IGFBP5, IGFBP7, IKZF3, IL10, IL10RA, IL11, IL12A, IL12B, IL13, IL15, IL15RA, IL17A, IL17B, IL17C, IL17D, IL17F, IL18, IL18BP, IL18RAP, IL1A, IL1B, IL1F10, IL1R1, IL1R2, IL1RN, IL2, IL21, IL21R, IL22, IL25, IL2RA, IL2RB, IL3, IL33, IL36A, IL36B, IL36G, IL36RN, IL37, IL4, IL4R, IL5, IL5RA, IL6, IL6R, IL6ST, IL7, IL7R, IMMP2L, IMMT, IMPDH1, IMPDH2, INPP5K, INPPL1, INS, INS-IGF2, INSL3, INSM1, INSR, IPCEF1, IPO11, IPW, IRAK3, IRF1, IRF4, IRF7, IRGM, IRS1, IRS2, IRS4, ISL1, ITCH, ITGA2, ITGA2B, ITGA3, ITGA4, ITGAE, ITGAM, ITGAV, ITGAX, ITGB2, ITGB3, ITGB5, ITGB7, ITIH4, ITLN1, ITPR1, ITPR2, ITPR3, JAG1, JAK1, JAK2, JAK3, JAML, JAZF1, JUN, KALRN, KAT2B, KATNB1, KAZN, KCNA4, KCNA5, KCNA7, KCNAB3, KCNC3, KCNE1, KCNE2, KCNG2, KCNH2, KCNH7, KCNJ1, KCNJ11, KCNJ12, KCNJ15, KCNJ2, KCNJ5, KCNJ6, KCNJ8, KCNJ9, KCNK10, KCNK16, KCNK3, KCNN3, KCNQ1, KCNQ1OT1, KCTD1, KCTD16, KDR, KERA, KHDRBS1, KIAA0825, KIAA1109, KIAA1549L, KIAA1671, KIF11, KIF1A, KIF6, KIR2DL1/KIR2DL3, KIR2DL2, KIR2DL4, KIR2DL5A, KIR2DL5B, KIR2DS3, KIR2DS4 , KIR3DL1, KIR3DL2, KIR3DL3, KIR3DS1, KIRREL3, KIT, KITLG, KLF10, KLF11, KLF14, KLF15, KLF4, KLF6, KLF7, KLHL1, KLHL42, KLRA1P, KLRB1, KMO, KNG1, KRAS, KRIT1, KRT17, KRT4, KRT8, L1CAM, L3HYPDH, LARGE1, LARS2, LAT, LBR, LCAT, LCK, LCN2, LCP1, LDLR, LDLRAP1, LEF1, LEP, LEPR, LGALS3, LGALS9B, LHB, LHCGR, LIF, LIG4, LILRB1, LILRB3, LILRB4, LIMK1, LIMK2, LIN9, LINC00271, LINC01104, LINC01619, LIPA, LIPC, LIPE, LIPG, LMNA, LMNB2, LOC101927026, LOC101927668, LOC101928423, LOC105370647, LOC105370656, LOC105377632, LOC107985988, LOC339529, LOC646588, LOR, LOXL3, LPA, LPAL2, LPIN2, LPL, LPP, LRBA, LRIG1, LRP1, LRP11, LRP1B, LRP2, LRP5, LRP6, LRP8, LRRC32, LSM2, LST1, LTA, LTBR, LTC4S, LY6D, LY6G5B, LY6G5C, LY6G6C, LY6G6D, LY86, LYRM4, LYZ, M6PR, MACF1, MADD, MAEA, MAF, MAFA, MAGEL2, MAGI2, MAGI3, MAN2B1, MAOA, MAOB, MAP2K1, MAP3K3, MAP4K4, MAPK1, MAPK10, MAPK14, MAPK3, MAPK8IP1, MAPK9, MAPKAPK5, MAPKAPK5-AS1, MAPT, MARCH6, MARCH8, MBD4, MBL2, MC2R, MC3R, MC4R, MCCD1, MCF2L2, MCHR1, MDC1, MDM2, MECP2, MED27, MEF2B, MEFV, MEN1, MERTK, MGAM, MGEA5, MGP, MGST1, MGST2, MGST3, MIA3, MICA, MICB, MIF, MIPOL1, MKKS, MKRN3, MLXIPL, MME, MMP1, MMP11, MMP12, MMP13, MMP14, MMP15, MMP16, MMP17, MMP19, MMP2, MMP21, MMP27, MMP3, MMP7, MMP9, MNX1, MOB2, MOG, MORN1, MPEG1, MPG, MPHOSPH9, MPO, MPZ, MR1, MRAP, MRAS, MRC1, MRPS35, MS4A1, MS4A2, MS4A6A, MSC, MSH2, MSH5, MSR1, MSTN, MT-ATP6, MT-ATP8, MT-CO1, MT-CO2, MT-CO3, MT-CYB, MT-ND1, MT-ND2, MT-ND3, MT-ND4, MT-ND4L, MT-ND5, MT-ND6, MT-RNR1, MT-RNR2, MT-TC, MT-TE, MT-TF, MT-TH, MT-TI, MT-TK, MT-TL1, MT-TL2, MT-TM, MT-TQ, MT-TR, MT-TS1, MT-TS2, MT-TT, MT-TY, MT1A, MT1G, MT2A, MT3, MTAP, MTHFD1, MTHFD1L, MTHFR, MTNR1A, MTNR1B, MTOR, MTR, MTRR, MTTP, MUC1, MUC21, MYBPC2, MYBPC3, MYC, MYH11, MYH8, MYH9, MYO1B, MYO1F, MYO3B, MYO7A, MYO9B, MYOG, MYSM1, MYT1L, MZB1, NAA25, NADSYN1, NAE1, NAGLU, NANOS3, NCALD, NCF1, NCF2, NCK1, NDN, NDP, NDST1, NDST3, NDUFA1, NDUFA10, NDUFA11, NDUFA12, NDUFA13, NDUFA2, NDUFA3, NDUFA4, NDUFA4L2, NDUFA5, NDUFA6, NDUFA7, NDUFA8, NDUFA9, NDUFAB1, NDUFAF1, NDUFAF2, NDUFB1, NDUFB10, NDUFB11, NDUFB2, NDUFB3, NDUFB4, NDUFB5, NDUFB6, NDUFB7, NDUFB8, NDUFB9, NDUFS1, NDUFS2, NDUFS3, NDUFS4, NDUFS5, NDUFS6, NDUFS7, NDUFS8, NDUFV1, NDUFV2, NDUFV3, NEBL, NECTIN2, NEDD4, NELFE, NEUROD1, NEUROG3, NFATC2, NFIX, NFKB1, NFKBIA, NGF, NHP2, NISCH, NKAPL, NKX2-2, NKX2-5, NKX6-1, NLRP1, NLRP2, NLRP3, NMT2, NNMT, NNT, NOC4L, NOD2, NODAL, NOG, NOP10, NOP53, NOS1, NOS1AP, NOS2, NOS3, NOTCH1, NOTCH2, NOTCH3, NOTCH4, NOX4, NPAS3, NPC1L1, NPHS1, NPPA, NPPB, NPY, NQO1, NR0B1, NR0B2, NR1H3, NR1H4, NR2E3, NR2F2, NR3C1, NR3C2, NR4A1, NR4A2, NR4A3, NR5A1, NRAS, NRF1, NRG3, NRL, NRSN1, NRXN3, NSD1, NSF, NT5E, NTRK1, NTRK2, NUB1, NUP155, NXN, OAS1, OCA2, OFD1, OGG1, OLR1, ONECUT1, OPN1SW, OPRD1, OPRK1, OPRM1, OR11A1, OR12D2, OR12D3, OR13D1, OR2B3, OR5K3, ORM1, ORM2, OSM, OTX2, OXT, P2RX7, P2RY1, P2RY10, P2RY12, P2RY13, PAK1, PAK5, PARD3B, PARG, PARK7, PARN, PARP1, PARP14, PASK, PAX2, PAX4, PAX6, PAX8, PBX3, PCBD1, PCDH10, PCDH15, PCK1, PCNT, PCSK1, PCSK2, PCSK6, PCSK7, PCSK9, PCYOX1, PCYT1A, PDCD1, PDCD1LG2, PDCD4, PDE11A, PDE1A, PDE1B, PDE2A, PDE3A, PDE3B, PDE4A, PDE4B, PDE4C, PDE4D, PDE5A, PDE6A, PDE6B, PDE6C, PDE7A, PDE7B, PDE8A, PDE8B, PDGFC, PDGFRA, PDGFRB, PDGFRL, PDHX, PDIA6, PDK2, PDLIM4, PDX1, PDYN, PDZD2, PDZK1, PEA15, PEBP1, PECAM1, PELI2, PFKFB3, PGF, PGM1, PGR, PHACTR1, PHACTR3, PHLDB1, PHLPP1, PHTF1, PIAS3, PIEZO2, PIGA, PIK3C2G, PIK3CG, PIK3R1, PILRB, PIP, PITPNM1, PJA2, PKHD1, PKLR, PKM, PKNOX1, PLA2G6, PLA2G7, PLA2R1, PLAGL1, PLAT, PLBD1, PLCB1, PLCD1, PLCG2, PLD4, PLEK, PLEKHG4, PLIN1, PLN, PLPP3, PLPP4, PLSCR1, PLTP, PLXDC2, PLXNA4, PLXND1, PNLIPRP3, PNPLA2, PNPLA3, PNPLA6, POC1A, POLA1, POLD1, POLG, POLI, POLR2M, POMC, PON1, PON2, PON3, POR, POT1, POU5F1, POU6F2, PPARA, PPARD, PPARG, PPARGC1A, PPARGC1B, PPEF2, PPIL6, PPM1F, PPP1R11, PPP1R3A, PPP2CA, PPP2R2B, PPP3CA, PPP3CB, PPP3CC, PPP3R1, PPP3R2, PPT2, PRAG1, PRC1, PRDM10, PRDM5, PRDX6, PRF1, PRIM1, PRIM2, PRKAA1, PRKAA2, PRKACA, PRKAG2, PRKAR1A, PRKCA, PRKCB, PRKCD, PRKCE, PRKCI, PRKCQ, PRKDC, PRL, PROC, PROK1, PROK2, PROS1, PROX1, PRPH2, PRR16, PRR5, PRRC2A, PRRX2, PRSS1, PRSS16, PRUNE2, PSMA1, PSMA2, PSMA3, PSMA4, PSMA5, PSMA6, PSMA7, PSMA8, PSMB1, PSMB10, PSMB11, PSMB2, PSMB3, PSMB4, PSMB5, PSMB6, PSMB7, PSMB8, PSMB9, PSMC1, PSMC2, PSMC3, PSMC4, PSMC5, PSMC6, PSMD1, PSMD10, PSMD11, PSMD12, PSMD13, PSMD14, PSMD2, PSMD3, PSMD4, PSMD5, PSMD6, PSMD7, PSMD8, PSMD9, PSME1, PSME2, PSME3, PSME4, PSMF1, PSORS1C1, PSRC1, PTAFR, PTCH1, PTEN, PTF1A, PTGDS, PTGER1, PTGER2, PTGER3, PTGER4, PTGFR, PTGIR, PTGIS, PTGS1, PTGS2, PTH, PTHLH, PTPMT1, PTPN1, PTPN11, PTPN2, PTPN22, PTPN6, PTPRC, PTPRD, PTPRM, PTPRN, PTPRN2, PTPRO, PTTG1, PVR, PVT1, PXDNL, PYCARD, PYY, QPRT, RAB5A, RAB5B, RAB5C, RAC1, RAC2, RAD54B, RAET1G, RAET1L, RAF1, RAG2, RAMP1, RAMP2, RAMP3, RAPGEF4, RARB, RARRES2, RB1, RBBP6, RBFOX1, RBL1, RBL2, RBM19, RBMS1, RBP1, RBP2, RBP3, RBP4, RBP5, RBP7, RCAN1, RCVRN, RDH12, RDH5, REG1A, REL, REN, REST, RET, RETN, RFC1, RFX6, RGS1, RGS2, RGS5, RGS7, RHO, RHOB, RHOBTB3, RHOU, RIDA, RINL, RNASE6, RNF11, RNF138, RNF220, RNF39, RNF5, ROBO2, ROM1, RP1, RPGRIP1L, RPH3A, RPS6KA3, RREB1, RRM1, RRM2, RRM2B, RS1, RSAD2, RSBN1, RSPO4, RTEL1, RTP4, RUNX3, RXRB, RXRG, RYR3, S100A1, S100A10, S100A11, S100A12, S100A13, S100A14, S100A16, S100A2, S100A3, S100A4, S100A5, S100A6, S100A7, S100A7A, S100A8, S100A9, S100B, S100G, S100P, S100Z, SAA1, SAA2, SAA2-SAA4, SAA4, SAG, SALL3, SAMD12, SAMD4A, SAMD9L, SARS2, SBDS, SBF2, SCAP, SCARB1, SCARB2, SCD, SCG5, SCGB1A1, SCN10A, SCN11A, SCN1A, SCN1B, SCN2A, SCN2B, SCN3A, SCN3B, SCN4A, SCN4B, SCN5A, SCN7A, SCN8A, SCN9A, SCNN1A, SCNN1B, SCYL1, SDF2L1, SDHB, SDHD, SDK2, SDR16C6P, SEC11A, SELE, SELENOP, SELL, SELP, SEMA5A, SEMA6B, SERPINA1, SERPINA3, SERPINB4, SERPINC1, SERPIND1, SERPINE1, SERPINF1, SERPINF2, SERPING1, SETD7, SEZ6L, SF1, SFI1, SFTPD, SGCD, SGCZ, SGF29, SGK1, SH2B1, SH2B3, SH2D1A, SH2D1B, SH2D2A, SHBG, SHH, SHMT1, SIAE, SIGLEC10, SIGMAR1, SIRPB1, SIRT1, SIRT3, SIRT6, SIX3, SKIV2L, SLC11A2, SLC12A1, SLC12A3, SLC13A1, SLC14A1, SLC16A11, SLC16A13, SLC16A2, SLC17A1, SLC17A3, SLC17A4, SLC19A2, SLC19A3, SLC22A1, SLC22A11, SLC22A12, SLC22A15, SLC22A2, SLC22A3, SLC22A5, SLC22A6, SLC24A1, SLC24A3, SLC26A1, SLC27A4, SLC29A3, SLC2A1, SLC2A10, SLC2A2, SLC2A4, SLC2A9, SLC30A8, SLC39A1, SLC3A2, SLC40A1, SLC44A3, SLC44A4, SLC44A5, SLC47A2, SLC5A2, SLC6A2, SLC6A20, SLC6A3, SLC6A4, SLC7A11, SLC9A1, SLC9A9, SLCO1B1, SLITRK5, SMAD1, SMAD2, SMAD3, SMAD4, SMAD5, SMAD7, SMAD9, SMARCA4, SMC3, SMURF2, SNAP25, SNCA, SNORD107, SNRPN, SNTB2, SNX4, SOAT1, SOCS1, SOCS2, SOCS3, SOD1, SOD2, SOD3, SORBS1, SORCS1, SORCS2, SORT1, SOS1, SOX1, SOX2, SP1, SP140, SPAG16, SPATA5, SPG7, SPHK1, SPIB, SPINK1, SPINK4, SPN, SPOCK1, SPP1, SPRED1, SPRY1, SPRY2, SPRY4, SPTA1, SPTBN2, SQSTM1, SRC, SREBF1, SREBF2, SRPRB, SRR, SSR1, SSTR1, SSTR2, SSTR3, SSTR4, SSTR5, ST3GAL6, ST6GAL1, STAP1, STAR, STAT1, STAT2, STAT3, STAT4, STAT5A, STAT5B, STAT6, STATH, STIL, STK11, STK19, STK32B, STK32C, STMN2, STRN3, STX1A, SUFU, SULF1, SUMO3, SUMO4, SUV39H1, SUV39H2, SYN1, SYN2, SYNE3, TAF13, TAF1B, TANGO6, TAP1, TAP2, TARDBP, TARP, TAS2R38, TBC1D4, TBX21, TBX5, TBXA2R, TCF19, TCF21, TCF24, TCF7, TCF7L2, TCN1, TCN2, TDGF1, TEK, TERC, TERF1, TERT, TF, TFAM, TFG, TFPI, TFR2, TFRC, TG, TGFB1, TGFB2, TGFB3, TGFBI, TGFBR1, TGFBR2, TGFBR3, TGIF1, TGM2, TGM6, TH, THADA, THBD, THBS2, THEM4, THEMIS, THPO, THRA, THRB, THY1, TIMP1, TIMP2, TIMP3, TIMP4, TINF2, TLE1, TLE4, TLR1, TLR10, TLR2, TLR3, TLR4, TLR5, TLR6, TLR7, TLR8, TLR9, TM6SF2, TMEFF2, TMEM116, TMEM140, TMEM154, TMEM18, TMEM212, TMEM240, TMEM71, TMPO, TMPRSS6, TMTC1, TNC, TNF, TNFAIP3, TNFRSF11A, TNFRSF11B, TNFRSF13B, TNFRSF13C, TNFRSF1A, TNFRSF1B, TNFRSF21, TNFRSF4, TNFRSF9, TNFSF10, TNFSF11, TNFSF13B, TNFSF4, TNFSF8, TNNT2, TNS3, TNXB, TOMM40, TOP2A, TOP2B, TOX, TP53, TP53INP1, TP63, TPBG, TPH2, TPMT, TPP1, TRA2B, TRAFD1, TRAK2, TRIB3, TRIM10, TRIM13, TRIM2, TRIM26, TRIM27, TRIM31, TRIM37, TRIM40, TRIM44, TRIO, TRMT10A, TRPA1, TRPC3, TRPM6, TRPV1, TSFM, TSHR, TSPAN12, TSPAN18, TSPAN5, TSPAN8, TSPO, TTBK2, TTC27, TTC7A, TTPA, TTR, TUB, TUBA1A, TUBA1C, TUBA3C/TUBA3D, TUBA4A, TUBA8, TUBB, TUBB1, TUBB2A, TUBB3, TUBB4A, TUBB4B, TUBD1, TUBE1, TUBG1, TUBG2, TULP1, TUSC3, TWNK, TWSG1, TXN, TYMS, TYRO3, TYROBP, UBAC2, UBASH3A, UBB, UBD, UBE2A, UBE2B, UBE2E1, UBE2E2, UBE2E3, UBE2G1, UBE2K, UBE2M, UBE2N, UBE2Q1, UBE3C, UBR1, UBR7, UBXN7, UCHL1, UCK2, UCP1, UCP2, UCP3, UFC1, UGT1A1, UGT1A6, UGT2B7, UMOD, UQCR10, UQCR11, UQCRB, UQCRC1, UQCRC2, UQCRFS1, UQCRH, UQCRQ, USB1, USF1, USP1, USP12, USP16, USP25, USP46, USP7, USP8, UTS2, VANGL1, VARS2, VAV2, VCAM1, VDR, VEGFA, VEGFB, VEGFC, VEGFD, VEPH1, VHL, VIP, VKORC1, VLDLR, VPREB3, VPS13C, VPS26A, VTCN1, VTN, VWA3B, VWA7, VWF, WAC, WDR12, WDR72, WFDC1, WFS1, WISP1, WNT1, WNT16, WNT2B, WNT4, WNT6, WRAP53, WRN, WSB2, XAF1, XBP1, XCL1, XDH, XRCC1, XRCC3, XRCC4, YBX3, ZBED3, ZBP1, ZBTB1, ZBTB12, ZBTB16, ZBTB7A, ZBTB7C, ZEB2, ZFAND3, ZFAND6, ZFP36, ZFP57, ZGLP1, ZHX2, ZIC1, ZIC2, ZKSCAN3, ZKSCAN4, ZKSCAN8, ZMAT4, ZMIZ1, ZMPSTE24, ZMYM2, ZNF165, ZNF274, ZNF311, ZNF615, ZNF665, ZNF7, ZNF705A, ZNF708, ZNHIT3, ZNRD1, ZNRD1ASP, ZNRF1, ZPBP, ZPR1, ZSCAN12, ZSCAN31 |
| **Hypertension**  2,818 affected genes | A1BG, ABCA1, ABCA3, ABCA8, ABCB1, ABCB11, ABCB4, ABCB6, ABCC1, ABCC3, ABCC4, ABCC5, ABCC6, ABCC8, ABCC9, ABCD3, ABCD4, ABCF2, ABCG2, ABCG5, ABCG8, ABHD4, ABL1, ABLIM1, ABO, ACAA1, ACACB, ACBD3, ACE, ACE2, ACHE, ACKR1, ACLY, ACMSD, ACOX1, ACOX2, ACSM3, ACTA2, ACTC1, ACTG2, ACTN4, ACTR3, ACTR5, ACTR6, ACVR1C, ACVR2A, ACVRL1, ACY3, ADA, ADA2, ADAM17, ADAMTS13, ADAMTS7, ADAMTS9, ADAMTSL1, ADAMTSL4, ADCY4, ADCY5, ADCY6, ADD1, ADD2, ADGRF5, ADGRL1, ADH1A, ADIPOQ, ADIRF, ADM, ADORA2A, ADORA2B, ADRA1A, ADRA1B, ADRA1D, ADRA2A, ADRA2B, ADRA2C, ADRB1, ADRB2, ADRB3, AFF3, AFTPH, AGAP1, AGBL1, AGER, AGGF1, AGO1, AGO2, AGPAT2, AGT, AGTR1, AGTR2, AGXT2, AHI1, AHR, AHSG, AIP, AIRE, AK9, AKR1C3, AKT3, ALAD, ALAS2, ALB, ALDH1A2, ALDH1A3, ALDH2, ALDH3A2, ALDH8A1, ALG10, ALG8, ALG9, ALK, ALKBH3, ALMS1, ALOX12, ALOX5, ALOX5AP, ALPI, ALPL, ALPP, ALPPL2, ALX4, AMBP, AMD1, AMER1, AMFR, AMMECR1, AMPD3, AMY1C , ANG, ANGPT1, ANGPT2, ANGPTL2, ANGPTL3, ANGPTL4, ANGPTL6, ANGPTL8, ANK1, ANKK1, ANKRD27, ANKRD29, ANKRD34A, ANKRD55, ANLN, ANXA1, ANXA11, AOC3, AP3S2, APBB3, APC, APLN, APLNR, APOA1, APOA2, APOA4, APOA5, APOB, APOBEC3A, APOBEC3B, APOBEC3D, APOC1, APOC2, APOC3, APOC4, APOD, APOE, APOF, APOH, APOL1, APOL3, APOM, APTX, AQP7, AQP9, AR, ARAP1, ARFGAP1, ARG1, ARG2, ARHGAP15, ARHGAP24, ARHGAP31, ARHGAP33, ARHGAP8/PRR5-ARHGAP8, ARHGEF1, ARHGEF10, ARHGEF15, ARHGEF19, ARHGEF25, ARHGEF9, ARL13B, ARL15, ARL5B, ARL6, ARL8B, ARMC5, ARNTL, ARPC5, ARRB2, ARSA, ART4, ARTN, ASB4, ASCC3, ASCL1, ASIC1, ASIC2, ASIC3, ASIC4, ASIC5, ASMTL, ASPN, ASS1, ASXL1, ATF1, ATF6, ATL1, ATL3, ATM, ATMIN, ATN1, ATP11A, ATP1A1, ATP1B1, ATP2A2, ATP2A3, ATP2B1, ATP2B3, ATP2B4, ATP2C1, ATP4A, ATP4B, ATP5A1, ATP6AP2, ATP6V0E2-AS1, ATP6V1A, ATP6V1E1, ATP8A1, ATP8B2, ATP8B4, ATPAF2, ATRN, ATRX, ATXN1, ATXN2, AURKA, AVP, AVPR1A, AVPR2, AXIN2, AZGP1, AZIN2, B2M, B4GALT1, B9D1, B9D2, BAAT, BAD, BAK1, BANF1, BANK1, BAP1, BARD1, BAX, BAZ1A, BAZ1B, BBIP1, BBS1, BBS10, BBS12, BBS2, BBS4, BBS5, BBS7, BBS9, BCAM, BCAR1, BCAS1, BCAT1, BCL11A, BCL2, BCL2L1, BCL6, BDKRB2, BDNF, BGLAP, BGLT3, BGN, BHLHE41, BIRC5, BLK, BLM, BMP1, BMP2, BMP4, BMP6, BMP8A, BMPR1A, BMPR1B, BMPR2, BNIP2, BNIP3L, BOK, BORA, BRAF, BRAP, BRCA2, BRS3, BSCL2, BTBD7, BTN2A1, BTNL2, BUB1B, BZW1, C10orf10, C10orf107, C12orf49, C16orf45, C18orf8, C19orf57, C1GALT1, C1QTNF5, C1S, C2CD3, C2CD4A, C3, C3AR1, C3orf18, C3orf52, C4A/C4B, C4orf33, C5, C5orf42, C6orf10, C6orf106, C7orf26, C8orf37, C9, CA1, CA10, CA11, CA12, CA13, CA14, CA2, CA3, CA4, CA5A, CA5B, CA6, CA7, CA8, CA9, CACNA1A, CACNA1B, CACNA1C, CACNA1D, CACNA1E, CACNA1F, CACNA1G, CACNA1H, CACNA1I, CACNA1S, CACNA2D1, CACNA2D2, CACNA2D3, CACNA2D4, CACNB1, CACNB2, CACNB3, CACNB4, CACNG1, CACNG2, CACNG3, CACNG4, CACNG5, CACNG6, CACNG7, CACNG8, CALCOCO1, CALD1, CALML4, CALR, CAMK2B, CAND1, CAP1, CAPN3, CARD16, CARM1, CASP3, CASP8, CASP9, CASR, CAST, CASZ1, CAT, CATSPER1, CATSPER2, CATSPER3, CATSPER4, CAV1, CAV2, CAV3, CAVIN1, CBFB, CBL, CBLB, CBLN1, CBLN2, CBS/CBSL, CBWD2, CBX7, CBY1, CC2D2A, CCBE1, CCDC117, CCDC136, CCDC28B, CCDC50, CCDC74A, CCDC74B, CCDC8, CCDC81, CCDC85A, CCDC86, CCL11, CCL13, CCL15, CCL17, CCL18, CCL2, CCL20, CCL24, CCL26, CCL27, CCL3, CCL4, CCL5, CCL7, CCNA2, CCNB1, CCNC, CCND1, CCND2, CCND3, CCNE1, CCNE2, CCNG1, CCR2, CCR5, CD14, CD163, CD177, CD19, CD1D, CD22, CD226, CD247, CD27, CD2AP, CD34, CD36, CD38, CD3G, CD40LG, CD44, CD46, CD47, CD52, CD55, CD63, CD69, CD80, CD84, CD86, CD9, CDC123, CDC25A, CDC42EP3, CDC7, CDC73, CDCA8, CDH1, CDH13, CDH17, CDK4, CDK6, CDKAL1, CDKN1A, CDKN1B, CDKN2A, CDKN2B-AS1, CDKN2C, CDYL2, CEBPA, CEBPE, CELA1, CELF2, CELF4, CELSR2, CENPK, CEP104, CEP120, CEP164, CEP19, CEP290, CEP41, CERS4, CETP, CFAP126, CFAP69, CFH, CFHR1, CFHR3, CFI, CFL1, CFLAR, CFTR, CGA, CGB1/CGB2, CGB3 , CGB7, CHD7, CHEK2, CHGA, CHI3L2, CHIT1, CHM, CHN2, CHP1, CHRM1, CHRM2, CHRM3, CHRM4, CHRM5, CHRNA1, CHRNA10, CHRNA2, CHRNA3, CHRNA4, CHRNA5, CHRNA6, CHRNA7, CHRNA9, CHRNB1, CHRNB2, CHRNB3, CHRNB4, CHRND, CHRNE, CHRNG, CHST3, CHUK, CIART, CIB2, CIITA, CILP2, CITED2, CLCN6, CLCN7, CLCNKA, CLCNKB, CLDN23, CLEC11A, CLEC12B, CLEC14A, CLEC2B, CLEC4E, CLEC7A, CLIC2, CLIC5, CLIP2, CLOCK, CLPTM1, CLTCL1, CLU, CMIP, CMTM7, CNN3, CNNM1, CNNM2, CNR1, CNTN4, CNTNAP2, CNTNAP5, COBLL1, COCH, COG5, COL12A1, COL16A1, COL18A1, COL1A1, COL1A2, COL3A1, COL4A1, COL4A2, COL4A3, COL4A4, COL4A5, COL4A6, COL5A1, COL5A2, COL6A1, COL6A2, COL6A3, COL8A1, COLEC12, COLQ, COMT, COPRS, COQ10A, COQ6, COQ7, COQ8B, CORIN, CORO2A, CORO7/CORO7-PAM16, COX7B, CPA1, CPD, CPOX, CPQ, CPS1, CR1, CRACR2A, CRBN, CREB5, CRH, CRHR1, CRHR2, CRIP1, CRLF1, CRLF2, CRP, CRTAP, CRY1, CRYZ, CSE1L, CSF1R, CSF2, CSF2RA, CSF2RB, CSF3R, CSGALNACT1, CSH1/CSH2, CSK, CSMD1, CSMD3, CSNK2A2, CSPP1, CSRP2, CSRP3, CST3, CSTF2T, CTCF, CTF1, CTGF, CTH, CTNNB1, CTNNBL1, CTNS, CTR9, CTSA, CTSL, CUBN, CUL3, CUX1, CX3CR1, CXCL10, CXCL11, CXCL12, CXCL13, CXCL17, CXCL2, CXCL8, CXCL9, CXCR4, CXXC4, CYB561, CYB5B, CYB5R3, CYBA, CYBB, CYP11A1, CYP11B1, CYP11B2, CYP17A1, CYP19A1, CYP1A1, CYP1B1, CYP21A1P, CYP21A2, CYP26A1, CYP27B1, CYP2C19, CYP2C9, CYP2D6, CYP2E1, CYP2J2, CYP3A4, CYP3A5, CYP3A7, CYP4A11, CYP4B1, CYP4F2, CYP51A1, CYP7A1, DAAM2, DAB2, DAD1, DBF4, DBH, DBNDD2, DBP, DCAF8, DCC, DCDC2, DCHS1, DCK, DCUN1D5, DDAH1, DDB2, DDC, DDR1, DDR2, DDX1, DDX60L, DFFA, DGCR2, DGCR8, DGKB, DGUOK, DHFR, DHRS1, DHX35, DICER1, DICER1-AS1, DIO2, DIS3L2, DISP2, DLG2, DLGAP3, DLL4, DNAH10, DNAH11, DNAJB11, DNAJB6, DNAJB9, DNAJC22, DNAJC3, DNASE1L3, DNASE2, DNMT3A, DOCK10, DOCK6, DOK6, DPP9, DPYSL3, DRD1, DRD2, DRD3, DRD5, DROSHA, DSC2, DSCAM, DSP, DTX3, DUOX2, DUOXA1, DUOXA2, DUSP18, DUSP8, DYRK1B, E2F3, EARS2, EBF1, EBI3, ECE1, EDA, EDA2R, EDARADD, EDEM3, EDN1, EDNRA, EDNRB, EFCAB13, EFTUD2, EGF, EGFL7, EGFR, EGLN1, EGLN3, EIF2AK1, EIF2AK3, EIF2AK4, EIF4EBP1, EIF4G1, ELAC2, ELK1, ELN, ELP2, EMC8, EMILIN1, EMILIN2, ENG, ENPP1, EOGT, EP300, EPAS1, EPB41, EPCAM, EPHA4, EPHX1, EPO, EPOR, ERAP1, ERAP2, ERBB2, ERCC1, ERCC2, ERCC3, ERCC4, ERCC5, ERCC6, ERCC6L2, ERCC8, ERFE, ERG, ERGIC3, ERMAP, ERVFRD-1, ERVW-1, ESR1, ESR2, ESYT2, ETNK1, EVC2, EVI5, EVX1, EXD3, EXOC2, EXOC3L2, EXOC4, EXOC8, EYA1, EZH2, F10, F11, F12, F13A1, F2, F2R, F3, F5, F7, F8, FAAH, FABP2, FABP4, FADS1, FAF2, FAM107A, FAM109B, FAM114A2, FAM13A, FAM155A, FAM174A, FAM221A, FAM49A, FAM69B, FANCB, FANCC, FAP, FARP1, FARS2, FAS, FASLG, FAT4, FAXDC2, FBLN1, FBN1, FBN2, FBXL2, FBXO32, FBXW7, FCAR, FCGR1A, FCGR1B, FCGR2A, FCGR2B, FCGR2C, FCGR3A/FCGR3B, FDPS, FECH, FERMT1, FGA, FGB, FGF1, FGF10, FGF19, FGF5, FGFR1, FGFR2, FGFR3, FGFR4, FGGY, FH, FHIT, FHL1, FHL5, FIBIN, FIG4, FIGLA, FITM2, FKBP1A, FKBP1B, FKBP2, FLNA, FLT1, FLT3, FLT4, FMN2, FMO2, FMO3, FMR1, FN1, FNDC1, FNDC3B, FOS, FOXC1, FOXC2, FOXD4L4/FOXD4L5, FOXE1, FOXF1, FOXL2, FOXN4, FOXO3, FOXP1, FOXP3, FOXS1, FPR1, FPR2, FPR3, FRK, FRMD4B, FRMD5, FSTL3, FTH1, FTL, FTO, FURIN, FUT11, FXYD1, FZD4, G6PC, G6PC3, G6PD, GAB1, GABARAP, GABRA1, GABRA2, GABRA3, GABRA4, GABRA5, GABRA6, GABRB1, GABRB2, GABRB3, GABRD, GABRE, GABRG1, GABRG2, GABRG3, GABRP, GABRQ, GABRR1, GABRR2, GABRR3, GAL, GALNT13, GALNT14, GALNT18, GALNT2, GALR1, GALR2, GANAB, GATA1, GATA2, GATA3, GATA4, GATA5, GATA6, GBA, GBA2, GBA3, GBE1, GBP2, GC, GCC1, GCC2, GCDH, GCG, GCH1, GCK, GCKR, GCNT1, GDF15, GDF2, GDNF, GEMIN2, GFAP, GFI1, GFI1B, GFM1, GFRA1, GGCX, GGT1, GGT2/LOC102724197, GGT5, GGT6, GGT7, GGTLC1, GGTLC2, GH1, GH2, GHR, GHRH, GHRL, GIPR, GJA4, GJC1, GLA, GLIS2, GLIS3, GLO1, GLP1R, GLRA1, GLRB, GLS, GMFB, GNAI2, GNAI3, GNAS, GNB3, GNG13, GNG5, GNPAT, GOLGA1, GOLPH3, GOPC, GOSR2, GOT1, GOT1L1, GOT2, GP1BA, GP1BB, GPAM, GPC2, GPC3, GPER1, GPHN, GPIHBP1, GPM6B, GPR101, GPT, GPT2, GPX1, GPX2, GPX3, GPX4, GPX5, GPX6, GPX7, GPX8, GRB14, GRB2, GREB1, GREM1, GRHPR, GRIA1, GRIA4, GRIK1, GRIK2, GRIK3, GRIK4, GRIK5, GRIN1, GRIN2A, GRIN2B, GRIN2C, GRIN2D, GRIN3A, GRIN3B, GRINA, GRK2, GRK4, GRK5, GRM5, GRM8, GRN, GRP, GRPR, GSDMB, GSN, GSR, GSS, GSTA1, GSTA3, GSTK1, GSTM1, GSTM2, GSTM5, GSTP1, GSTT1, GSTT2/GSTT2B, GSTZ1, GTPBP1, GUCA2B, GUCY1A2, GUCY1A3, GUCY1B3, GUK1, GXYLT1, GYPA, GYPB, GYPC, GYS1, GZMB, H19, H3F3A/H3F3B, HACE1, HADHA, HADHB, HAGH, HAVCR1, HBA1/HBA2, HBB, HBBP1, HBD, HBE1, HBEGF, HBG1, HBG2, HBQ1, HBS1L, HBZ, HCRT, HCRTR1, HCRTR2, HDC, HELQ, HELZ, HENMT1, HES1, HFE, HGC6.3, HGD, HGFAC, HHAT, HHEX, HIF1A, HIGD1B, HIST1H4H, HIST1H4J, HLA-A, HLA-B, HLA-C, HLA-DMA, HLA-DMB, HLA-DOA, HLA-DOB, HLA-DPA1, HLA-DPB1, HLA-DPB2, HLA-DQA1, HLA-DQA2, HLA-DQB1, HLA-DQB2, HLA-DRA, HLA-DRB1, HLA-DRB3, HLA-DRB4, HLA-DRB5, HLA-E, HLA-F, HLA-G, HMBS, HMG20A, HMGA2, HMGB1, HMGCR, HMGN1, HMOX1, HNF1A, HNF1B, HNF4A, HNRNPU, HOXA2, HOXA3, HOXA4, HOXD10, HOXD3, HOXD9, HPD, HPR, HPRT1, HPSE, HPSE2, HPX, HRAS, HRG, HRH1, HS3ST4, HS6ST1, HSD11B1, HSD11B2, HSD17B3, HSD3B1, HSD3B2, HSD3B7, HSP90AA1, HSP90AB1, HSP90B1, HSPA1L, HSPB2, HSPD1, HSPG2, HTR2A, HTR2B, HTR2C, HTR3B, HTR3E, HTR4, HTRA1, HTT, HUWE1, HYLS1, ICAM1, ICAM2, ICAM4, IDE, IDH1, IDH2, IDUA, IFI16, IFI30, IFIT3, IFITM1, IFITM2, IFNA1/IFNA13, IFNA10, IFNA14, IFNA16, IFNA17, IFNA2, IFNA21, IFNA4, IFNA5, IFNA6, IFNA7, IFNA8, IFNAR1, IFNAR2, IFNB1, IFNE, IFNG, IFNGR1, IFNGR2, IFNK, IFNL3, IFNW1, IFT172, IFT27, IFT74, IGF1, IGF1R, IGF2, IGF2BP2, IGFBP3, IGFBP5, IKBKAP, IKBKG, IL10, IL10RB, IL11, IL12A, IL12B, IL13, IL16, IL17A, IL17RA, IL17RB, IL18, IL1A, IL1B, IL1R1, IL1RN, IL2, IL23R, IL24, IL25, IL2RA, IL2RB, IL2RG, IL31RA, IL3RA, IL4, IL4R, IL5, IL5RA, IL6, IL6R, IL6ST, IL7R, ILK, IMPDH1, IMPDH2, INF2, ING4, INHA, INHBA, INMT, INO80B, INPP5A, INPP5E, INS, INSL3, INSR, INTS3, INTS8, INVS, IPO7, IQCB1, IRAK1, IRAK2, IREB2, IRF2BP2, IRF4, IRF5, IRF7, IRF8, IRS1, IRS2, ISX, ITGA11, ITGA2, ITGA2B, ITGA3, ITGA4, ITGA8, ITGAM, ITGAV, ITGB2, ITGB3, ITGBL1, ITIH4, ITLN1, ITPKB, ITPR1, ITPR2, ITPR3, ITSN1, IVD, IYD, JAG1, JAK1, JAK2, JARID2, JAZF1, JMJD1C, JUNB, JUP, KALRN, KAZN, KCNA1, KCNA4, KCNA5, KCNB1, KCNE1, KCNE2, KCNH1, KCNH2, KCNIP1, KCNIP3, KCNJ11, KCNJ2, KCNJ5, KCNJ8, KCNK16, KCNK3, KCNK9, KCNMA1, KCNMB1, KCNN3, KCNN4, KCNQ1, KCNQ1DN, KCNS1, KCTD1, KCTD15, KDELR2, KDR, KEL, KERA, KHDC3L, KHDRBS1, KIAA0040, KIAA0556, KIAA0586, KIAA0753, KIAA1147, KIF14, KIF17, KIF1A, KIF1B, KIF1BP, KIF7, KIT, KL, KLF1, KLF12, KLF14, KLF15, KLF2, KLF5, KLF6, KLF7, KLF9, KLHL1, KLHL24, KLHL3, KLHL42, KLHL8, KLK1, KLK11, KLKB1, KLRB1, KMT2D, KNG1, KRAS, KREMEN1, KRIT1, KRT10, KRT18, KRT23, KRT8, KYNU, L3MBTL3, LACTB, LAMA2, LARS2, LASP1, LBR, LCAT, LCK, LCLAT1, LCN2, LDLR, LDLRAP1, LEMD3, LEP, LEPR, LGALS1, LGALS3, LGALS7/LGALS7B, LGALS8, LGALS9B, LHB, LHCGR, LHX4, LIF, LIFR, LILRA3, LIMK1, LIN28B, LINC00393, LINC00476, LINC00890, LINC00922, LINC00937, LINC01411, LIPA, LIPC, LIPG, LIPT1, LMBRD1, LMF1, LMNA, LMO1, LMO3, LMOD1, LMX1B, LNX2, LOC100287290, LOC100507477, LOC101927026, LOC102724788/PRODH, LOC105370982, LOC105375366, LOC105375911, LOC105377700, LOC105378010, LOC642361, LONRF3, LOR, LOX, LOXL1, LOXL2, LPA, LPAR1, LPAR2, LPL, LRIG1, LRIG2, LRIG3, LRP1, LRP4, LRP6, LRRC14, LRRC26, LRRC3, LRRC34, LRRC8B, LRRCC1, LTA, LTBP3, LTC4S, LUC7L, LUC7L3, LY6E, LYN, LYZ, LZTFL1, LZTS1, MADD, MAEA, MAFB, MAN1A2, MAN2B1, MAOA, MAP2K1, MAP3K1, MAP3K15, MAP3K7, MAP4K3, MAP6, MAPK1, MAPK12, MAPK14, MAPK15, MAPK3, MAPK4, MAPK6, MAPK7, MAPK8, MAPT, MARC1, MARCH10, MARCH2, MARCH6, MARCH8, MARVELD2, MAS1, MAX, MBD5, MBP, MC2R, MC4R, MCF2, MCHR1, MCL1, MCTP2, MDH2, MDM2, MDM4, ME3, MECOM, MEF2A, MEFV, MEGF8, MEI1, MEN1, MERTK, MESDC2, MET, METTL17, MGLL, MGP, MGST1, MGST2, MGST3, MIA3, MICA, MICALL2, MICB, MIER3, MIF, MKKS, MKL2, MKS1, MLH1, MLLT3, MLXIPL, MMAB, MMACHC, MME, MMP1, MMP12, MMP14, MMP2, MMP23B, MMP28, MMP7, MMP8, MMP9, MNX1, MOCOS, MORN1, MOV10, MPC1, MPL, MPO, MR1, MRE11, MRGPRF, MS4A1, MS4A2, MSH2, MSH5, MSH6, MSL2, MSMO1, MSR1, MSRB3, MST1R, MSX2, MT-ATP6, MT-ATP8, MT-CO1, MT-CO2, MT-CO3, MT-CYB, MT-ND1, MT-ND2, MT-ND3, MT-ND4, MT-ND5, MT-ND6, MT-RNR1, MT-RNR2, MT-TC, MT-TE, MT-TF, MT-TH, MT-TI, MT-TK, MT-TL1, MT-TQ, MT-TS1, MT-TS2, MT1B, MTAP, MTHFD1L, MTHFR, MTHFS, MTIF3, MTNR1B, MTO1, MTOR, MTR, MTRR, MTTP, MTX1, MUC1, MUC16, MUC2, MUC21, MUC5B, MUS81, MUSTN1, MVK, MXRA5, MYB, MYBL2, MYBPC1, MYBPC3, MYC, MYCN, MYH10, MYH7, MYH9, MYLIP, MYLK2, MYNN, MYO10, MYO16, MYO1E, MYO3A, MYO3B, MYOC, MYOZ3, MYSM1, NAA15, NAALAD2, NADSYN1, NANOS1, NANOS3, NANS, NAT2, NAT8, NCAM1, NCAPD3, NCKAP5, NCS1, NDFIP2, NDRG2, NDUFA4L2, NDUFS1, NECTIN2, NEDD4L, NEGR1, NEK1, NELFCD, NES, NETO2, NEUROD1, NF1, NFATC3, NFATC4, NFE2, NFIX, NFKB1, NFKBIA, NFU1, NGF, NHLRC3, NISCH, NKRF, NKX2-1, NKX2-5, NKX2-6, NLGN1, NLRP1, NLRP13, NLRP2, NLRP5, NLRP7, NME1, NMNAT3, NMRK1, NMT2, NNAT, NOC3L, NOD1, NOD2, NOG, NOMO1 , NOP53, NOS1, NOS1AP, NOS2, NOS3, NOTCH1, NOTCH2, NOTCH3, NOTCH4, NOX3, NOX4, NPAS1, NPAS2, NPAS3, NPC1L1, NPHP1, NPHP3, NPHP4, NPHS1, NPHS2, NPM2, NPNT, NPPA, NPPB, NPPC, NPR1, NPR2, NPR3, NPRL3, NPTX2, NPY, NQO1, NR1H2, NR1H4, NR1I2, NR1I3, NR2C2, NR3C1, NR3C2, NR5A1, NRARP, NRAS, NRK, NRTN, NSD3, NSMF, NSUN6, NT5E, NTAN1, NTF4, NTRK1, NTRK2, NTRK3, NUCB2, NUP107, NUP43, NYNRIN, OFD1, OLFML2A, OLFML2B, OLR1, OPCML, OPRD1, OPRK1, OPRM1, OR10G9, OR2T3/OR2T34, OR51B5, OR51B6, OR51L1, ORC5, ORM1, ORM2, OSBPL11, OSBPL7, OSER1, OSMR, OSTM1, OTC, OTOG, OXT, OXTR, P2RX7, P2RY1, P2RY12, P3H1, PABPC4, PAH, PAK1, PAK2, PALB2, PAM16, PARD6A, PARD6B, PARD6G, PARP1, PARP8, PAX8, PBX3, PCDH1, PCDH12, PCM1, PCNA, PCSK9, PCTP, PCYOX1, PDC, PDCD1LG2, PDE10A, PDE11A, PDE12, PDE1B, PDE3A, PDE3B, PDE4A, PDE4B, PDE4C, PDE4D, PDE4DIP, PDE5A, PDE6D, PDE7A, PDE7B, PDE8A, PDE8B, PDGFA, PDGFB, PDGFC, PDGFRA, PDGFRB, PDIA2, PDIA3, PDLIM3, PDPR, PDSS1, PDYN, PDZD2, PDZD4, PEBP1, PEPD, PER1, PER2, PEX14, PFDN2, PGBD1, PGF, PGM2, PGR, PGRMC1, PGS1, PHC1, PHKB, PHOX2B, PHYHIPL, PIAS1, PIAS2, PIBF1, PIGA, PIGF, PIGM, PIGN, PIGQ, PIGT, PIK3CA, PIK3CD, PIK3CG, PIK3R1, PIM1, PINK1, PINX1, PKD1, PKD1L2, PKHD1, PKHD1L1, PLA2G10, PLA2G2A, PLA2G4A, PLA2G5, PLA2G6, PLA2G7, PLAT, PLAU, PLCD3, PLCE1, PLCG1, PLCG2, PLD2, PLEC, PLEKHA4, PLEKHA5, PLEKHA7, PLEKHG1, PLEKHG4, PLEKHM1, PLEKHO2, PLG, PLIN1, PLN, PLOD2, PLS3, PLSCR4, PLTP, PMEPA1, PML, PMP22, PMS1, PNLIPRP3, PNMA3, PNPLA3, POLA1, POLB, POLD1, POLE, POLE2, POLE3, POLE4, POLR3B, POMC, PON1, PON2, PON3, POPDC2, POR, POU3F1, POU5F1, POU6F2, PPARA, PPARD, PPARG, PPARGC1A, PPAT, PPIB, PPIL3, PPM1F, PPOX, PPP1R12C, PPP1R14A, PPP1R3B, PPP2R1A, PPP2R2A, PPP2R2B, PPP3CA, PPP3CB, PPP3CC, PPP3R1, PPP3R2, PRC1, PRCP, PRDX6, PRELID2, PRELP, PREP, PRIM1, PRIM2, PRIMPOL, PRKACA, PRKAG2, PRKAR1A, PRKAR1B, PRKAR2B, PRKCA, PRKCB, PRKCD, PRKCE, PRKCG, PRKCH, PRKCI, PRKCQ, PRKCZ, PRKD1, PRKD3, PRKG1, PRL, PRMT5, PROK1, PROS1, PROX1, PROZ, PRR5, PRRC2A, PRRG1, PRRT1, PRRT2, PRRX1, PRSS21, PRSS22, PRSS33, PRSS8, PRTN3, PSAP, PSCA, PSD4, PSEN1, PSMA1, PSMA2, PSMA3, PSMA4, PSMA5, PSMA6, PSMA7, PSMA8, PSMB1, PSMB10, PSMB11, PSMB2, PSMB3, PSMB4, PSMB5, PSMB6, PSMB7, PSMB8, PSMB9, PSMC1, PSMC2, PSMC3, PSMC4, PSMC5, PSMC6, PSMD1, PSMD10, PSMD11, PSMD12, PSMD13, PSMD14, PSMD2, PSMD3, PSMD4, PSMD5, PSMD6, PSMD7, PSMD8, PSMD9, PSME1, PSME2, PSME3, PSME4, PSMF1, PSORS1C1, PTEN, PTF1A, PTGDS, PTGER1, PTGER2, PTGER3, PTGER4, PTGES, PTGES2, PTGFR, PTGIR, PTGIS, PTGS1, PTGS2, PTK2, PTK2B, PTPMT1, PTPN11, PTPN14, PTPN22, PTPRD, PTPRE, PTPRO, PTPRT, PUM3, PYGB, RAB1A, RAB23, RAB3GAP1, RABGAP1L, RAC1, RAD17, RAD51, RAD54B, RAF1, RAI14, RANBP1, RANBP3, RAPGEF3, RAPGEF4, RARA, RARB, RARG, RARRES2, RASA1, RASGRP1, RASL10B, RASL12, RASSF1, RB1, RBFOX1, RBFOX3, RBL2, RBM12B, RBM6, RBMS1, RBP4, RBP5, RBPJ, RCOR1, RDH5, RECQL4, RELA, REN, RENBP, REST, RET, RETN, RETNLB, REV1, RFTN2, RGS1, RGS2, RHCE/RHD, RHOA, RHOB, RHOH, RHOJ, RNF182, RNF220, RNLS, RNMT, RNU12, ROBO3, ROBO4, ROCK1, ROCK2, RORA, RORB, ROS1, RPGRIP1L, RPH3A, RPL27, RPL31, RPL32, RPS27A, RPS6KA1, RPS6KB1, RRAD, RRM1, RRM2, RRM2B, RSPO1, RSPO3, RSU1, RTEL1, RUNX1, RUNX1T1, RUNX2, RUNX3, RXFP1, S100A1, S100A11, S100A4, S1PR1, SAA1, SAA2, SAA4, SALL2, SAMD11, SAMD12, SAMD4A, SAP30, SAR1A, SARDH, SARS2, SAT2, SBF2, SBNO1, SCAP, SCARB1, SCG2, SCG5, SCLT1, SCN10A, SCN11A, SCN1A, SCN1B, SCN2A, SCN2B, SCN3A, SCN3B, SCN4A, SCN4B, SCN5A, SCN7A, SCN8A, SCN9A, SCNN1A, SCNN1B, SCNN1D, SCNN1G, SCOC, SCYL2, SDC1, SDC2, SDC3, SDCBP, SDCCAG8, SDF2, SDHA, SDHAF2, SDHB, SDHC, SDHD, SDK1, SEC11A, SEC23B, SEC24D, SELE, SELENBP1, SELENON, SELL, SELP, SEMA3C, SEMA5A, SEMA5B, SEPT4, SERINC2, SERPINA1, SERPINA10, SERPINA12, SERPINA3, SERPINA4, SERPINA5, SERPINA6, SERPINA7, SERPINA9, SERPINC1, SERPINE1, SERPINF1, SERPINF2, SERPINH1, SERTAD1, SESN3, SETD2, SETDB1, SETMAR, SEZ6L, SF1, SF3B1, SFTPA1, SFTPA2, SFTPB, SFTPC, SGCD, SGSH, SH2B3, SH2D2A, SH3YL1, SHANK3, SHB, SHBG, SHC1, SHC2, SHOC2, SHTN1, SIGMAR1, SIRPB1, SIRT3, SIX1, SIX2, SKI, SKIL, SLC11A1, SLC12A1, SLC12A2, SLC12A3, SLC12A4, SLC12A6, SLC12A7, SLC14A1, SLC14A2, SLC15A2, SLC16A10, SLC16A12, SLC18A1, SLC18A2, SLC19A3, SLC20A1, SLC22A2, SLC24A2, SLC24A4, SLC25A5, SLC26A4, SLC26A8, SLC2A10, SLC2A13, SLC2A4, SLC2A9, SLC30A7, SLC30A8, SLC33A1, SLC35A5, SLC37A4, SLC39A10, SLC39A8, SLC40A1, SLC43A1, SLC4A1, SLC4A7, SLC52A2, SLC52A3, SLC5A2, SLC5A5, SLC6A1, SLC6A18, SLC6A2, SLC6A3, SLC6A4, SLC6A5, SLC7A1, SLC7A11, SLC7A3, SLC8A1, SLC9A1, SLCO1A2, SLCO1B1, SLCO4C1, SLIT3, SLITRK4, SLN, SMAD1, SMAD2, SMAD3, SMAD4, SMAD6, SMAD7, SMAD9, SMARCA2, SMARCA4, SMARCAL1, SMARCB1, SMIM10L2B, SMIM11A, SMOC2, SMPD1, SMURF2, SNAP25, SNAPC4, SNCA, SNCAIP, SNRK, SNRPD1, SNTB1, SNX10, SNX16, SNX18, SNX29, SOAT1, SOCS3, SOD1, SOD2, SOD3, SORT1, SOSTDC1, SOX12, SOX6, SP3, SPARC, SPARCL1, SPATA5, SPCS2, SPECC1, SPECC1L, SPHK1, SPOCD1, SPP1, SPPL2A, SPPL2C, SPRED1, SPRY1, SPRY2, SPSB3, SPTBN1, SPTY2D1, SRC, SRCAP, SRD5A2, SREBF2, SRGAP1, SRPK3, SRPRB, SRPX, SRR, SRSF2, SRSF3, SRY, SSTR1, SSTR2, SSTR3, SSTR4, SSTR5, ST3GAL4, ST6GAL1, ST7-AS1, ST8SIA4, STAR, STARD3, STAT3, STAT4, STAT5A, STAT5B, STAT6, STK11, STK17A, STK19, STK39, STMN1, STMN2, STN1, STOX1, STRN3, SUGCT, SULT2B1, SUZ12P1, SV2B, SYNCRIP, TAAR1, TACC1, TAF13, TAGLN, TAMM41, TAOK2, TARDBP, TARP, TAS2R1, TAS2R20, TAS2R38, TAX1BP3, TBC1D1, TBCD, TBL1Y, TBPL2, TBX1, TBX4, TBX5, TBXA2R, TCF12, TCF3, TCF7L2, TCIRG1, TCTN1, TCTN2, TCTN3, TEAD2, TEF, TEKT4, TERC, TERT, TESC, TET2, TF, TG, TGFA, TGFB1, TGFB1I1, TGFB2, TGFB3, TGFBI, TGFBR1, TGFBR2, TGFBR3, TH, THADA, THBD, THBS1, THBS2, THNSL1, THPO, THRA, THRB, TIE1, TIMD4, TIMP1, TIMP2, TIMP3, TIMP4, TJP2, TLE3, TLE4, TLR4, TLR5, TLR7, TLR9, TM9SF2, TMED5, TMEM107, TMEM119, TMEM127, TMEM129, TMEM130, TMEM135, TMEM138, TMEM140, TMEM173, TMEM200B, TMEM200C, TMEM204, TMEM216, TMEM231, TMEM237, TMEM259, TMEM26, TMEM47, TMEM52B, TMEM63C, TMEM67, TMEM70, TMEM97, TMOD1, TMPO, TMSB15B, TMX1, TNC, TNF, TNFAIP3, TNFAIP6, TNFRSF11A, TNFRSF11B, TNFSF10, TNFSF11, TNFSF13B, TNFSF15, TNFSF4, TNNC1, TNNC2, TNNT2, TNR, TNXB, TOLLIP, TOP1, TOP2A, TOP2B, TOPBP1, TOR2A, TOX, TP53, TP53INP1, TP63, TP73, TP73-AS1, TPM1, TPM2, TPM3, TPM4, TPO, TPR, TPSAB1/TPSB2, TPSD1, TPSG1, TRAF3IP1, TRAF6, TRAM1, TRAM2, TRAPPC10, TRAPPC3, TREM1, TREX1, TRIB1, TRIM32, TRIM69, TRIM72, TRIO, TRMT11, TRMU, TRPA1, TRPC6, TRPM2, TRPS1, TRPT1, TRPV1, TRPV4, TSHB, TSHR, TSHZ2, TSLP, TSNAXIP1, TSPAN2, TSPAN8, TSPOAP1, TTC12, TTC21B, TTC25, TTC3, TTC39B, TTC39C, TTC8, TTF2, TTK, TTR, TUBA1A, TUBA1C, TUBA3C/TUBA3D, TUBA4A, TUBA8, TUBB1, TUBB2A, TUBB3, TUBB4A, TUBB4B, TUBD1, TUBE1, TUBG1, TUBG2, TULP4, TWNK, TXN2, TXNDC15, TYK2, TYMS, U2AF1/U2AF1L5, UBA52, UBASH3B, UBB, UBC, UBD, UBE2E2, UBE2K, UBE2L3, UBE4B, UBFD1, UBP1, UBQLN1, UCKL1, UCP1, UCP2, UFC1, UGCG, UGGT2, UGT1A1, UGT2B7, UHRF1BP1L, ULK4, UMOD, UNK, UPK3A, UQCRB, UROD, UROS, USB1, USP21, USP8, USP9X, UTP14C, UTP4, UTRN, UTS2, VAC14, VANGL1, VANGL2, VAV2, VAV3, VCAM1, VCAN, VCL, VDR, VEGFA, VEGFB, VEGFC, VEGFD, VEPH1, VGF, VHL, VIM, VIP, VKORC1, VLDLR, VNN1, VPS26A, VPS33B, VTN, VWA3B, VWF, WASF3, WDPCP, WDR19, WDR27, WDR35, WDR5, WFS1, WISP2, WNK1, WNK4, WNT5A, WRN, WT1, WT1-AS, WWC1, XBP1, XDH, XPNPEP2, XPNPEP3, XPO5, XRCC3, XRCC5, XRN1, XYLT1, XYLT2, YBX3, YPEL1, YWHAQ, YWHAZ, YY1AP1, ZBED3, ZBTB16, ZBTB17, ZBTB41, ZC3H13, ZC3HC1, ZDHHC15, ZDHHC21, ZDHHC8, ZFAND3, ZFAND6, ZFHX4, ZFP57, ZFP90, ZFYVE27, ZHX2, ZKSCAN1, ZKSCAN8, ZMIZ1, ZMPSTE24, ZMYM2, ZNF117, ZNF133, ZNF154, ZNF207, ZNF219, ZNF230, ZNF236, ZNF266, ZNF274, ZNF280B, ZNF32, ZNF33B, ZNF343, ZNF362, ZNF423, ZNF516, ZNF525, ZNF532, ZNF540, ZNF563, ZNF595, ZNF614, ZNF626, ZNF648, ZNF652, ZNF662, ZNF664, ZNF671, ZNF7, ZNF701, ZNF717, ZNF770, ZNF800, ZNF816, ZNF823, ZNF827, ZNF83, ZNHIT6, ZXDC |
| **Preeclampsia/eclampsia**  584 affected genes | ABCF2, ABHD4, ABLIM1, ACACB, ACBD3, ACMSD, ACOX1, ACOX2, ACTG2, ACTR5, ACTR6, ACVR1C, ACVR2A, ADAMTS13, ADCY6, ADGRF5, ADGRL1, ADH1A, ADIRF, ADRA1A, ADRA1B, ADRA1D, ADRA2A, ADRA2B, ADRA2C, ADRB1, ADRB2, ADRB3, AFTPH, AGER, AGT, AGTR1, AGTR2, ALDH3A2, ALDH8A1, ALG8, ALKBH3, AMD1, ANGPT1, ANGPTL2, ANKRD29, ANKRD55, AOC3, APBB3, APOBEC3B, APOL3, APTX, ARHGEF10, ARHGEF15, ARHGEF19, ARHGEF25, ARL5B, ARL8B, ASMTL, ASPN, ATF6, ATL3, ATMIN, ATP2A2, ATP2B3, ATP2C1, ATP4A, ATP4B, ATP6V0E2-AS1, ATP6V1A, ATP8B2, BAZ1A, BNIP2, BOK, BORA, BSCL2, BTBD7, BZW1, C10orf10, C12orf49, C16orf45, C18orf8, C1GALT1, C1QTNF5, C1S, C3, C3orf18, C4orf33, C7orf26, CALCOCO1, CALML4, CAND1, CASP8, CAV3, CBFB, CBLN1, CBX7, CCBE1, CCDC117, CCDC136, CCDC50, CCDC74A, CCDC74B, CCDC81, CCDC85A, CCL15, CCR5, CD46, CDC7, CDH13, CDH17, CEP164, CFAP69, CFH, CFI, CGA, CGB1/CGB2, CGB3 , CGB7, CHM, CHN2, CIART, CLCN6, CLCNKA, CLDN23, CLEC14A, CLIC2, COL12A1, COL16A1, COL8A1, COMT, COQ10A, CORIN, CORO2A, CPA1, CPD, CSH1/CSH2, CST3, CSTF2T, CXCL10, CXCL13, CXXC4, CYB5B, CYB5R3, CYP11A1, CYP11B1, CYP11B2, CYP2E1, CYP2J2, DAAM2, DBNDD2, DBP, DCAF8, DCHS1, DCUN1D5, DHFR, DHX35, DICER1-AS1, DLG2, DNAJB11, DNAJB6, DNAJB9, DNAJC3, DNMT3A, DOCK6, DOK6, DPYSL3, DSC2, DTX3, DUSP18, EARS2, EGFL7, EGFR, EIF2AK3, ELAC2, ENG, EP300, EPHX1, ERAP1, ERAP2, ERVFRD-1, ERVW-1, ESR1, F10, F2, F3, F5, FAF2, FAM109B, FAM49A, FAM69B, FANCB, FAS, FAXDC2, FBXO32, FERMT1, FGGY, FHL1, FHL5, FIBIN, FIGLA, FKBP2, FLT1, FMO2, FNDC1, FNDC3B, FOXC1, FOXL2, FOXN4, FOXP3, FOXS1, FUT11, FXYD1, FZD4, GALNT18, GCNT1, GDF15, GH2, GJA4, GLO1, GMFB, GNAI3, GOLGA1, GPC2, GPIHBP1, GPX1, GPX2, GPX3, GPX4, GPX5, GPX6, GPX7, GPX8, GRB2, GSN, GSR, GSTA1, GSTA3, GSTK1, GSTM1, GSTM2, GSTP1, GSTT1, GSTT2/GSTT2B, GSTZ1, GUCY1A3, GUK1, GXYLT1, HADHA, HADHB, HAGH, HENMT1, HES1, HIGD1B, HLA-G, HMGCR, HMOX1, HOXA2, HOXA4, HOXD10, HOXD3, HOXD9, HPSE, HSPB2, HUWE1, IFITM1, IFITM2, IL11, IL6ST, ILK, INHA, INHBA, INMT, INO80B, INTS3, ITGA11, ITPKB, KCNA4, KCNH1, KCNIP3, KCTD15, KDR, KHDC3L, KIAA0040, KIAA1147, KLF15, KLF5, KLF9, KREMEN1, KRT23, KYNU, LBR, LDLR, LGALS1, LHCGR, LINC00890, LMBRD1, LMF1, LMO3, LMOD1, LNX2, LOC100507477, LOC642361, LONRF3, LRIG1, LRRC14, LUC7L, MAN1A2, MAOA, MAP6, MARCH2, MARVELD2, MBD5, MCL1, ME3, MET, METTL17, MGP, MGST1, MGST2, MGST3, MMP23B, MMP28, MOCOS, MPO, MRGPRF, MSMO1, MSRB3, MTHFR, MTHFS, MUS81, MUSTN1, MXRA5, MYNN, MYO10, MYO16, MYOC, NAA15, NAALAD2, NADSYN1, NANS, NCAPD3, NCS1, NDFIP2, NDUFA4L2, NETO2, NFATC4, NFIX, NGF, NKRF, NLGN1, NLRP1, NLRP2, NLRP5, NLRP7, NMNAT3, NOD1, NOS2, NOS3, NOTCH3, NOTCH4, NOX4, NPHP4, NPNT, NPPA, NPTX2, NR1H2, NR3C1, NR3C2, NRARP, NSD3, NSMF, NTRK3, NUCB2, OLFML2A, OLFML2B, OSBPL11, OSBPL7, OXT, OXTR, P2RY12, PAK1, PARD6A, PARD6B, PARD6G, PARP1, PCDH12, PDE12, PDE1B, PDLIM3, PDZD4, PGBD1, PGF, PGM2, PGR, PHKB, PHYHIPL, PIGA, PIGF, PIGN, PIK3CG, PLA2G2A, PLA2G5, PLA2G7, PLCG1, PLEKHA4, PLEKHA5, PLEKHG4, PMEPA1, PMP22, PNMA3, POLR3B, POPDC2, PPARG, PPM1F, PPP1R12C, PPP1R14A, PPP2R2A, PPP3CB, PRDX6, PRELP, PROS1, PRRG1, PRRT2, PRRX1, PSCA, PTGER1, PTGER2, PTGER3, PTGER4, PTGIR, PTGIS, PTGS1, PTGS2, PTPRD, PUM3, RAB1A, RANBP3, RARRES2, RASL12, RBP5, RDH5, REN, RFTN2, ROBO3, ROBO4, RPL27, RSPO1, RUNX1T1, SALL2, SCG2, SCN10A, SCN1A, SCN4B, SCYL2, SDF2, SEC23B, SEC24D, SELENBP1, SELENON, SEMA5A, SEMA5B, SEPT4, SERPINA3, SERPINC1, SERPINE1, SETDB1, SETMAR, SGCD, SHANK3, SHC2, SHTN1, SKIL, SLC12A1, SLC16A10, SLC24A2, SLC26A8, SLC33A1, SLC39A10, SLC39A8, SLC43A1, SLC6A1, SLC6A2, SLC7A3, SLCO4C1, SLIT3, SLITRK4, SLN, SMIM10L2B, SMIM11A, SNCA, SNRPD1, SNTB1, SOD2, SOX12, SP3, SPARC, SPARCL1, SPCS2, SPOCD1, SPPL2A, SRCAP, SRPRB, SRPX, ST7-AS1, ST8SIA4, STOX1, SULT2B1, SYNCRIP, TACC1, TAOK2, TAS2R1, TEAD2, TEF, TGFB1, TGFB1I1, THBS2, THNSL1, TM9SF2, TMED5, TMEM119, TMEM129, TMEM130, TMEM135, TMEM140, TMEM200B, TMEM204, TMEM26, TMEM47, TMEM52B, TMEM97, TMOD1, TMSB15B, TMX1, TNC, TNF, TNFSF13B, TNNC1, TNNC2, TP73-AS1, TPR, TRAM1, TRAPPC10, TRIM69, TRMU, TRPT1, TSNAXIP1, TSPAN2, TSPOAP1, TTC25, TTK, UBASH3B, UBE2K, UBFD1, UCKL1, UHRF1BP1L, USP21, UTP14C, VANGL2, VDR, VEGFA, VEGFB, VEGFC, VPS33B, WDR19, WDR27, WISP2, XBP1, XPNPEP2, YBX3, YPEL1, ZBTB16, ZBTB41, ZFP57, ZFYVE27, ZNF133, ZNF154, ZNF219, ZNF362, ZNF525, ZNF532, ZNF563, ZNF614, ZNF662, ZNF770, ZNF800, ZNF816, ZNF823, ZNF827 |
| **Thrombophilia**  25 affected genes | CBS/CBSL, CYP2C9, F10, F11, F13A1, F2, F5, F8, F9, FGB, HABP2, HRG, LMNA, MTHFR, PROC, PROS1, PROZ, PTAFR, RNF212, SERPINC1, SERPIND1, TFPI, THBD, THPO, VKORC1 |
| **Gene Panel** | **Variants included in genes** |
| **Agents acting on the renin-angiotensin system pathway**  32 genes | ACE, CMA1, CTSG, REN, MAS1, ATP6AP2, AGT, ACE2, AGTR1, AGTR2, CYP11B2, KNG1, NR3C2, NOS3, MME, TGFB1, BDKRB1, BDKRB2, MAPK1, MAPK3, MRGPRD, CPA3, ENPEP, ANPEP, KLK1, KLK2, LNPEP, CTSA, PRCP, PREP, NLN, THOP1 |
| **Antigen Presentation- Folding assembly and peptide loading of class I MHC**  25 genes | B2M, CALR, CANX, PDIA3, HLA-A, HLA-B, HLA-C, HLA-E, HLA-F, HLA-G, HLA-H, HSPA5, SEC13, TAP1, TAP2, TAPBP, SEC24C, SEC24D, SEC24B, SEC23A, SEC24A, SEC31A, SAR1B, ERAP1, ERAP2 |
| **Cell adhesion endothelial cell contacts by non-junctional mechanisms**  40 genes | ACTB, ACTG1, ACTN4, ACTN1, ACTN2, ACTN3, CDH2, COL1A1, COL1A2, COL4A1, COL4A2, COL4A3, COL4A4, COL4A5, COL4A6, CTNNA1, CTNNA2, CTNNB1, CTNND1, FN1, ITGA6, ITGA1, ITGA2, ITGA3, ITGA5, ITGAV, ITGB1, ITGB3, ITGB4, ITGB5, JUP, LAMA4, LAMB1, LAMC1, PECAM1, VTN, MAGI1, CTNNA3, ESAM, LAMA1 |
| **DNA double-strand break repair**  315 genes | MRE11A, RAD50, CHEK1, ATM, ATR, BRCA1, MDC1, NBN, RBBP8, BARD1, BLM, DNA2, H2AFX, HIST1H2BD, HIST1H2BB, HUS1, RAD1, RAD9A, RAD17, RFC2, RFC3, RFC4, RFC5, RPA1, RPA2, RPA3, TOP3A, TP53BP1, UBE2N, UBE2V2, SUMO1, WHSC1, WRN, HIST3H3, HIST1H4I, HIST1H2BG, HIST1H2BL, HIST1H2BN, HIST1H2BM, HIST1H2BF, HIST1H2BE, HIST1H2BH, HIST1H2BI, HIST1H2BC, HIST1H2BO, HIST2H2BE, HIST1H4A, HIST1H4D, HIST1H4F, HIST1H4K, HIST1H4J, HIST1H4C, HIST1H4H, HIST1H4B, HIST1H4E, HIST1H4L, HIST2H4A, HERC2, HIST1H2BJ, RNF8, EXO1, BRE, KAT5, TOPBP1, BABAM1, PIAS4, UIMC1, BRCC3, RMI1, RHNO1, BRIP1, ATRIP, FAM175A, HIST1H2BK, RMI2, HIST4H4, HIST3H2BB, RAD9B, RNF168, HIST1H2BA, HIST2H4B, LOC102724334, ABL1, CCNA2, CDK2, FEN1, PPP4C, RNF4, RPS27A, SUMO2, UBA52, UBB, UBC, UBE2I, CCNA1, TIMELESS, SIRT6, TIPIN, CLSPN, PPP4R2, PRKDC, TP53, CHEK2, PARP1, BRCA2, ERCC1, XRCC6, LIG3, PCNA, POLD1, POLD2, POLE, POLE2, POLH, RAD51, RAD51C, RAD51B, RAD51D, RAD52, ERCC4, RFC1, XRCC1, XRCC2, XRCC3, XRCC4, XRCC5, PARP2, RAD51AP1, POLD3, POLQ, SPIDR, POLK, RTEL1, POLD4, SLX1B, PALB2, NHEJ1, MUS81, SLX4, EME1, EME2, SLX1A, LOC105369236, GEN1, LIG4, POLL, POLM, DCLRE1C, APBB1, EYA1, EYA2, EYA3, KPNA2, PPP5C, MAPK8, EYA4, SMARCA5, BAZ1B, KDM4A, PAXIP1, KDM4B, TDP2, RIF1, TDP1, CCNB1, CDK1, CDC25C, SFN, WEE1, YWHAB, YWHAE, YWHAG, YWHAH, YWHAZ, YWHAQ, DNTT, ACTB, ACTL6A, APEX1, CCNH, CDK7, CETN2, ERCC8, DDB1, DDB2, EP300, ERCC2, ERCC3, ERCC5, ERCC6, FANCA, FANCC, FANCD2, FANCE, FANCB, FANCF, FANCG, GPS1, MSH6, GTF2H1, GTF2H2, GTF2H3, GTF2H4, HMGN1, LIG1, MGMT, MLH1, MNAT1, MPG, MSH2, MSH3, MUTYH, NFRKB, NTHL1, OGG1, PMS2, POLB, POLR2A, POLR2B, POLR2C, POLR2D, POLR2E, POLR2F, POLR2G, POLR2H, POLR2I, POLR2J, POLR2K, POLR2L, RAD23A, RAD23B, REV3L, SUMO3, TCEA1, TDG, UBA7, UBE2B, UFD1L, UNG, USP1, VCP, XPA, XPC, YY1, TRIM25, USP7, ELL, CUL4B, CUL4A, PARG, COPS3, PIAS1, RUVBL1, MBD4, USP10, UBE2L6, COPS2, CHD1L, ISG15, AQR, KIAA0101, DCLRE1A, RBX1, PIAS3, MCRS1, PPIE, MAD2L2, COPS8, ASCC3, COPS6, COPS5, POLI, PNKP, FAN1, SMUG1, RCHY1, ZBTB32, PRPF19, UBE2T, TFPT, COPS7A, ASCC1, COPS4, REV1, DTL, INO80, RNF111, INO80D, FANCL, FANCI, NPLOC4, RAD18, XAB2, ISY1, UVSSA, FANCM, COPS7B, DCLRE1B, NEIL1, ACTR5, FAAP100, INO80B, SPRTN, ASCC2, USP45, FAAP24, ZNF830, ACTR8, ALKBH2, USP43, INO80C, FAAP20, NEIL2, INO80E, ALKBH3 |
| **Complement and coagulation cascades**  80 genes | A2M, SERPINC1, BDKRB1, SERPING1, C1QA, C1QB, C1QC, C1R, C1S, C2, C3, C3AR1, C5AR1, C6, C7, C8G, C9, CPB2, CR1, CR2, F2, F2R, F3, F5, F7, F8, F9, F10, F12, F13B, FGB, SERPIND1, KLKB1, KNG1, CD46, SERPINE1, SERPINA5, SERPINA1, PLAT, PLAU, PLAUR, PLG, SERPINF2, PROC, PROS1, MASP1, TFPI, THBD, VWF, MASP2, APOA2, PROCR, VSIG4, CLU, CD55, CFD, F2RL2, F11, F13A1, FGA, FGG, CFH, CFI, ITGAM, ITGAX, ITGB2, MBL2, SERPINB2, BDKRB2, CFB, C4A, C4B, C4BPA, C4BPB, C5, C8A, C8B, VTN, F2RL3, CD59 |
| **Epoxide Hydrolase Pathway**  22 genes | VKORC1, F2, F7, BGLAP, CALU, CYP4F2, EPHX1, F10, F9, GAS6, GGCX, MGP, PROC, PROS1, PROZ, COX8A, CYP2C8, CYP2C9, CYP2J2, EPHX2, GSTP1, COX5A |
| **Genes published in Moses et al.**  37 genes | TGFB1, INHBA, IFNG, IL6, AGT, VEGFA, ACVR1C, COL4A2, COL4A1, INHBB, ERAP2, ERAP1, INHA, ACVR2A, ACVR1, LNPEP, SMAD7, XIAP, SHH, FST, CYP17A1, AVP, SLC2A4, OXT, TNF, INS, ENG, IL10, EDN1, NOS3, NOS2, PTGS2, MMP2, CDH1, MMP9, KDR, FLT1 |
| **Myometrial Relaxation and Contraction Pathways**  213 genes | ADCY1, CACNB3, GNB2, ITPR1, PRKCA, RGS4, YWHAZ, GNG13, ADM, CRHR1, IGFBP4, OXTR, CREB3, ADRA1B, ATP2B2, CASQ2, GJB2, PLN, ADCY2, CALM1, GNB3, ITPR2, PRKCB, RGS7, RGS5, GNG2, ATF1, LPAR1, IGFBP5, PDE4B, CORIN, ADRA1A, ATP2B3, CHRM1, GJB3, SLC8A3, ADCY3, CALM2, GNG3, ITPR3, PRKCD, RGS10, RGS20, GNG12, ATF3, ETS2, IGFBP6, PDE4D, GPR182, ADRB1, CACNA1A, CHRM2, GJB5, CAMK1, ADCY5, CALM3, GNG4, PLCB3, PRKCE, RGS16, RGS11, GNB4, ATF4, FOS, IL1B, PLCD1, ATF5, ADRB2, CACNA1B, CHRM3, GNA11, GJC1, ADCY6, CAMK2A, GNG5, PRKACA, PRKCG, RYR1, RGS9, RGS18, CALCA, GABPA, IL6, PLCG1, MAFF, ADRB3, CACNA1C, CHRM4, GNAI1, GJB6, ADCY7, CAMK2B, GNG7, PRKACB, PRKCH, RYR2, RGS6, GNG8, CALD1, GABPB1, JUN, PLCG2, CRCP, ANXA6, CACNA1D, CHRM5, GNAI2, GJC2, ADCY8, CAMK2D, GNG11, PKIA, PRKD1, RYR3, RGS19, ADCY4, CNN1, GUCA2A, MYL2, SP1, RLN1, ATP1A4, CACNA1E, FKBP1A, GNAI3, GJD2, ADCY9, CAMK2G, GNGT1, PKIB, PRKCQ, SLC8A1, RGS14, ACTA1, CNN2, GUCA2B, MYL4, DGKZ, ACKR3, ATP1B1, CACNA1S, GJA3, GNAO1, GJA9, ARRB1, GJA1, SFN, PRKAR1A, PRKCZ, YWHAB, GNB5, ACTA2, CREB1, GUCY1A3, NFKB1, GSTO1, RXFP1, ATP1B2, CACNB1, GJA4, GNAZ, GJB4, ARRB2, GNAQ, GRK4, PRKAR1B, RGS1, YWHAE, YWHAQ, ACTB, ATF2, IGFBP1, NOS1, RAMP2, MYLK2, ATP1B3, CALR, GJA5, KCNB1, ATP2A2, GNAS, GRK5, PRKAR2A, RGS2, YWHAG, PKIG, ACTC1, ATF6B, IGFBP2, NOS3, RAMP1, RXFP2, FXYD2, CAMK4, GJA8, KCNJ3, ATP2A3, GNB1, GRK6, PRKAR2B, RGS3, YWHAH, RGS17, ACTG1, CRH, IGFBP3, OXT, RAMP3, ADRA1D, ATP2B1, CASQ1, GJB1, KCNJ5 |
| **Transforming growth factor-beta signalling pathway**  103 genes | GDF1, ACVR1, ACVR1B, ACVR1C, ACVR2A, ACVR2B, ACVRL1, AKT1, AKT2, AKT3, ATF2, BAMBI, BMP10, BMP2, BMP4, BMP5, BMP7, GDF2, BMPR1A, BMPR1B, BMPR2, CDC42, CER1, CHRDL1, DAND5, DCN, DPT, ENG, FST, FSTL1, FSTL3, GDF15, GDF3, GDF5, GDF6, GDF9, GDNF, GFRA1, GFRA2, GFRA3, HRAS, INHA, INHBA, INHBB, INHBC, INHBE, KRAS, LEFTY2, MAP2K1, MAP2K2, MAP2K4, MAP2K6, MAPK1, MAPK10, MAPK12, MAPK13, MAPK14, MAPK3, MAPK8, MAPK9, MARK3, MSTN, MTOR, NBL1, NCAM1, NOG, NR2C2, NRAS, NRTN, PAK2, PAK3, PAK4, PAK6, PAK5, PARD6A, PIK3R4, PRKCA, PRKCG, PRKCB, PRKCD, PRKCE, PRKCI, PRKCZ, PRKD3, RAC2, RAF1, RHOA, RPS6KB1, SMAD2, SMAD3, SMAD4, SMAD5, SMURF1, SOST, TDGF1, TGFB1, TGFB2, TGFB3, TGFBR1, TGFBR2, TGFBR3, TMEFF1, TSKU |

**Ingenuity Variant Analysis version 5.0.20171003**

Content versions: CADD (v1.3), CentoMD (-), EVS (ESP6500SI-V2), Allele Frequency Community (2017-07-03), JASPAR (2013-11), Ingenuity Knowledge Base (Narnia 171007.000), Vista Enhancer (2012-07), gnomAD (2.0.1), Clinical Trials (Narnia 171007.000), BSIFT (2016-02-23), TCGA (2013-09-05), PolyPhen-2 (v2.2.2), 1000 Genome Frequency (phase3v5b), Clinvar (2017-06-01), DGV (2016-05-15), COSMIC (v81), ExAC (0.3.1), HGMD (2017.2), PhyloP (2009-11), DbSNP (150), TargetScan (6.2), SIFT4G (2016-02-23)
